# Differentiated adaptive evolution, episodic relaxation of selective constraints, and pseudogenization of umami and sweet taste genes *TAS1Rs* in catarrhine primates

**DOI:** 10.1186/s12983-014-0079-4

**Published:** 2014-10-29

**Authors:** Guangjian Liu, Lutz Walter, Suni Tang, Xinxin Tan, Fanglei Shi, Huijuan Pan, Christian Roos, Zhijin Liu, Ming Li

**Affiliations:** Key Laboratory of Animal Ecology and Conservation Biology, Institute of Zoology, Chinese Academy of Sciences, 1-5 Beichen West Road, , Chaoyang Beijing, 100101 China; University of Chinese Academy of Sciences, Beijing, 100049 China; Primate Genetics Laboratory, German Primate Center, Leibniz Institute for Primate Research, Kellnerweg 4, 37077 Göttingen, Germany; Gene Bank of Primates, German Primate Center, Leibniz Institute for Primate Research, Kellnerweg 4, 37077 Göttingen, Germany; Department of Biomedical Sciences, School of Pharmacy, Texas Tech University Health Sciences Center, 1300 S. Coulter St, Amarillo, TX 79106 USA; Institute of Health Sciences, Anhui University, Hefei, Anhui Province 230601 China; College of Nature Conservation, Beijing Forestry University, Haidian Beijing, 100083 China

**Keywords:** Catarrhine primates, *TAS1Rs*, Adaptive evolution, Positive selection, Episodic relaxation of selective constraints, Pseudogenization

## Abstract

**Background:**

Umami and sweet tastes are two important basic taste perceptions that allow animals to recognize diets with nutritious carbohydrates and proteins, respectively. Until recently, analyses of umami and sweet taste were performed on various domestic and wild animals. While most of these studies focused on the pseudogenization of taste genes, which occur mostly in carnivores and species with absolute feeding specialization, omnivores and herbivores were more or less neglected. Catarrhine primates are a group of herbivorous animals (feeding mostly on plants) with significant divergence in dietary preference, especially the specialized folivorous Colobinae. Here, we conducted the most comprehensive investigation to date of selection pressure on sweet and umami taste genes (*TAS1Rs*) in catarrhine primates to test whether specific adaptive evolution occurred during their diversification, in association with particular plant diets.

**Results:**

We documented significant relaxation of selective constraints on sweet taste gene *TAS1R2* in the ancestral branch of Colobinae, which might correlate with their unique ingestion and digestion of leaves. Additionally, we identified positive selection acting on Cercopithecidae lineages for the umami taste gene *TAS1R1*, on the Cercopithecinae and extant Colobinae and Hylobatidae lineages for *TAS1R2*, and on *Macaca* lineages for *TAS1R3*. Our research further identified several site mutations in Cercopithecidae, Colobinae and *Pygathrix*, which were detected by previous studies altering the sensitivity of receptors. The positively selected sites were located mostly on the extra-cellular region of TAS1Rs. Among these positively selected sites, two vital sites for TAS1R1 and four vital sites for TAS1R2 in extra-cellular region were identified as being responsible for the binding of certain sweet and umami taste molecules through molecular modelling and docking.

**Conclusions:**

Our results suggest that episodic and differentiated adaptive evolution of *TAS1Rs* pervasively occurred in catarrhine primates, most concentrated upon the extra-cellular region of TAS1Rs.

**Electronic supplementary material:**

The online version of this article (doi:10.1186/s12983-014-0079-4) contains supplementary material, which is available to authorized users.

## Background

Umami and sweet tastes are important sensations allowing animals to recognize diets with nutritious proteins and carbohydrates, respectively. In mammals, sweet and umami taste perceptions are conferred by taste receptor cells through the use of G protein-coupled receptors (GPCRs) TAS1R, which are encoded by the *TAS1R* gene family [1,2]. Of these, TAS1R1 and TAS1R2 are expressed in separate taste receptor cells, although both are co-expressed with TAS1R3. The TAS1R1 protein forms a heterodimer with TAS1R3 to form a two-part umami taste receptor, and the TAS1R2 and TAS1R3 heterodimer functions as the sweetness receptor [3,4].

The gene structure of *TAS1R* family is conserved among species [5]. Human *TAS1R* genes span from 3 kb-20 kb with 6 exons and 5 introns, and the cDNA of *TAS1R* genes consist of ~2500 bp. TAS1R proteins are characteristic of seven domains spanning the plasma membrane, which have a large N-terminal extracellular domain composed of the Venus flytrap module (VFTM) and the small cysteine-rich domain (CRD), followed by the transmembrane domain (TMD) and the C-terminal intracellular domain (CID) [6]. These domains are required for recognizing sweeteners and umami compounds, such as aspartame, neotame, monellin, cyclamate, neohesperidin dihydrochalcone, brazzein and L-amino acids [7-12]. Furthermore, studies using mutagenesis, molecular modeling and functional expression have demonstrated multiple potential binding sites in the heterodimeric receptors [8-11,13-19]. For example, it is reported that two amino acid substitutions (A110V and R507Q) in the VFTM domain of TAS1R1 and two substitutions (F749S and R757C) in the TMD domain of TAS1R3, severely impair the response to monosodium glutamate (MSG) in humans [17]. Above all, genetic factors have been shown to play a crucial role in the variability of sensitivity to tastants.

Until recently, analyses of umami and sweet taste receptors have been performed on various domestic and wild animals. For example, *TAS1R1* is a pseudogene in the herbivorous giant panda (family Ursidae) [20,21], and the *TAS1R2* gene is inactivated in cats (family Felidae), vampire bats, chickens, zebra finches, the western clawed frog, and some carnivorous mammals [5,22,23]. However, research on the evolution of sweet and umami taste genes revealed that taste perception of sweet and umami is not be as conserved as previously thought, and the structural integrity of *TAS1R* is sometimes inconsistent with the known functions of these genes and the tastes involved [24]. For example, although *TAS1R2* is a pseudogene in some species of carnivores, some other obligate carnivores (such as ferrets and Canadian otters) still possess an intact *TAS1R2* gene [25]. Moreover, *TAS1R2* is absent in all bird genomes sequenced thus far, irrespective of their diet [21]. These puzzling cases indicate that our understanding of the physiological functions of sweet and umami tastes and/or their receptor genes is far from complete [24].

While most previous studies in mammals focused on the pseudogenization of taste genes which occur mostly in carnivores and species with absolute feeding specialization, omnivores and herbivores were more or less neglected, except for the systematic study of bats and the giant panda [26]. Additionally, it is known that plant foods usually contain much more complex and variable taste-inducing compounds than animal food [27,28]. The taste system of omnivores and herbivores may be more complex than in carnivores, and pseudogenization of taste genes is less possible to occur massively in omnivores and herbivores (except for the giant panda). To thoroughly understand the physiological functions and adaptive evolution of sweet and umami tastes and/or their receptor genes, more comprehensive studies should be performed in omnivorous and herbivorous animals with close phylogenetic relationships.

Catarrhine primates are a group of herbivorous animals (feeding mostly on plants) with a significant divergence in dietary preference. The Colobinae, also called leaf-monkeys, feed predominantly on relatively low-energy leaves and other plant parts [29], and are unique among primates in that they have a complex stomach to permit efficient digestion of leaves [30]. By contrast, the Cercopithecinae feed predominantly on relatively high-energy foods such as fruits, seeds, insects, and vertebrates. Gibbons are fruit-pulp specialists, and the foods eaten by the great apes and modern humans generally include a wide variety of items such as fruits, assorted types of vegetation, bark, seeds, insects and meat, although the great apes are predominantly frugivorous. Consequently, as a group of animals including omnivores and herbivores, catarrhine primates exhibit a certain degree of diet specialization and differentiation, which makes them ideal objects for studying the evolution of sweet and umami taste genes.

As diets have evolved and differentiated during the radiation of catarrhine primates, presumably tastes have responded adaptively in order to maximize energy intake. Species with the highest taste sensitivity for sugars and other soluble nutrients tend to improve foraging efficiency, which could be the target of natural selection. Thus, we predicted that the umami and sweet taste genes of species in catarrhine primates with different diets have experienced variation in selection pressure through evolutionary history. To test this hypothesis, we performed a comprehensive investigation of evolution in *TAS1R* genes for representative species of herbivorous catarrhine primates. Our research aimed to explore possible specific adaptive evolution of *TAS1R* in catarrhine primates with known dietary specializations, and to enrich our understanding of the physiological functions of sweet and umami tastes receptor genes.

## Results

### Characterization of the *TAS1R* genes in catarrhine primates

Complete coding regions of *TAS1R* were obtained from 30 catarrhine primate species. Most of them were highly conservative without premature stop codons or frame shift mutations. In total, 2523 bp, 2517 bp and 2556 bp DNA sequences were generated from six exons of *TAS1R1*, *TAS1R2* and *TAS1R3*, respectively. Additionally, we also downloaded available *TAS1R* sequences of Hominidae from GenBank: all *TAS1R* of *Pan troglodytes*, *Homo sapiens* and *Gorilla gorilla gorilla*; *TAS1R1* and *Tas1r2* of *Pan paniscus*; *TAS1R1* and *TAS1R3* of *Pongo abelii*; *Tas1r2* and *TAS1R3* of *Pongo pygmaeus* (see Additional file 1). Alignments of *TAS1R* revealed a total of 334 (13.24%), 398 (15.81%) and 469 (18.35%) variable sites for *TAS1R1*, *TAS1R2* and *TAS1R3*, respectively. Among these sites, 243, 318 and 414 synonymous mutations were found in *TAS1R1*, *TA1SR2* and *TAS1R3*, respectively. Correspondingly, 185, 263 and 269 non-synonymous mutations were detected in *TAS1R1, TAS1R2* and *TAS1R3*, which revealed the most variable sites but the lowest non-synonymous ratio of *TAS1R3* (see Additional file 2). The alignments of amino acid sequences are provided as Additional files 3, 4 and 5.

### ORF-disrupting mutations in *TAS1R* genes

**Figure 1:**
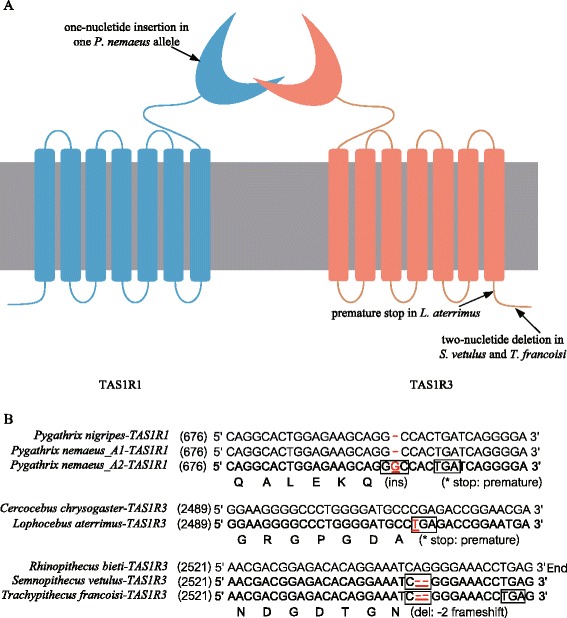
**Schematic of umami receptor structure and ORF-disrupting mutations of** ***TAS1R genes*** **. (A)** Schematic of umami receptor structure. ORF-disrupting mutations are marked. **(B)** ORF-disrupting mutations. A1 and A2 denote the pair of *TAS1R1* alleles of *Pygathrix nemaeus*. The first line of each aligned group is intact sequence. The codon that contains the ORF-disrupting mutation (marked with red and underlined) is indicated by a box.

The open reading frame (ORF) of *TAS1R* was disrupted in some species of Cercopithecidae, including insertions, deletions and transitions (Figure 1). Both of two individuals of *Pygathrix nemaeus* had an allele of *TAS1R1* with an insertion of a G in exon 3 (nucleotide position 695, codon number 232), leading to a frame shift mutation and a premature stop codon. Thus, *TAS1R1* is likely a pseudogene in *P. nemaeus*, resulting in the truncation of the protein in the extracellular N-terminus (VFTM). However, this gene is intact in all other *Pygathrix* species *P. nigripes* and *P. cinerea*, indicating a recent origin of this pseudogene. *Trachypithecus francoisi* and *Semnopithecus vetulus* share a 2-nucleotide deletion at the very end of exon 6 (nucleotide position 2,543-2,544, codon number 848) of *TAS1R3*, leading to three amino acid substitutions and a two amino acid residue shorter C-terminal intracellular domain (CID) in comparison with other species. Thus, this mutation of *TAS1R3* was suggested to occur in the common ancestor of *Semnopithecus* and *Trachypithecus*, which are the closest genera in Colobinae. Interestingly, a premature stop codon was also found in *TAS1R3* of *Lophocebus aterrimus* because of a transition from C to T in exon 6 (nucleotide position 2,512, codon number 838). All these mutations were confirmed by multiple PCR experiments and colonies of different cloning procedures.

### Relaxation of selective constraint on *TAS1R* genes within catarrhine primates

**Figure 2:**
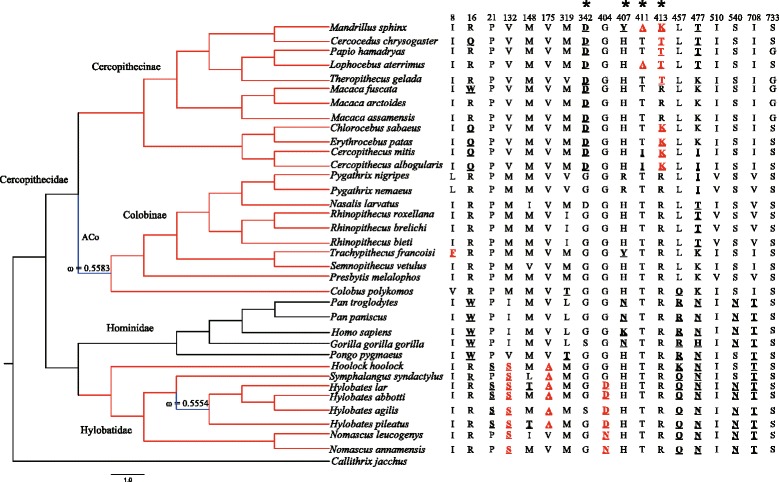
**Radical amino acid changes in selected sites across the catarrhine primates phylogeny of** ***TAS1R2.*** The input tree used for pressure analysis of *TAS1R2* is shown on the left. Positively selected sites detected by site models and branch-site models are shown on the right. Radical changes are shown in bold and underlined, and changes detected by branch-site models are marked in red additionally. *Callithrix jacchus* was used as outgroup, but not included in the CODEML analysis. Lineages under positive selection are marked in red, while lineages experienced relaxation of selective constraints are marked in blue.

**Figure 3:**
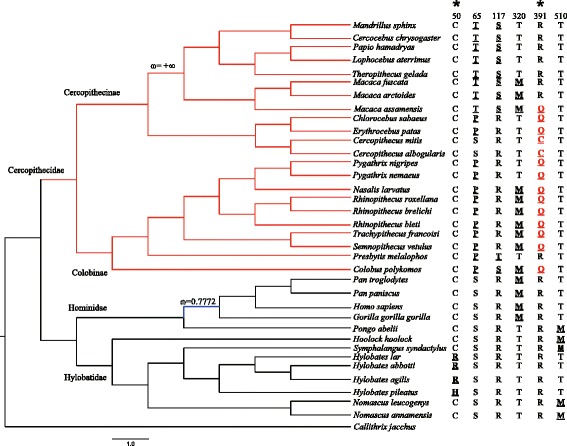
**Radical amino acid changes in selected sites across the catarrhine primates phylogeny of** ***TAS1R1*** **.** The input tree used for pressure analysis of *TAS1R1* is shown on the left. Positively selected sites detected by site models and branch-site models are shown on the right. Radical changes are shown in bold and are underlined, and changes detected by branch-site models are marked in red additionally. *Callithrix jacchus* was used as outgroup, but not included in the CODEML analysis. Lineages under positive selection are marked in red, while lineages experienced relaxation of selective constraints are marked in blue.

In the branch model, the free-ratio model was detected to be significantly different from the one-ratio model only for *TAS1R2*, indicating that *d*_N_/*d*_S_ ratios among lineages of *TAS1R2* were different (Table 1). These ratios varied from 0 (*d*_N_ = 0) to infinity (*d*_S_ = 0). Then, the two-ratio model was used to identify whether lineages with 0.50 < ω <1 (detected by free-ratio) had underwent different selection pressure from other lineages, which was addressed as hypothesis 1 (H1) in Table 1. After comparing with the one-ratio model, we found that selection pressure acting on the ancestral lineage of Colobinae for *TAS1R2* (branch ACo in Figure 2) was significantly different from other lineages (ω_1_ = 0.5583, *p <*0.05, Table 1). We also hypothesized a fixing ω_1_ = 1 for the ancestral lineage of *TAS1R2* in Colobinae (H0, Table 1), and H1 was not significantly supported compared with H0. The analysis revealed that selection constraints were relaxed on *TAS1R2* of ancestral Colobinae, but it was not totally removed. The relaxation of selective constraints was also found in *TAS1R1* of the common ancestor of human, chimpanzee and gorilla (ω_1_ = 0.7772, *p <*0.05, Table 2, Figure 3), and in *TAS1R2* of the common ancestor of the genus *Hylobates* (ω_1_ = 0.5554, *p <*0.05, Table 1, Figure 2).

**Table 1:** **CODEML analyses of selective pattern on the** ***TAS1R2*** **in catarrhine primates**

**Models**	**lnL**	**Compared**	**2ΔLnL**	***P-value***	**Parameter estimates**
***Branch model***					
M0:one-ratio	−7258.8072				ω = 0.1608
M1:free-ratio	−7211.2267	M0 vs. M1	95.1610	*p <* 0.05	
Foreground branch: ancestral Colobinae					
H1: ω_0,_ ω_1_	−7256.0276	M0 vs. H1	5.5592	*p <* 0.05	ω_0_ = 0.1548, ω_1_ = 0.5583
H0: ω_0,_ ω_1_ = 1	−7256.4033	H0 vs. H1	0.7514	*p* > 0.05	ω_0_ = 0.1543, ω_1_ = 1.0000
Foreground branch: ancestral *Hylobates*					
H1: ω_0,_ ω_1_	−7256.8606	M0 vs. H1	3.8932	*p <* 0.05	ω_0_ = 0.1570, ω_1_ = 0.5554
H0: ω_0,_ ω_1_ = 1	−7257.1816	H0 vs. H1	0.6420	*p* > 0.05	ω_0_ = 0.1570, ω_1_ = 1.0000
***Site model***					
M1a	−7149.5623				p_0_ = 0.8609, p_1_ = 0.1391,ω_0_ = 0.0451, ω_1_ = 1.0000
M2a	−7145.1097	M1a vs. M2a	8.9052	*p <* 0.05	p_0_ = 0.8669, p_1_ = 0.1198, p_2_ = 0.0133, ω_0_ = 0.0499, ω_1_ = 1.0000, ω_2_ = 3.6223
M8a	−7149.3577				p_0_ = 0.8725( p_1_ = 0.1275), p = 0.4874, q = 7.5964, ω = 1.0000
M8	−7143.4448	M8a vs. M8	11.8258	*p <* 0.01	p_0_ = 0.9744( p_1_ = 0.0257), p = 0.1750, q = 1.1154, ω = 2.8718
***Branch-site model***					
Lineages of Cercopithecidae					
Null	−7146.1093				p_0_ = 0.8463, p_1_ = 0.0953, p_2a_ = 0.0525 p_2b_ = 0.0059,ω_0_ = 0.0407, ω_1_ = 1.0000 ω_2_ = 1.0000
Alternative	−7144.2553		3.7080	*p <* 0.05	p_0_ = 0.8683, p_1_ = 0.0924, p_2a_ = 0.0354 p_2b_ = 0.0038,ω_0_ = 0.0481, ω_1_ = 1.000 ω_2_ = 2.0345
Lineages of Colobinae					
Null	−7145.5063				p_0_ = 0.8299, p_1_ = 0.1176, p_2a_ = 0.0460 p_2b_ = 0.0065,ω_0_ = 0.0396, ω_1_ = 1.0000,ω_2_ = 1.0000
Alternative	−7143.7154		3.5818	*p <* 0.05	p_0_ = 0.8542, p_1_ = 0.1162, p_2a_ = 0.0261 p_2b_ = 0.0036,ω_0_ = 0.0428, ω_1_ = 1.0000 ω_2_ = 2.4997,
Lineages of Cercopithecinae					
Null	−7148.7133				p_0_ = 0.8512, p_1_ = 0.1292, p_2a_ = 0.0170 p_2b_ = 0.0026,ω_0_ = 0.0440, ω_1_ = 1.0000,ω_2_ = 1.0000
Alternative	−7143.4285		10.5696	*p <* 0.01	p_0_ = 0.8639, p_1_ = 0.1228, p_2a_ = 0.0116 p_2b_ = 0.0017,ω_0_ = 0.0466, ω_1_ = 1.0000 ω_2_ = 5.8766
Lineages of Hylobatidae					
Null	−7149.1766				p_0_ = 0.8489, p_1_ = 0.1326, p_2a_ = 0.0161 p_2b_ = 0.0025,ω_0_ = 0.0442, ω_1_ = 1.000 ω_2_ = 1.0000
Alternative	−7143.6646		11.0240	*p*0.01	p_0_ = 0.8612, p_1_ = 0.1297, p_2a_ = 0.0079, p_2b_ = 0.0012,ω_0_ = 0.0462, ω_1_ = 1.000 ω_2_ = 11.8832

**Table 2:** **CODEML analyses of selective pattern on the** ***TAS1R1*** **in catarrhine primates**

**Models**	**lnL**	**Compared**	**2ΔLnL**	***P-value***	**Parameter estimates**
***Branch model***					
M0:one-ratio	−6347.5127				ω = 0.2486
M1:free-ratio	−6316.3339	M0 vs. M1	62.3576	*p* > 0.05	
Foreground branch: all lineages of Cercopithecidae except for the ancestral branch					
H1: ω_0,_ ω_1_	−6344.8700	M0 vs. H1	5.2854	*p <* 0.05	ω_0_ = 0.3112, ω_1_ = 0.1981
H0: ω_0,_ ω_1_ = 1	−6412.3663	H0 vs. H1	134.9926	*p <* 0.01	ω_0_ = 0.3149, ω_1_ = 1.0000
Foreground branch: ancestral of *Mandrillus*, *Cercocebus*, *Papio*, *Lophocebus*, *Theropithecus* and *Macaca*					
H1: ω_0,_ ω_1_	−6343.4866	M0 vs. H1	8.0522	*p <* 0.01	ω_0_ = 0.2419, ω_1_ = +∞
Foreground branch: ancestral of *Pan*, *Homo* and *Gorilla*					
H1: ω_0,_ ω_1_	−6345.0477	M0 vs. H1	4.9300	*p <* 0.05	ω_0_ = 0.2379, ω_1_ = 0.7772
H0: ω_0,_ ω_1_ = 1	−6345.1667	H0 vs. H1	0.2380	*p* > 0.05	ω_0_ = 0.2379, ω_1_ = 1.0000
***Site model***					
M1a	−6316.1817				p_0_ = 0.8312, p_1_ = 0.1688, ω_0_ = 0.0859, ω_1_ = 1.0000
M2a	−6310.5399	M1a vs. M2a	11.2836	*p <* 0.01	p_0_ = 0.8708, p_1_ = 0.1159, p_2_ = 0.0133, ω_0_ = 0.1137, ω_1_ = 1.0000, ω_2_ = 4.7335
M8a	−6316.2094				p_0_ = 0.8318( p_1_ = 0.1682), p = 9.4660, q = 99.0000, ω = 1.0000
M8	−6310.7199	M8a vs. M8	10.9790	*p <* 0.01	p_0_ = 0.9794( p_1_ = 0.0206), p = 0.4478, q = 1.7622, ω = 4.0147
***Branch-site model***					
Lineages of Cercopithecidae					
Null	−6316.1817				p_0_ = 0.8312, p_1_ = 1688 p_2a_ = 0.0000, p_2b_ = 0.0000 ω_0_ = 0.0859, ω_1_ = 1.0000 ω_2_ = 1.0000
Alternative	−6308.6453		15.0728	*p <* 0.01	p_0_ = 0.8384, p_1_ = 1583 p_2a_ = 0.0027, p_2b_ = 0.0005 ω_0_ = 0.0936, ω_1_ = 1.0000 ω_2_ = 13.4610

+∞ means infinite, and in this case *d*_*S*_ *=0* thus ω (*d*_*N*_/*d*_*S*_)= +∞.

### Positively selected sites on *TAS1R* genes within catarrhine primates

In site models, LRTs showed that the incorporate selections (i.e. M2a and M8) fitted significantly better than neutral models (i.e. M1a and M8a) for *TAS1R1* and *TAS1R2*, whereas no significant evidence of positive selection was found for *TAS1R3* (Tables 1, 2 and 3). In the M2a model, six and one sites for *TAS1R1* and *TAS1R2*, respectively, were under positive selection. Model M8 revealed six and 10 positively selected sites for *TAS1R1* and *TAS1R2*, respectively, identified by the BEB approach with posterior probabilities larger than 0.85 (see Additional file 6).

**Table 3:** **CODEML analyses of selective pattern on the** ***TAS1R3*** **in catarrhine primates**

**Models**	**lnL**	**Compared**	**2ΔLnL**	***P-value***	**Parameter estimates**
***Branch model***					
M0:one-ratio	−7676.8782				ω = 0.1342
M1:free-ratio	−7638.3982	M0 vs. M1	76.9600	*p* > 0.05	
***Site model***					
M1a	−7623.3051				p_0_ = 0.8913, p_1_ = 0.1087, ω_0_ = 0.0632, ω_1_ = 1.0000
M2a	−7623.3051	M1a vs. M2a	0.0000	*p* > 0.05	p_0_ = 0.8913, p_1_ = 0.0609, p_2_ = 0.0478, ω_0_ = 0.0632, ω_1_ = 1.000, ω_2_ = 1.0000
M8a	−7619.6648				p_0_ = 0.9461( p_1_ = 0.0539), p = 0.2391, q = 1.8178, ω = 1.0000
M8	−7619.7175	M8a vs. M8	0.1054	*p* > 0.05	p_0_ = 0.9678( p_1_ = 0.0322), p = 0.2634, q = 1.9053, ω = 1.1121
***Branch-site model***					
Lineages of *Macaca*					
Null	−7622.2362				p_0_ = 0.8389, p_1_ = 0.1004 p_2a_ = 0.0543, p_2b_ = 0.0065 ω_0_ = 0.0619, ω_1_ = 1.000, ω_2_ = 1.0000
Alternative	−7620.4248		3.6228	*p <* 0.05	p_0_ = 0.8877, p_1_ = 0.1058 p_2a_ = 0.0059, p_2b_ = 0.0007 ω_0_ = 0.0621, ω_1_ = 1.000, ω_2_ = 21.4208

**Figure 4:**
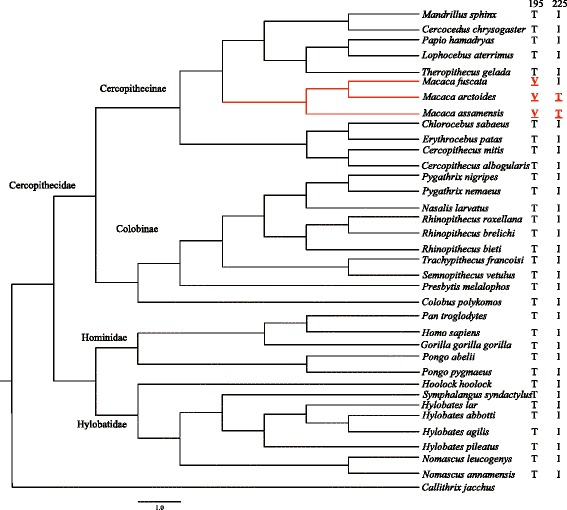
**Radical amino acid changes in selected sites across the catarrhine primates phylogeny of** ***TAS1R3.*** The input tree used for pressure analysis of *TAS1R3* is shown on the left. Positively selected sites detected by site models and branch-site models are shown on the right. Radical changes are shown in bold and underlined, and changes detected by branch-site models are marked in red additionally. *Callithrix jacchus* was used as outgroup, but not included in the CODEML analysis. Lineages under positive selection are marked in red.

The branch-site model was then used to test for positive selection in potential codons in lineages of separate groups of catarrhine primates, i.e., in Cercopithecidae, Cercopithecinae, Colobinae, Hylobatidae, and Hominidae (Tables 1, 2 and 3). The LRT results showed that Hylobatidae-specific lineages for *TAS1R2*, and Cercopithecidae-specific lineages for both *TAS1R1* and *TAS1R2* were subjected to strong positive selection. Further analysis indicated that lineages of Cercopithecinae and Colobinae for *TAS1R2* (Figure 2) and *Macaca*-specific lineages for *TAS1R3* were also under strong positive selection (Figure 4). In addition, 11 codons (*TAS1R1*: 391; *TAS1R2*: 8, 21, 175, 404, 411, 413, 510 and 733; *TAS1R3*: 195 and 225) were also identified by the BEB approach with posterior probabilities larger than 0.85 in branch-site analysis (see Additional file 6). Finally, most of these changes detected by both site models and branch-site models were identified to be critical (see Additional file 6).

### Prediction of amino acid sites of TAS1R1 and TAS1R2 responsible for umami and sweet taste molecules binding

**Figure 5:**
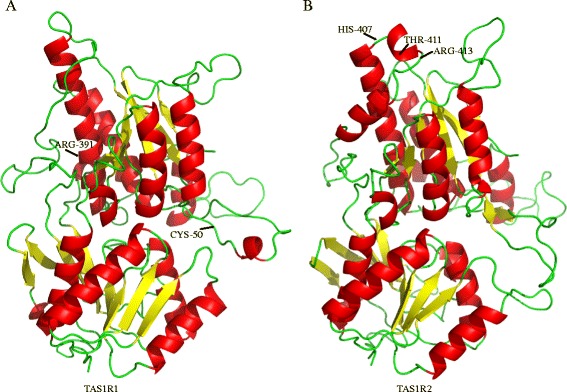
**Homology models of TAS1R1 and TAS1R2 VFTM domain. (A)** The model of TAS1R1 VFTM domain, and positive selection sites locating at binding regions are marked. **(B)** The model of TAS1R2 VFTM domain, and positive selection sites locating at binding regions are marked.

In order to examine whether positively selected sites detected in this study were responsible for binding of some umami and sweet taste molecules, we built homology models of TAS1R1 and TAS1R2 VFTM domain and docked with some sweet and umami taste molecules (Figure 5). Then, we got a series of docking results and found that most of these molecules bound with taste receptors at specific regions of TAS1R1 and TAS1R2. Finally, we mapped positive selection sites detected in our study onto models of TAS1R1 and TAS1R2 and found that sites 50 and 391 of TAS1R1 may interact with inosine monophosphate (IMP) and L-glutamate (see Additional file 7). Similarly, sites 342, 407, 411 and 413 of TAS1R2 were also found to be within binding domains of D-tryptophan, D-galactose, D-glucose, fructose, galactose and sucrose (see Additional file 8).

### Critical sites influencing the response of *TAS1R* genes to tastants

Previous functional expression data showed that mutations in two amino acid residues (S40T and I67S) of TAS1R2 were predicted to affect receptor response to aspartame and neotame, i.e., S40T abolished aspartame sensitivity and I67S reduced response to neotame slightly [31] (Table 4). Investigation of the amino acid residue 40 of TAS1R2 revealed a serine residue (S) in Hylobatidae and Hominidae, but a threonine residue (T) in Cercopithecidae. Furthermore, residue 67 is isoleucine (I) in most species, but methionine (M) in Colobinae (Table 4). Substitution at residue 733 (733A in Hylobatidae and Hominidae, 733 V in Cercopithecidae) of TAS1R3 was previously suggested to reduce its sensitivity to lactisole [32]. Furthermore, substitution R757C in human TAS1R3 seems to reduce responses to monosodium glutamate (MSG) and to increase binding with monopotassium glutamate (MPG) [13,16] (Table 4). This site shows histidine (H) in the genus *Pygathrix* and arginine (R) in other species (Table 4).

**Table 4:** **Functional TAS1R binding sites for sweet and umami tastants from previous studies and the mutation in catarrhine primates**

**Compounds**	**Interact receptors**	**Amino acid mutations**	**Results**	**Mutations in catarrhine monkeys**	**References**
Aspartame	hTAS1R2^1^	S40T	abolish aspartame sensitivity	S40T in Cercopithecidae	Liu et al. [31]
Neotame	hTAS1R2	I67S	reduce response to neotame sightly	I67M only in Colobinae	Liu et al. [31]
Lactisole	hTAS1R3	A733V	reduce sensitivity to lactisole	A733V in Cercopithecidae	Jiang et al. [32]
Monosodium glutamate (MSG)	hTAS1R3	R757C	reduce sensitivity to MSG	R757H in *Pygathrix*	Raliou et al. [16]
Monopotassium glutamate (MPG)	hTAS1R3	R757C	increase sensitivity to MPG	R757H in *Pygathrix*	Chen et al. [13]

^1^h denotes human.

## Discussion

The present study conducted the most comprehensive investigation to date of selection pressure on sweet and umami taste genes (*TAS1Rs*) in catarrhine primates to test whether specific adaptive evolution occurred during their diversification in association with particular plant diets. Our results support differentiated evolution and episodic relaxation of selective constraints on *TAS1R* in herbivorous/omnivorous catarrhine primates.

### Non-massive pseudogenization of *TAS1Rs* in catarrhine primates

As predicted above, no massive pseudogenization of *TAS1Rs* in catarrhine primates were founded in our data. However, disrupting mutations of *TAS1Rs* were revealed in *P. nemaeus*, *S. vetulus*, *T. francoisi* and *L. aterrimus*, which were supposed to influence species-specific functional differences in sweet and/or umami tastes (Figure 1). In the two species of *Pygathrix* (*P. nigripes* and *P. nemaeus*) tested in this study, one pseudogenized allele resulting in the truncation of the protein in VFTM was found in *TAS1R1* of *P. nemaeus*, which was otherwise intact in *P. nigripes*. Subsequent analysis of the *TAS1R1* gene in *P. cinerea* (data not shown) confirmed that this pseudogenized allele occurred only in *P. nemaeus*. Thus, considering their close phylogenetic relationships, the cause and biological influence of this one-allele pseudogenization of *TAS1R1* seems to be informative for a comparative analysis of *Pygathrix*. This pseudogenization of one *TAS1R1* allele in *P. nemaeus* likely weakens the biological function of this gene and decreases categories of discernible umami compounds. Suggestively, *P. nemaeus* shows the lowest diversity of recorded consumed plants compared to *P. cinerea* and *P. nigripes* [33].

Unlike the pseudogenized allele of *TAS1R1* in *P. nemaeus* resulting in truncation of the protein in VFTM, the two-nucleotide deletion of *TAS1R3* in *S. vetulus* and *T. francoisi* and the transition from C to T of *TAS1R3* in *L. aterrimus* occurred in the C-terminus of TAS1R3 and leaded to a premature stop codon in C-terminal intracellular domain (CID). Until now, mutations in VRTM, CRD, TMD of TAS1Rs have been reported to influence the sensitivity of TAS1Rs to a variety of substances [13,15,17,31]. However, the *d*_*N*_/*d*_*S*_ ratio is significantly lower than 1 for CID in rodent TAS2R genes, demonstrating the operation of purifying selection and a vital role of CID [34]. The experimental verification of the function of CID for TAS1Rs is lacking, and the influences of these mutations on the CID of TAS1R3 await further research.

Pseudogenization of *TAS1R* genes has been reported in various species with dietary specialization, such as the giant panda (*Ailuropoda melanoleuca*), vampire bats (*Desmodus* spp.) and whales (Cetacea) [21,23,24]. Although the Colobinae display a certain degree of dietary specialization and feeds mainly on leaves, no massive loss of *TAS1R* genes was found. Furthermore, the pseudogene of *TAS1R2* found in *P. nemaeus* was heterozygous. For Cercopithecidae, the premature stop codons occurring in CID of *TAS1R3* in *S. vetulus*, *T. francoisi* and *L. aterrimus* seemed not to disrupt the main structure of TAS1R3, as they only shortened the C-terminus. While, as endangered species, it is difficult to get living organisms for experimental analysis to examine whether these stop codons would terminate *TAS1R3* function in these species. But as the only species of catarrhine primates with amino acids length mutations in such sequence conservative *TAS1Rs*, formation mechanisms and biology function of these mutations are interesting and need further research. Moreover, neither pseudogene nor premature stop codon was found in Hylobatidae, which are typical frugivores. These results imply a functional role of *TAS1R* genes in most (or even all) species of catarrhine primates.

### Episodic relaxation of selective constraints and subsequent positive selection on *TAS1R2* in leaf-eating Colobinae

As specific leaf-eating primates, Colobinae attracted our primary attention in this study. The lineage-specific analysis showed that the ancestral *TAS1R2* of Colobinae (branch ACo in Figure 2) has undergone significantly different selection pressure and relaxation of selective constrains (ω_1_ = 0.5583, *p <*0.05) compared with other lineages (Table 1). The folivorous colobines mostly prefer leaves, and some unripe fruits with low simple and water-soluble sugars; microbes in the foregut of colobines can also degrade crude fibers of leaves and produce sugar, proteins and vitamins [35,36]. Additionally, colobines can recover nutrients by breaking down and digesting bacteria in a true stomach using various enzymes [37,38]. Lysozyme, one of those bacteriolytic enzymes, was documented to be under positive natural selection in a common ancestor of the foregut-fermenting colobine monkeys, which coincided with the establishment of leaf-eating and foregut-fermentation [39]. Therefore, sugar content in food was apparently not very important for ancestral colobines. Moreover, colobines are vulnerable to dietary change because the foregut microbes are not buffered by acidic defenses of the stomach [40]; the high simple sugar content of ripe and pulpy fruits will lower fore-stomach pH value, which can disrupt fermentation and induce bloating or acidosis, resulting in death [41-44]. As a consequence, the function or sensitivity of sweet taste may have been less necessary for the common ancestor of Colobinae, and selection pressure acting on functional sweet taste gene underwent a certain degree of relaxation. Consistent with this assumption, we indeed detected such a relaxation in the ancestral Colobinae *TAS1R2* gene. In addition, we also found fourteen specific amino acid substitutions in the ancestral *TAS1R2* gene of Colobinae (I67M, E118D, D119N, D225N, P348S, E423K, I436L, S547R, M616V, A635V, A649T, T686M, M697I and V741L) through alignment with the ancestral *TAS1R2* of Cercopithecidae and Cercopithecinae. Interestingly, except for D225N and A649T, all other amino acid substitutions were unique and fixed in colobine species, suggesting that these amino acid changes reflect a necessary adaption to their specific diet.

Interestingly, branch-site analysis indicated that *TAS1R2* was subjected to strong positive natural selection (ω_2_ = 3.5818, *p <*0.05) in lineages of extant colobines, and also suggested substitutions I8F and I510V (posterior probability ≥0.850) as candidate sites for selection (Table 1). It seems to be a paradox that *TAS1R2* would have undergone positive selection in extant leaf-eating colobines, which prefer low simple and water-soluble sugars. However, they actually prefer young leaves and only few fruits (especially ripe fruits), which contain certain proportion of proteins and sugars and are also preferred by other catarrhine primates [43-46]. It has been reported that *P. cinerea* eat 49.5% young leaves, 21.9% ripe fruits, 19.1% unripe fruits and only 9.3% mature leaves; the nutritional component in selected food of *P. cinerea* consists of protein at 11.4%, dry matter, lipids at 2.6%, minerals at 5.0%, sugars at 4.9%, starch at 12.8%, and neutral detergent fibers at 40.8% [33]. The sense of sweetness is associated with sugars, some amino acids, a few proteins, and glucosides [47-49]. Perhaps the sensitivity of TAS1R2 for sweeteners in foods is essential for extant colobines. After the establishment of leaf-eating and foregut-fermenting, extant colobines would have undergone further selection to detect new nutrient substance through TAS1R2.

### Positive selection of *TAS1R1* in Cercopithecidae

The branch-site model revealed that the *TAS1R1* lineage of all Cercopithecidae underwent positive selection, and that substitutions R391C and R391Q are candidate sites for selection. Primates can sense the presence of protein through the taste of umami, which is elicited by glutamate and glutamic acid [50,51]. Commonly incorporated in leaf protein, glutamic acid is also reported from nectar and figs [52,53]. Besides dietary differences between Cercopithecinae and Colobinae, both groups also require proteins in plant foods (and animal foods). According to Yeager & Kool [54], colobines select foods of high nutritional value, and young leaves are preferred over mature leaves. Young leaves contain more protein and less fiber than mature leaves [44]. Furthermore, it was reported that *Rhinopithecus bieti* tended to choose leaves with high content of crude protein, as well as insects and even birds [55]. Thus, it is assumed that the taste of umami is important not only for Cercopithecidae but also for Colobinae.

### Positive selection of *TAS1R2* in Cercopithecinae

Branch-site analysis showed that *TAS1R2* in Cercopithecinae is subject to strong positive natural selection (ω_2_ = 5.8766, *p <*0.01), and the substitutions T411A, R413I and R413K (posterior probability ≥0.850) were indicated as candidate sites for selection (Table 1). However, compared to colobines, for which *TAS1R2* is also under positive natural selection, the candidate sites of positive natural selection in Cercopithecinae are different. The functions of the different positively selected sites of *TAS1R2* in Cercopithecinae and Colobinae await further research, and perhaps these mutations have changed sensitivity for certain substances. It might reflect the common importance of sweetness for Cercopithecinae and extant colobines, along with variation in types and proportions of different kind of sweet substances.

### Positive selection of *TAS1R3* in Macaques

*TAS1R3* showed positive natural selection in the genus *Macaca*. As described above, *TAS1R3* is the most conserved member among the *TAS1R* gene family due to its basic function in both sweet and umami taste receptors. Therefore, as the only group of lineages in which *TAS1R3* was detected to be under strong positive natural selection, the genus *Macaca* was suggested to possess special sweet and umami tastes and food perceptions different to that of other taxa. The genus *Macaca* represents one of the most successful primate radiations. While most cercopithecines are confined in Africa, except for the Barbary macaque of northern Africa [56], macaques are widely distributed in Asia, north to Japan and west to Afghanistan, which represented the only Asian linage in Cercopithecinae [57-59]. According to fossil data, the earliest macaque originated in northeast Africa around 7 million years ago [60,61], and spread through most of Eurasia [62]. Now, they are found in a wide range of habitats, from evergreen forests to grassland and even areas modified by humans, from tropical forests to temperate ecosystems, and from continents to deep-water islands [57]. This range is likely reflected in dietary diversity as well.

### Positive selection of *TAS1R2* in Hylobatidae

Branch-site analysis showed that lineages of Hylobatidae were under significant positive natural selection for *TAS1R2* (ω_2_ = 11.8832, *p <*0.01), indicating adaptive evolution of sweet taste in gibbons. As well-documented frugivores, fruits (especially ripe fruits), account for more than 50% of gibbons’ diet, except for the more folivorous siamang (*Symphalangus syndactylus*) [63-66]. It is commonly agreed that food choices of primates are correlated with the nutritional and toxic contents [65,67,68]. Compared with other parts of plant, fruits typically have the highest content of soluble carbohydrates, i.e., sweet tasting compounds [69,70]. As typical frugivorous primates, gibbons seem to intake food with more soluble sweet taste contents than other primates. For example, white-handed gibbons were reported to include a high proportion of carbohydrate-rich fruits in their natural diet [71] and showed clear preferences toward ripe fruits, which typically have the highest content of soluble carbohydrates [69,70]. Furthermore, a food preference test of captive white-handed gibbons with ten types of fruits revealed a highly significant positive correlation between the food preference ranking and total carbohydrates [67]. Accordingly, total carbohydrate content in foods might be an important determinant of food choice for gibbons [72-77], which may contribute to specific adaptive evolution of *TAS1R2*. Four amino acid substitutions (M132S, V175A, G404D and G404N) were detected as candidates for natural selection, which may have influenced the sensitivity of TAS1R2 in gibbons and adapted to their special frugivorous diet.

### Why is there no positive selection of *TAS1R* genes in Hominidae?

Interestingly, no signs of positive selection were detected in Hominidae. This could be explained by external and internal reasons. Hominid species are able to acquire, process and consume a wide variety of foods such as fruits, assorted types of vegetation, bark, seeds, insects and meat. They are able to change the composition of diet due to local ecological conditions as they have the ability of tool usage to acquire and consume foods [78]. Thus, sensitivity to sweet and umami taste might not play an overwhelming role in their feeding ecology. From another aspect, sensitivity of taste is also correlated with the body size of primates [47]. A large area of the lingual mucosa in large animals may increase taste performance. This means that the larger the species, the better their taste acuity [47]. As the largest species among the primates, the sensitivity of umami and sweet taste in Hominidae might be sufficiently sensitive for foraging.

Additionally, an episodic relaxation of selective constraints (ω_1_ = 0.7772, *p <* 0.05) was also found in *TAS1R1* for the common ancestor of human, chimpanzee, bonobo and gorilla (Table 2, Figure 3). In contrast, diet and food availability for orang-utans exert a much more restrictive influence. These large, arboreal great apes rely predominantly on fruit, such as figs (*Ficus* spp.) and durian (*Durio* spp.) This difference in diet might explain the variation in the evolutionary history of *TAS1R1* between orang-utans and other great apes (humans included).

### Functional sites concentrated on extra-cellular region of taste receptors and phylogenetically scattered

Nearly all of the positive selected sites (92.3%, 24 out of 26 positive selected sites, see Additional file 2) were localized in the VFTM and CRD domains (extra-cellular region) of TAS1R, which are responsible for binding of small molecules [79]. In order to examine potential influence of these positively selected mutations, we conducted the commonly used molecular modeling and docking methods to detect binding of some sweet and umami taste molecular with TAS1Rs receptors. Interestingly, we located some positive selection sites at binding regions of several sweet and umami taste molecules based on the molecular modelling and docking results. Sites 50 and 391 of TAS1R1, and sites 342, 407, 411 and 413 of TAS1R2 were identified to interact with several taste molecules, such as D-tryptophan, IMP, L-glutamate, fructose and sucrose (see Additional files 7, 8). It is remarkable that these amino acid sites were coincidently revealed to be positively selected sites. All of these results offer us potential active regions and important amino acid sites which are likely to influence the binding of several tastants, which also implies that the extra-cellular regions play a vital role in adaptive evolution. Certainly, comparing functional expression experiments, molecular modeling and docking have certain limitations. However, as the wildly used molecular structure analysis methods, molecular modeling and docking can predicate some potential binding domains and binding sites between receptors and ligands, theoretically. In our study, as the complementary analysis to predicate potential influence on binding with ligands of positively selected sites detected in our studies, the modeling and docking studies were suggested to be necessary and critical.

Remarkably, most of these radical changes in amino acids occurred in a few sub-terminal and terminal lineages across the phylogeny of catarrhine primates, without obvious phylogeny-related occurrence (Figures 2, 3 and 4). In other words, relatives belonging to the same phylogenetic group (family or genus) usually did not exhibit uniform amino acids in a single radical change site, and even several of these radical changes occurred irregularly in a single or a few distantly related species. This suggests specific adaptive evolution of *TAS1R* genes in catarrhine primates, resulting from habitat change and complexity of plant–based diets.

### Potential variation in tasting of aspartame, neotame, lactisole, monosodium glutamate and monopotassium glutamate among catarrhine primates

Although a potential functional change induced by these positively selected sites was not substantiated in this study, we referred to multiple mutation/substitution sites of *TAS1R* identified by previous functional expression studies in other species, and some of them also occurred in the sequences described here (Table 4). For example, the site substitution S40T of TAS1R2 was shown to abolish the receptor response to aspartame [31], suggesting that Cercopithecidae species showing this substitution might have lost the taste of aspartame (Table 4). However, other studies suggested that aspartame is perceived as sweet by humans, apes and some species of Cercopithecidae [20,80]. Variable perception strength to aspartame between Cercopithecidae and other species of catarrhine primates was suggested to be the best explanation for this ‘contradictory’ situation. We hypothesized that this amino acid substitution did not really abolish response of Cercopithecidae to aspartame, but rather led to differences in sensitivity of aspartame perception between Cercopithecidae and other species. Further functional expression studies about the influence of S40T on sensitivity of TAS1R2 in catarrhine primates should be launched in future. Additionally, the A733V substitution in TAS1R3 was one of the important changes detected for diminished receptor sensitivity to lactiosole, according to research conducted in humans and rodents [32]. The same substitution (A733V) occurred in Cercopithecidae, where A was found in Hylobatidae and Hominidae. It is reasonable to hypothesize that the A733V substitution in TAS1R3 of Cercopithecidae potentially led to more diminished sensitivity to lactisole than in Hylobatidae and Hominidae.

The substitution I67S of TAS1R2 in humans was confirmed to slightly reduce the response to neotame. Furthermore, the substitution R757C of TAS1R3 in humans reduces sensitivity to monosodium glutamate (MSG) and increases sensitivity to monopotassium glutamate. Substitutions at the same sites of TAS1R2 and TAS1R3 but not the same amino acid changes were found in Colobinae (Table 4) and their potential influence on TAS1R2 and TAS1R3 in Colobinae is expected to be evaluated during future studies.

Though some of these tastants were artificial compounds and have less of relationship with adaption of the sweet and umami tastes, these work supplied examples which reflect the mutation-induced sensitivity variation for certain compounds no matter natural or artificial. It highlights the importance of a number of sites and domains for ligands binding. Similar work can be implemented in research on catarrhine primates incorporating more natural compounds to examine the potential influence of positive selection sites detected in our study.

## Conclusions

Unlike animal food, the number and diversity of nutritional compounds of leaves and fruit varies with plant species, location, position on the tree, stage of development, and even time of day [27,81-84]. Therefore, it is reasonable to assume that catarrhine primates require more sensitive and specific sweet and umami tastes when compared to carnivorous animals. Our results demonstrate family-, subfamily-, genus- and even species-specific adaptive evolution of *TAS1R* genes, and suggest pervasively differentiated evolution of sweet and umami tastes during the divergence of catarrhine primates. Episodic relaxation of selective constraints and pseudogenization were also found, both of which might be accompanied by and even contribute to dietary transition. Remarkably, positive selected sites were concentrated on extra-cellular region of taste receptors, which indicated that extra-cellular region of TAS1Rs play a vital role in adaption for variations in sweet and umami taste molecules in different diets.

The relationships among gustatory sense, food intake and foraging efficiency are complicated [85]. Both physical and chemical properties of food play important roles in food choice in primates [86]. Thus, the influence of both external and internal factors on taste receptor gene evolution is complex. Given such multiple factors, the impact of any particular factor is likely to be quantitative rather than qualitative, and a small number of counterexamples do not automatically refute any potential. Given these complications, to avoid spurious results, it is imperative to examine a large and diverse group of species when testing the potential impact of any given ecological factor on taste receptor gene evolution [87]. Our study provides new insights into the evolutionary history of taste genes in primates, as well as a database of mutations/substitutions in taste genes which will facilitate future work assessing the ecological correlates between taste sensitivity and food choice in primates.

## Methods

### Ethical approval

Blood samples were taken during routine health checks by experienced veterinarians and not specifically for this study. Fresh tissue samples were taken from deceased animals. All research complied with protocols approved by the Animal Welfare Body of the DPZ in Germany and the Wildlife Conservation Association in China, and adhered to the legal requirements of the countries, in which research was conducted. The study was carried out in compliance with the principles of the American Society of Primatologists for the ethical treatment of non-human primates (https://www.asp.org/society/resolutions/EthicalTreatmentOfNonHumanPrimates.cfm). No animals were sacrificed for this study.

### Polymerase chain reaction and DNA sequencing

Based on an alignment of currently available *TAS1R* sequences of human, chimpanzee, and macaque, we designed a set of primer pair to amplify *TAS1R* sequences from DNA samples of 30 species in catarrhine monkeys (see Additional file 1). The polymerase chain reaction (PCR) mixtures (50 ul) contained 5 ul (50 ng/ul) genomic DNA, 25 ul of 2 × buffer, 7.5 ul (50 mM) MgCl_2_, 5ul (10 mM) of each primer, and 1 U Taq DNA polymerase (Takara). PCRs were performed in a DNA Engine Dyad Cycler (BioRad) under the following condition: 5 min of initial denaturation, 30 cycles of denaturation at 94°C for 30 s, annealing at 55°C for 30 s, extension at 72°C for 60 s; and a final extension at 72°C for 5 min. PCR products were examined on agarose gels and subsequently cloned in the PMD18-T (Promega) cloning vector. Positive clones were sequenced on an ABI 3130 xl DNA Sequencer. Three to five clones of each PCR product were sequenced from both directions to validate the results. All intact *TAS1R* sequences of Hominidae were downloaded from GeneBank (see Additional file 1). Additionally, we also aligned our sequences with available primate genomes from GeneBank and the genome assembly of snub-nosed monkeys (unpublished), confirming that *TAS1R* are single-copy genes in genomes of catarrhine primates. The nucleotide and deduced amino acid sequences of each gene were aligned with CLUSTALX 1.81 [88] and modified with Bio-Edit 7.0.4 [89].

### Selective pressure detection

A powerful approach to detecting molecular evolution by positive selection derives from comparison of the relative rates of synonymous (*d*_*S*_) and non-synonymous substitutions (*d*_*N*_/) [90-92]. The rate ratio ω (*d*_*N*_/*d*_*S*_) is a measure of selective pressure, where ω =1, ω <1 and ω >1 correspond to neutral evolution, purifying and positive selection [93]. The ω ratio was estimated using a codon-based maximum-likelihood method implemented in CODEML program of the PAML v. 4.4 package [94]. A well-accepted phylogeny of primates was used as the input tree in all analyses [95]. Because only one or two intact *TAS1R* were available in some hominoids, input trees used in the analysis of *TAS1R1*, *TAS1R2*, and *TAS1R3* were slightly different within lineages of Hominidae (Figures 2, 3 and 4).

A combination of branch, site and branch-site models was used to analyze the selection of *TAS1R* in catarrhine primates. Firstly, to test whether the ω ratio of each gene was different among lineages, the ‘free-ratios’ model (M1), which assumes an independent ω ratio for each branch, was compared with the ‘one-ratio’ model (M0), which assumes the same ω ratio for all branches [93]. Subsequently, site models in which ω can vary among sites were implemented to identify candidates of positively selected sites in the *TAS1R* genes [96,97]. Therefore, two pairs of site models were tested: M1a (nearly neutral) versus M2a (positive selection), and M8a (nearly neutral; beta distribution) versus M8 (positive selection; beta distribution).

Additionally, positive selection often operates episodically on a few amino acid sites in a small number of lineages in a phylogenetic tree [98]. Therefore, in the case of branch-site model, modified branch-site model A was performed for each gene in the lineages of Cercopithecidae, Cercopithecinae, Colobinae, Hylobatidae and Hominidae, separately. Finally, two-ratio model assuming the branches of interest having a *d*_N_/*d*_S_ (ω_1_) different from the background ratio (ω_0_) was used for lineages with 0.50 < ω <1 (detecting by free ratio model) to identify potential functional relaxation [99,100]. Ancestral *TAS1R* sequences were inferred based on empirical Bayesian methods implemented in the CODEML program of the PAML package.

The likelihood ratio test (LRT), which calculates twice the log-likelihood (2ΔLnL) of the difference following a chi-square distribution, was used to evaluate the significance of differences between each pair of models. All of the pseudogenes were removed in the PAML-based analysis. To evaluate the probabilities of positively selected sites on *TAS1R* sequenced in this research, the Bayes empirical Bayes (BEB) analysis was used to calculate posterior probabilities of positively selected sites implemented in the CODEML program of PAML. Based on BEB analysis, sites with a posterior probability >0.85 were considered as candidates for selection. Finally, according to charge, polarity and volume of amino acids, positive selection sites detected by site models and branch-site models were used to estimate amino acids change patterns (conservative or radical substitutions) along the evolution lineages of primates [98].

### Molecular modelling and docking

Homology models of TAS1R1 and TAS1R2 VFTM (amino acids 29–496 and 24–494, respectively) were built with the Modeller9.11 [101,102] and EasyModeller4.0 [103] using the mGluR1-VFTM crystal structure (PDB entry: 2U4E) [104] as the template. Then, automatic molecular docking programs, Autodock Vina1.1.2 [105] and MGLTools1.5.6 [106,107] were used for the docking studies with some sweet and umami taste molecules (umami molecules: glycine, IMP, L-alanine, L-glutamate; sweet molecules: D-tryptophan, fructose, D-fructose, galactose, D-galactose, D-glucose and, sucrose, neotame and aspartame), and these results were viewed modified by PyMol (The PyMOL Molecular Graphics System, Version 1.7 Schrödinger, LLC).

## Additional files

Additional file 1:
**Information about the species and genes present in this study.**
Additional file 2:
**Summary of mutation sites and positive selected sites in TAS1Rs proteins.**
Additional file 3:
**Alignment of TAS1R1 amino acid sequences of 35 catarrhine primates. **Variant residues are marked with red. Additional file 4:
**Alignment of TAS1R2 amino acid sequences of 35 catarrhine primates.** Variant residues are marked with red. Additional file 5:
**Alignment of TAS1R3 amino acid sequences of 35 catarrhine primates.** Variant residues are marked with red. Additional file 6:
**Candidate amino acid sites under positive selection identified in Tas1rs using the site models and branch-site models.**
Additional file 7:
**Molecular docking results of TAS1R1.** Positive selection sites detected in our research are marked with red. Additional file 8:
**Molecular docking results of TAS1R2.** Positive selection sites detected in our research are marked with red.

## References

[1] Bachmanov AA, Beauchamp GK (2007). Taste receptor genes. Annu Rev Nutr.

[2] Lindemann B (1996). Taste reception. Physiol Rev.

[3] Li XD, Staszewski, Xu H, Durick K, Zoller M, Adler E, Affiliations A (2002). Human receptors for sweet and umami taste. Proc Natl Acad Sci U S A.

[4] Nelson G, Hoon MA, Chandrashekar J, Zhang YF, Nicholas JP, Ryba, Zuker CS (2001). Mammalian sweet taste receptors. Cell.

[5] Shi P, Zhang J (2006). Contrasting modes of evolution between vertebrate sweet/umami receptor genes and bitter receptor genes. Mol Biol Evol.

[6] Pin JP, Galvez T, Prezeau L (2003). Evolution, structure, and activation mechanism of family 3/C G-protein-coupled receptors. Pharmacol Ther.

[7] Jingami H, Nakanishi S, Morikawa K (2003). Structure of the metabotropic glutamate receptor. Curr Opin Neurobiol.

[8] Jiang P, Ji Q, Liu Z, Snyder LA, Benard LM, Margolskee RF, Max M (2004). The cysteine-rich region of T1R3 determines responses to intensely sweet proteins. J Biol Chem.

[9] Jiang P, Cui M, Zhao B, Snyder LA, Benard LM, Osman R, Max M, Margolskee RF (2005). Identification of the cyclamate interaction site within the transmembrane domain of the human sweet taste receptor subunit T1R3. J Biol Chem.

[10] Winnig M, Bufe B, Kratochwil NA, Slack JP, Meyerhof W (2007). The binding site for neohesperidin dihydrochalcone at the human sweet taste receptor. BMC Struct Biol.

[11] Xu H, Staszewski L, Tang H, Adler E, Zoller M, Li X (2004). Different functional roles of T1R subunits in the heteromeric taste receptors. Proc Natl Acad Sci U S A.

[12] Zhang F, Klebansky B, Fine RM, Liu H, Xu H, Servant G, Zoller M, Tachdjian C, Li X (2010). Molecular mechanism of the sweet taste enhancers. Proc Natl Acad Sci U S A.

[13] Chen QY, Alarcon S, Tharp A, Ahmed OM, Estrella NL, Greene TA, Rucker J, Breslin PAS (2009). Perceptual variation in umami taste and polymorphisms in *TAS1R* taste receptor genes. Am J Clin Nutr.

[14] Cui M, Jiang P, Maillet E, Max M, Margolskee RF, Osman R (2006). The heterodimeric sweet taste receptor has multiple potential ligand binding sites. Curr Pharm Des.

[15] Jiang P, Cui M, Ji Q, Snyder L, Liu Z, Benard L, Margolskee RF, Osman R, Max M (2005). Molecular mechanisms of sweet receptor function. Chem Senses.

[16] Raliou M, Grauso M, Hoffmann B, Schlegel-Le-Poupon C, Débat H, Belloir C, Wiencis A, Sigoillot M, Bano SP, Trotier D, Pernollet JC, Montmayeur JP, Faurion A, Briand L (2011). Human genetic polymorphisms in T1R1 and T1R3 taste receptor subunits affect their function. Chem Senses.

[17] Assadi-Porter FM, Tonelli M, Maillet EL, Markley JL, Max M (2010). Interactions between the human sweet-sensing T1R2-T1R3 receptor and sweeteners detected by saturation transfer difference NMR spectroscopy. Biochim Biophys Acta.

[18] Nie Y, Vigues S, Hobbs JR, Conn GL, Munger SD (2005). Distinct contributions of T1R2 and T1R3 taste receptor subunits to the detection of sweet stimuli. Curr Biol.

[19] Winnig M, Bufe B, Meyerhof W (2005). Valine 738 and lysine 735 in the fifth transmembrane domain of rTas1r3 mediate insensitivity towards lactisole of the rat sweet taste receptor. BMC Neurosci.

[20] Li X, Bachmanov AA, Maehashi K, Li W, Lim R, Brand JG, Beauchamp GK, Reed DR, Thai C, Floriano BW (2011). Sweet taste receptor gene variation and aspartame taste in primates and other species. Chem Senses.

[21] Zhao H, Zhou Y, Pinto M, Dominique PC, Gonzalez JG, Zhang S, Zhang J (2010). Evolution of the sweet taste receptor gene Tas1r2 in bats. Mol Biol Evol.

[22] Li X, Li WH, Cao J, Maehashi K, Huang LQ, Bachmanov AA, Reed DR, Beauchamp GK, Brand JG (2005). Pseudogenization of a sweet-receptor gene accounts for cats’ in difference toward sugar. PLoS Genet.

[23] Zhao H, Yang JR, Xu H, Zhang J (2010). Pseudogenization of the umami taste receptor gene Tas1r1 in the giant panda coincided with its dietary switch to bamboo. Mol Biol Evol.

[24] Zhao H, Zhang J (2012). Mismatches between feeding ecology and taste receptor evolution: An inconvenient truth. Proc Natl Acad Sci U S A.

[25] Jiang P, Josue J, Li X, Glaser D, Li W, Brand JG, Margolskee RF, Reed DR, Beauchamp GK (2012). Major taste loss in carnivorous mammals. Proc Natl Acad Sci U S A.

[26] Bachmanov AA, Bosak NP, Lin C, Matsumoto I, Ohmoto M, Reed DR, Nelson TM (2014). Genetics of taste receptors. Curr Pharm Des.

[27] Chapman CA, Chapman LJ, Rode K, Hauck EM, Mcdowell LR (2003). Variation in the nutritional value of primate foods: among trees, time periods, and areas. Int J Primatol.

[28] Pereira PM, Vicente AF (2013). Meat nutritional composition and nutritive role in the human diet. Meat Sci.

[29] Struhsaker TT (1995). Colobine monkeys: their ecology, behaviour and evolution. Int J Primatol.

[30] Bauchop T (1971). Stomach microbiology of primates. Annu Rev Microbiol.

[31] Liu B, Ha M, Meng XY, Kaur T, Khaleduzzaman M, Zhang Z, Jiang P, Li X, Cui M (2011). Molecular mechanism of species-dependent sweet taste toward artificial sweeteners. J Neurosci.

[32] Jiang P, Cui M, Zhao B, Liu Z, Snyder LA, Benard LMJ, Osman R, Margolskee RF, Max M (2005). Mechanisms of signal transduction: lactisole interacts with the transmembrane domain of human T1R3 to inhibit sweet taste. J Biol Chem.

[33] Tinh NT, Long HT, Tuan BV, Vy TH, Tam NA (2012). The feeding behavior and phytochemical food content of grey-shanked douc langurs (*Pygathrix cinerea*) at Kon Ka Kinh National Park, Vietnam. Vietnamese J Primatol.

[34] Wang XX, Thomas SD, Zhang JZ (2004). Relaxation of selective constraint and loss of function in the evolution of human bitter taste receptor genes. Hum Mol Genet.

[35] Blackburn TH, Dougherty RW (1965). Nitrogen metabolism in the rumen. Physiology of Digestion in the Ruminant.

[36] Hungate RE, Cole CF (1967). Ruminal fermentation. Handbook of Physiology.

[37] Barnard EA (1969). Biological function of pancreatic ribonuclease. Nature.

[38] Beintema JJ (1990). The primary structure of langur (*Presbytis entellus*) pancreatic ribonuclease: adaptive features in digestive enzymes in mammals. Mol Biol Evol.

[39] Messier W, Stewart CB (1997). Episodic adaptive evolution of primate lysozymes. Nature.

[40] Kool KM (1992). Food selection by the silver leaf monkey, *Trachypithecus auratus sondaicus,* in relation to plant chemistry. Oecologia.

[41] Collins L, Roberts M, Montgomery GG (1978). Arboreal folivores in captivity-maintenance of a delicate minority. The Ecology of Arboreal Folivores.

[42] Danish L, Chapman CA, Hall MB, Rode KD, Worman CO, Hohmann G, Robbins MM, Boesch C (2006). The role of sugar in diet selection in red tail and red colobus monkeys. Feeding Ecology in Apes and Other Primates.

[43] Davies G, Oates JF (1994). Colobine Monkeys: Their Ecology, Behaviour and Evolution.

[44] Milton K (1998). Physiological ecology of howlers (*Alouatta*): energetic and digestive considerations and comparison with the Colobinae. Int J Primatol.

[45] Dominy NJ, Lucas PW, Osorio D, Yamshita N (2001). The sensory ecology of primate food perception. Evol Anthropol.

[46] Workman C (2010). The foraging ecology of the Delacour’s langur (*Trachypithecus delacouri*) in Van Long Nature Reserve, Vietnam. PhD Dissertation.

[47] Hladik CM, Simmen B (1997). Taste perception and feeding behavior in nonhuman primates and human populations. Evol Anthropol.

[48] Rui-Lin N, Tanaka T, Zhou J, Tanaka O (1982). Phlorizin and trilobatin, sweet dihydrochalcone-glucosides from leaves of *Lithocarpus litseifolius* (Hance) Rehd. (Fagaceae). Agric Biol Chem.

[49] Schiffman S, Van der Starre H (1980). Magnitude estimation of amino acids for young and elderly subjects. Olfaction and Taste VII.

[50] Chaudhari N, Landin AM, Roper SD (2000). A metabotropic glutamate receptor variant functions as a taste receptor. Nat Neurosci.

[51] Rolls ET (2000). The representation of umami taste in the taste cortex. J Nutr.

[52] Simmen B, Sabatier D (1996). Diets of some French Guianan primates: food composition and food choices. Int J Primatol.

[53] Wendeln MC, Runkle JR, Kalko EKV (2000). Nutritional values of 14 fig species and bat feeding preferences in Panama. Biotropica.

[54] Yeager CP, Kool K, Whitehead J (2000). The behavioural ecology of Asian colobines. Catarrhine Monkeys.

[55] Ren BP, Li DY, Liu ZJ, Li BG, Wei FW, Li M (2010). First evidence of prey capture and meat eating by wild Yunnan snub-nosed monkeys *Rhinopithecus bieti* in Yunnan, China. Curr Zool.

[56] Fooden J (1982). Ecogeographic segregation of macaque species. Primates.

[57] Abegg C, Thierry B (2002). Macaque evolution and dispersal in insular south-east Asia. Biol J Linn Soc.

[58] Tosi AJ, Morales JM, Melnick DJ (2003). Paternal, maternal, and biparental molecular markers provide unique windows onto the evolutionary history of macaque monkeys. Evolution.

[59] Hassel-Finnegan H, Borries C, Zhao Q, Phiapalath P, Koenig A (2013). Southeast Asian primate communities: the effects of ecology and Pleistocene refuges on species richness. Integr Zool.

[60] Delson E, Szalay FS (1975). Evolutionary history of the Cercopithecidae. Approaches to Primate Paleobiology. Contributions to Primatology.

[61] Delson E, Lindburg DG (1980). Fossil macaques, phyletic relationships and a scenario of deployment. The Macaques: Studies in Ecology, Behavior, and Evolution.

[62] Delson E (1996). The oldest monkeys in Asia. International Symposium: Evolution of Asian Primates.

[63] Fan PF, Fei HL, Scott MB, Zhang W, Ma CY (2011). Habitat and food choice of the critically endangered cao vit gibbon (*Nomascus nasutus*) in China: implications for conservation. Biol Conserv.

[64] Fan PF, Ai HS, Fei HL, Zhang D, Yuan SD (2013). Seasonal variation of diet and time budget of Eastern hoolock gibbons (*Hoolock leuconedys*) living in a northern montane forest. Primates.

[65] Jildmalm R, Amundin M, Laska M (2008). Food preferences and nutrient composition in captive white-handed gibbons, *Hylobates lar*. Int J Primatol.

[66] Ni QY, Huang B, Liang ZL, Wang XW, Jiang XL (2014). Dietary variability in the Western Black Crested Gibbon (*Nomascus concolor*) inhabiting an isolated and disturbed forest fragment in southern Yunnan, China. Am J Primatol.

[67] Leighton M (1993). Modeling dietary selectivity by Bornean orangutans: evidence for integration of multiple criteria in fruit selection. Int J Primatol.

[68] Bollard EG, Hulme AC (1970). The physiology and nutrition of developing fruits. The Biochemistry of Fruits and Their Products.

[69] Stevenson PR (2003). Fruit choice by woolly monkeys in Tinigua National Park, Colombia. Int J Primatol.

[70] Simmen B, Hladik A, Ramasiarisoa PL, Iaconelli S, Hladik CM, Rakotosamimanana H (1999). Taste discrimination in lemurs and other primates, and the relationships to distribution of the plant allelochemicals in different habitats of Madagascar. New Directions in Lemur Studies.

[71] Chivers DJ, Preuschoft H, Chivers DJ, Brockelman WY, Creel N (1984). Feeding and ranging in gibbons: a summary. The Lesser Apes.

[72] Bartlett TQ, Campbell CJ, Fuentes A, Mackinnon KC, Panger M, Bearder SK (2007). The hylobatidae, small apes of Asia. Primates in Perspective.

[73] Carpenter CR, Dorcus RM (1940). A field study in Siam of the behavior and social relations of the gibbon (*Hylobates lar*). Comparative Psychology Monographs.

[74] Jolly A, Jolly A (1985). Food and feeding. The Evolution of Primate Behavior.

[75] Raemaekers J (1978). Changes through the day in the food choice of wild gibbons. Folia Primatol.

[76] Richard AF, Richard AF (1985). Primate diets: patterns and principles. Primates in Nature.

[77] Ungar PS (1995). Fruit preferences of four sympatric primate species at Ketambe, Northern Sumatra, Indonesia. Int J Primatol.

[78] Kleiman DG, Geist V, McDade MC (2004). Grzimek’s Animal Life Encyclopedia.

[79] Temussi PA (2009). Sweet, bitter and umami receptors: a complex relationship. Cell.

[80] Hellekant G, Danilova V, Ninomiya Y (1997). Primate sense of taste: behavioral and single chorda tympani and glossopharyngeal nerve fiber recordings in the rhesus monkey, *Macaca mulatta*. J Neurophysiol.

[81] Fernandez-Escobar R, Moreno R, Garcia-Creus M (1999). Seasonal changes of mineral nutrients in olive leaves during the alternate-bearing cycle. Sci Hort.

[82] Klages K, Donnison H, Wunsche J, Boldingh H (2001). Diurnal changes in non-structural carbohydrates in leaves, phloem exudate and fruit in ‘Braeburn’ apple. Aust J Plant Physiol.

[83] Marquis RJ, Newell EA, Villegas AC (1997). Non-structural carbohydrate accumulation and use in an understorey rain-forest shrub and relevance for the impact of leaf herbivory. Funct Ecol.

[84] Woodwell GM (1974). Variation in the nutrient content of leaves of *Quercus alba*, *Quercus coccinea*, and *Pinus rigida* in the Brookhaven forest from bud-break to abscission. Am J of Bot.

[85] Xiang Z, Liang W, Nie S, Li M (2013). Short notes on extractive foraging behavior in gray snub-nosed monkeys. Integr Zool.

[86] Blaine KP, Lambert JE (2012). Digestive retention times for Allen’s swamp monkey and L’Hoest’s monkey: data with implications for the evolution of cercopithecine digestive strategy. Integr Zool.

[87] Li DY, Zhang JZ (2014). Diet shapes the evolution of the vertebrate bitter taste receptor gene repertoire. Mol Biol Evol.

[88] Thompson JD, Gibson TJ, Plewniak F, Jeanmougin F, Higgins DG (1997). The CLUSTAL_X windows interface: flexible strategies for multiple sequence alignment aided by quality analysis tools. Nucleic Acids Res.

[89] Hall TA (1999). BioEdit: a user-friendly biological sequence alignment editor and analysis program for windows 95/98/NT. Nucleic Acids Symp Ser.

[90] Kimura M (1983). The Neutral Theory of Molecular Evolution.

[91] Gillespie JH (1991). The Causes of Molecular Evolution.

[92] Ohta T (1993). The nearly neutral theory of molecular evolution. Annu Rev Ecol Syst.

[93] Yang Z (1998). Likelihood ratio tests for detecting positive selection and application to primate lysozyme evolution. Mol Biol Evol.

[94] Yang Z (2007). PAML 4: phylogenetic analysis by maximum likelihood. Mol Biol Evol.

[95] Perelman P, Johnson WE, Roos C, Seuánez HN, Horvath JE, Moreira MA, Kessing B, Pontius J, Roelke M, Rumpler Y, Schneider MP, Silva A, O’Brien SJ, Pecon-Slattery J (2011). A molecular phylogeny of living primates. PLoS Genet.

[96] Nielsen R, Yang Z (1998). Likelihood models for detecting positively selected amino acid sites and applications to the HIV-1 envelope gene. Genetics.

[97] Yang Z, Nielsen R, Goldman N, Pedersen AMK (2000). Codon-substitution models for heterogeneous selection pressure at amino acid sites. Genetics.

[98] Zhang J (2000). Rates of conservative and radical nonsynonymous nucleotide substitution in mammalian nuclear genes. J Mol Evol.

[99] Bielawski JP, Yang Z, Nielsen R (2005). Maximum likelihood methods for detecting adaptive protein evolution. Statistical Methods in Molecular Evolution.

[100] Feng P, Zheng JS, Rossiter SJ, Wang D, Zhao H (2014). Massive losses of taste receptor genes in toothed and baleen whales. Genome Biol Evol.

[101] Sali A, Blundell TL (1993). Comparative protein modelling by satisfaction of spatial restraints. J Mol Biol.

[102] Marti-Renom MA, Stuart AC, Fiser A, Sánchez R, Melo F, Sali A (2000). Comparative protein structure modeling of genes and genomes. Annu Rev Biophys Biomol Struct.

[103] Kuntal BK, Polamarasetty A, Reddanna P (2010). EasyModeller: a graphical interface to MODELLER. BMC Res Notes.

[104] Muto T, Tsuchiya D, Morikawa K, Jingami H (2007). Structures of the extracellular regions of the group II/III metabotropic glutamate receptors. Proc Natl Acad Sci U S A.

[105] Trott O, Olson AJ (2010). AutoDock Vina: improving the speed and accuracy of docking with a new scoring function, efficient optimization and multithreading. J Comput Chem.

[106] Sanner MF (1999). Python: a programming language for software integration and development. J Mol Graphics Mod.

[107] Morris GM, Huey R, Lindstrom W, Sanner MF, Belew RK, Goodsell DS, Olson AJ (2009). Autodock4 and AutoDockTools4: automated docking with selective receptor flexiblity. J Comput Chem.

