# Supplementary material for: Differentiated adaptive evolution, episodic relaxation of selective constraints, and pseudogenization of umami and sweet taste genes TAS1Rs in catarrhine primates
Source: Front Zool. 2014 Oct 29;11:79. doi: 10.1186/s12983-014-0079-4 (PMC4226867; doi:10.1186/s12983-014-0079-4)
Supplement: Additional file 3: — Alignment of TAS1R1 amino acid sequences of 35 catarrhine primates. Variant residues are marked with red. [file 12983_2014_79_MOESM3_ESM.pdf]

Additional file 3. Alignment of TAS1R1 amino acid sequences of 35 catarrhine primates. Variant residues are marked with red.

Species	1 … 50 … 100
Mandrillus sphinx	MLLCTACLVGLQLLISCCWAFACHSTEPSPDFTLPGDYLLAGLFPLHSGCLQVRHRPEVTLCDRTGSFNEHGYHLFQAMRLGVEEINNSTTLLPNVTLGY
Cercocebus chrysogaster	MLLCTACLVGLQLLISCCWAFACHSTEPSPDFTLPGDYLLAGLFPLHSGCLQVRHRPEVTLCDRTGSFNEHGYHLFQAMRLGVEEINNSTTLLPNVTLGY
Papio hamadryas	MLLCTACLVGLQLLISCCWAFACHSTEPSPDFTLPGDYLLAGLFPLHSGCLQVRHRPEVTLCDRTGSFNEHGYHLFQAMRLGVEEINNSTTLLPNVTLGY
Lophocebus aterrimus	MLLCTACLVGLQLLISCCWAFACHSTEPSPDFTLPGDYLLAGLFPLHSGCLQVRHRPEVTLCDRTGSFNEHGYHLFQAMRLGVEEINNSTTLLPNVTLGY
Theropithecus gelada	MLLCTACLVGLQLLISCCWAFACHSTEPSPDFTLPGDYLLAGLFPLHSGCLQVRHRPEVTLCDRTGGFNEHGYHLFQAMRLGVEEINNSTTLLPNVTLGY
Macaca fuscata	MLLCTACLVGLQLLISCCWAFACHSTEPSPDFTLPGDYLLAGLFPLHSGCLQVRHRPEVTLCDRTGSFNEHGYHLFQAMRLGVEEINNSTTLLPNVTLGY
Macaca arctoides	MLLCTACLVGLQLLISCCWAFACHSTEPSPDFTLPGDYLLAGLFPLHSGCLQVRHRPEVTLCDRTGSFNEHGYHLFQAMRLGVEEINNSTTLLPNVTLGY
Macaca assamensis	MLLCTACLVGLQLLISCCWAFACHSTEPSPDFTLPGDYLLAGLFPLHSGCLQVRHRPEVTLCDRTGSFNEHGYHLFQAMRLGVEEINNSTTLLPNVTLGY
Chlorocebus sabaceus	MLLCTACLVGLQLLISCCWAFACHSTEPSPDFTLPGDYLLAGLFPLHSGCLQVRHRPEVTLCDRPGSFNEHGYHLFQAMRLGVEEINNSTALLPNVTLGY
Erythrocebus patas	MLLCTACLVGLQLLISCCWAFACHSTEPSPDFTLPGDYLLAGLFPLHSGCLQVRHRPEVTLCDRPGSFNEHGYHLFQAMRLGVEEINNSTALLPNVTLGY
Cercopithecus mitis	MLLCTACLVGLQLLISCCWAFACHSTEPSPDFTLPGDYLLAGLFPLHSGCLQVRHRPEVTLCDRSGSFNEHGYHLFQAMRLGVEEINNSTALLPNVTLGY
Cercopithecus albogularis	MLLCTACLVGLQLLISCCWAFACHSTEPSPDFTLPGDYLLAGLFPLHSGCLQVRHRPEVTLCDRSGSFNEHGYHLFQAMRLGVEEINNSTALLPNVTLGY
Pygathrix nigripes	MLLCTACLVGLQLLISCCWAFACHSTKPSPDFTLPGDYLLAGLFPLHSGCLQVRHRPEVTLCDRPGSFNEHGYHLFQAMRLGVEEINNSTALLPNVTLGY
Pygathrix nemaeus	MLLCTACLVGLQLLISCCWAFACHSTKPSPDFTLPGDYLLAGLFPLHSGCLQVRHRPEVTLCDRPGSFNEHGYHLFQAMRLGVEEINNSTALLPNVTLGY
Nasalis larvatus	MLLCTACLVGLQLLISCCWAFACHSTKPSPDFTLPGDYLLAGLFPLHSGCLQVRHRPEVTLCDRPGSFSEHGYHLFQAMRLGVEEINNSTALLPNVTLGY
Rhinopithecus roxellana	MLLCTACLVGLQLLISCCWAFACHSTKPSPDFTLPGDYLLAGLFPLHSGCLQVRHRPEVTLCDRPGSFNEHGYHLFQAMRLGVEEINNSTALLPNVTLGY
Rhinopithecus brelichi	MLLCTACLVGLQLLISCCWAFACHSTKPSPDFTLPGDYLLAGLFPLHSGCLQVRHRPEVTLCDRPGSFNEHGYHLFQAMRLGVEEINNSTALLPNVTLGY
Rhinopithecus bieti	MLLCTACLVGLQLLISCCWAFACHSTKPSPDFTLPGDYLLAGLFPLHSGCLQVRHRPEVTLCDRPGSFNEHGYHLFQAMRLGVEEINNSTALLPNVTLGY
Trachypithecus francoisi	MLLCTACLVGLQLLISCCWAFACHSTKPSPDFTLPGDYLLAGLFPLHSGCLQVRHRPEVTLYDRPGSFNEHGYHLFQAMRLGVEEINNSTALLPNVTLGY
Semnopithecus vetulus	MLLCTACLVGLQLLISCCWAFACHSTKPSPDFTLPGDYLLAGLFPLHSGCLQVRHRPEVTLCDRPGSFNEHGYHLFQAMRLGVEEINNSTALLPNVTLGY
Presbytis melalophos	MLLCTACLVGLQLLISHCWAFACHSTKPSPDFTLPGDYLLAGLFPLHSGCLQVRHRPEVTLCDRPGSFNEHGYHLFQAMRLGVEEINNSTALLPNVTLGY
Colobus polykomos	MLLCTACLVGLQLLISCCWAFACHSTEPSPDFILPGDYLLAGLFPLHSGCLQVRHRPEVTLCDRPASFNEHGYHLFQAMRLGVEEINNSTALLPNVTLGY
Pan troglodytes	MLLCKARLVGLQLLISCCWAFACHSTESSPDFSLHGDYLLAGLFPLHSGCLQVRHRPEVTLCDRSCSFNEHGYHLFQAMRLGVEEINNSTALLPNITLGY
Pan paniscus	MLLCKARLVGLQLLISCCWAFACHSTESSPDFTLPGDYLLAGLFPLHSGCLQVRHRPEVTLCDRSCSFNEHGYHLFQAMRLGVEEINNSTALLPNITLGY
Homo sapiens	MLLCTARLVGLQLLISCCWAFACHSTESSPDFTLPGDYLLAGLFPLHSGCLQVRHRPEVTLCDRSCSFNEHGYHLFQAMRLGVEEINNSTALLPNITLGY
Gorilla gorilla gorilla	MLLCTARLVGLQLLISCCWAFACHSTESSPDFTLPGDYLLAGLFPLHSGCLQVRHRPEVTLCDRSCSFNEHGYHLFQAMRLGVEEINNSTALLPNITLGY
Pongo abelii	MLLCTARLVGLQLLIFCCWAFACHSTESSPEFTLPGDYLLAGLFPLHSGSLQVRHRPEVTLCDRSGSFNEHGYHLFQAMRLGVEEINNSTALLPNVTLGY
Hoolock hoolock	MLLCTARLVGLQLLISWCWAFACHSTESSPDFTLPGDYLLAGLFPLHSGCLQVRHRPEVTLCDRSGSFNEHGYHLFQAMRLGVEEINNSTALLPNVTLGY
Symphalangus syndactylus	MLLCTARLVGLQLLISWCWAFACHSTESSPDFTLPGDYLLAGLFPLHSGCLQVRHRPEVTLCDRSGSFNEHGYHLFQAMRLGVEEINNSTALLPNVTLGY
Hylobates lar	MLLCTARLVGLQLLISWCWAFACHSTESSPDFTLPGDYLLAGLFPLHSGRLQVRHRPEVTLCDRSGSFNEHGYHLFQAMRLGVEEINNSTALLPNVTLGY
Hylobates abbotti	MLLCTARLVGLQLLISWCWAFACHSTESSPDFTLPGDYLLAGLFPLHSGRLQVRHRPEVTLCDRSGSFNEHGYHLFQAMRLGVEEINNSTALLPNVTLGY
Hylobates agilis	MLLCTARLVGLQLLISWCWAFACHSTESSPDFTLPGDYLLAGLFPLHSGRLQVRHRPEVTLCDRSGSFNEHGYHLFQAMRLGVEEINNSTALLPNVTLGY
Hylobates pileatus	MLLCTARLVGLQLLISWCWAFACHSTESSPDFTLPGDYLLAGLFPLHSGHLQVRHRPEVTLCDRSGSFNEHGYHLFQAMRLGVEEINNSTALLPNVTLGY
Nomascus leucogenys	MLLCTARLVGLQLLISWCWAFACHSTESSPDFTLPGDYLLAGLFPLHSGCLQVRHRPEVTLCDRSGSFNEHGYHLFQAMRLGVEEINNSTALLPNVTLGY
Nomascus annamensis	MLLCTARLVGLQLLISWCWAFACHSTESSPDFTLPGDYLLAGLFPLHSGCLQVRHRPEVTLCDRSGSFNEHGYHLFQAMRLGVEEINNSTALLPNVTLGY

	101 — 150 — 200
Mandrillus sphinx	QLYDVCSDSANVYATLSVLSLPGPRHIELQGDLLCYSPKVLAVIGPDTTNRAATTAALLSPFLMPLISYGASSETLSVKRQYPSFLRTIPNDKYQVETMV
Cercocebus chrysogaster	QLYDVCSDSANVYATLSVLSLPGPRHIELQGDLLRYSPKVLAVIGPDTTNHAATTAALLSPFLMPLISYGASSETLSVKRQYPSFLRTIPNDKYQVETMV
Papio hamadryas	QLYDVCSDSANVYATLSVLSLPGPRHIELQGDLLRYSPKVLAVIGPDTTNRAATTAALLSPFLMPLISYGASSETLSVKRQYPSFLRTIPNDKYQVETMV
Lophocebus aterrimus	QLYDVCSDSANVYATLSVLSLPGPRHIELQGDLLRYSPKVLAVIGPDTTNRAATTAALLSPFLMPLISYGASSETLSVKRQYPSFLRTIPNDKYQVETMV
Theropithecus gelada	QLYDVCSDSANVYATLSVLSLPGPRHIELQGDLLRYSPKVLAVIGPDTTNRAATTAALLSPFLMPLISYGASSETLSVKRQYPSFLRTIPNDKYQVETMV
Macaca fuscata	QLYDVCSDSANVYATLSVLSLPGPRHIELQGDLLRYSPKVLAVIGPDTTNRAATTAALLSPFLMPLISYGASSETLSVKRQYPSFLRTIPNDKYQVETMV
Macaca arctoides	QLYDVCSDSANVYATLSVLSLPGPRHIELQGDLLRYSPKVLAVIGPDTTNRAATTAALLSPFLMPLISYGASSETLSVKRQYPSFLRTIPNDKYQVETMV
Macaca assamensis	QLYDVCSDSANVYATLSVLSLPGPRHIELQGDLLRYSPKVLAVIGPDTTNRAATTAALLSPFLMPLISYGASSETLSVKRQYPSFLRTIPNDKYQVETMV
Chlorocebus sabaceus	QLYDVCSDSANVYATLRVLSLPGPRHIELQGDLLRYSPKVLAVIGPDTTNRAATTAALLSPFLMPLISYGASSETLSVKRQYPSFLRTIPSDKYQVETMV
Erythrocebus patas	QLYDVCSDSANVYATLRVLSLPGPRHIELQEDLLRYSPKVLAVIGPDTTNRAATTAALLSPFLMPLISYGASSETLSVKRQYPSFLRTIPSDKYQVETMV
Cercopithecus mitis	QLYDVCSDSANVYATLRVLSLPGPHHIELQGDLLRYSPKVLAVIGPDTTNRAATTAALLSPFLMPLISYGASSETLSVKRQYPSFLRTIPSDKYQVETMV
Cercopithecus albogularis	QLYDVCSDSANVYATLRVLSLPGPHHIELQGDLLRYSPKVLAVIGPDTTNRAATTAALLSPFLMPLISYGASSETLSVKRQYPSFLRTIPSDKYQVETMV
Pygathrix nigripes	QLYDVCSDSANVYATLRVLSLPGPRHIELQGDLLHYSPKVLAVIGPDTTNRAATTAALLSPFLMPLISYGASSETLSVKQQYPSFLRTIPNDKYQVETMV
Pygathrix nemaeus	QLYDVCSDSANVYATLRVLSLPGPRHIELQGDLLHYSPKVLAVIGPDTTNRAATTAALLSPFLMPLISYGASSETLSVKRQYPSFLRTIPNDKYQVETMV
Nasalis larvatus	QLYDVCSDSANVYATLRVLSLPGPRHIELQEDLLHYSPKVLAVIGPDTTNRAATTAALLSPFLMPLISYGASSETLSVKRQYPSFLRTIPNDKYQVETMV
Rhinopithecus roxellana	QLYDVCSDSANVYATLRVLSLPGPRHIELQGDLLHYSPKVLAVIGPDTTNRAATTAALLSPFLMPLISYGASSETLSVKRQYPSFLRTIPNDKYQVETMV
Rhinopithecus brelichi	QLYDVCSDSANVYATLRVLSLPGPRHIELQGDLLHYSPKVLAVIGPDTTNRAATTAALLSPFLMPLISYGASSETLSVKRQYPSFLRTIPNDKYQVETMV
Rhinopithecus bieti	QLYDVCSDSANVYATLRVLSLPGPRHIELQGDLLHYSPKVLAVIGPDTTNRAATTAALLSPFLMPLISYGASSETLSVKRQYPSFLRTIPNDKYQVETMV
Trachypithecus francoisi	QLYDVCSDSANVYATLRVLSLPGPRHIELQGDLLHYSPKVLAVIGPDTTNRAATTAALLSPFLMPLISYGASSETLSVKRQYPSFLRTIPNDKYQVETMV
Semnopithecus vetulus	QLYDVCSDSANVYATLRVLSLPGPRHIELQGDLLHYSPKVLAVIGPDTTSRAATTAALLSPFLMPLISYGASSETLSVKRQYPSFLRTIPNDKYQVETMV
Presbytis melalophos	QLYDVCSDSANVYATLTVLSLPGPHHIELQGDLLHYSPKVLAVIGPDTTNRAATTAALLSPFLMPLISYGASSETLSVKRQYPSFLRTIPNDKYQVETMV
Colobus polykomos	QLYDVCSDSASVYATLSVLSLPGPRHIELQGDLLHYSPKVLAVIGPDTTNRAATTAALLSPFLMPLISYGASSETLSVKRQYPSFLRTIPNDKYQVETMV
Pan troglodytes	QLYDVCSDSANVYATLRVLSLPGQHHIELQGDLLHYSPTVLAVIGPDSTNRAATTAALLSPFLVPMISYAASSETLSVKRQYPSFLRTIPNDKYQVETMV
Pan paniscus	QLYDVCSDSANVYATLRVLSLPGQHHIELQGDLLHYSPTVLAVIGPDSTNRAATTAALLSPFLVPMISYAASSETLSVKRQYPSFLRTIPNDKYQVETMV
Homo sapiens	QLYDVCSDSANVYATLRVLSLPGQHHIELQGDLLHYSPTVLAVIGPDSTNRAATTAALLSPFLVPMISYAASSETLSVKRQYPSFLRTIPNDKYQVETMV
Gorilla gorilla gorilla	QLYDVCSDSANVYATLRVLSLPGQHHIELQGDLLHYSPTVLAVIGPDSTNRAATTAALLSPFLVPMISYAASSETLSVKRQYPSFLRTIPNDKYQVETMV
Pongo abelii	QLYDVCSDSANVYATLRVLSLPGPHHIELQGDLLHYSPMVLAVIGPDSTNRAATTAALLSPFLVPMISYAASSETLSVKRQYPSFLRTIPNDKYQVETMV
Hoolock hoolock	QLYDVCSDSANVYATLRVLSLPGPHHIELQGDLLHYSPTVLAVIGPDSTNHAATTAALLSPFLVPLISYAASSETLSVKRQYPSFLRTIPNDKYQVETMV
Symphalangus syndactylus	QLYDVCSDSANVYATLRVLSLPGPHHIELQGDLLHYSPTVLAVIGPDSTNHAATTAALLSPFLVPLISYAASSETLSVKRQYPSFLRTIPNDKYQVETMV
Hylobates lar	QLYDVCSDSANVYATLRVLSLPGPHHIELQGDLLHYSPTVLAVIGPESTNHAATTAALLSPFLVPLISYAASSETLSVKRQYPSFLRTIPNDKYQVETMV
Hylobates abbotti	QLYDVCSDSANVYATLRVLSLPGPHHIELQGDLLHYSPTVLAVIGPESTNHAATTAALLSPFLVPLISYAASSETLSVKRQYPSFLRTIPNDKYQVETMV
Hylobates agilis	QLYDVCSDSANVYATLRVLSLPGPHHIELQGDLLHYSPTVLAVIGPESTNHAATTAALLSPFLVPLISYAASSETLSVKRQYPSFLRTIPNDKYQVETMV
Hylobates pileatus	QLYDVCSDSANVYATLRVLSLPGPHHIELQGDLLHYSPTVLAVIGPESTNHAATTAALLSPFLVPLISYAASSETLSVKRQYPSFLRTIPNDKYQVETMV
Nomascus leucogenys	QLYDVCSDSANVYATLRVLSLPGPHHIELQGDLLHYSPTVLAVIGPDSTNHAATTAALLSPFLVPLISYAASSETLSVKRQYPSFLRTIPNDKYQVETMV
Nomascus annamensis	QLYDVCSDSANVYATLRVLSLPGPHHIELQGDLLHYSPTVLAVIGPDSTNHAATTAALLSPFLVPLISYAASSETLSVKRQYPSFLRTIPNDKYQVETMV

201 250 300

Mandrillus sphinx	LLLQRFGWTWISLVGSSGDYGQLGVQALEKQATDQGICIAFKDIVPFSAQVGDQRMQRLMRHLAQATATVVVVFSNRPLARVFFESVVLANLTGKVWVAS
Cercocebus chrysogaster	LLLQRFGWTWISLVGSSGDYGQLGVQALEKQATDQGICIAFKDIVPFSAQVGDQRMQRLMRHLAQATATVVVVFSNRPLARVFFESVVLANLTGKVWVAS
Papio hamadryas	LLLQRFRWTWISLVGSSGDYGQLGVQALEKQATDQGICIAFKDIVPFSAQVGDQRMQRLMRHLAQATATVVVVFSNRPLARVFFESVVLANLTGKVWVAS
Lophocebus aterrimus	LLLQRFGWTWISLVGSSGDYGQLGVQALEKQATDQGICIAFKDIVPFSAQVGDQRMQRLMRHLAQATATVVVVFSNRPLARVFFESVVLANLTGKVWVAS
Theropithecus gelada	LLLQRFGWTWISLVGSSGDYGQLGVQALEKQATDQGICIAFKDIVPFSAQVGDQRMHRLMRHLAQATATVVVVFSNRPLARVFFESVVLANLTGKVWVAS
Macaca fuscata	LLLQRFGWTWISLVGSSGDYGQLGVQALEKQATDQGICIAFKDIVPFSAQVGDQRMQRLMRHLAQATATVVVVFSNRPLARVFFESVVLANLTGKVWVAS
Macaca arctoides	LLLQRFGWTWISLVGSSGDYGQLGVQALEKQATDQGICIAFKDIVPFSAQVGDQRMQRLMRHLAQATATVVVVFSNRPLARVFFESVVLANLTGKVWVAS
Macaca assamensis	LLLQRFGWTWISLVGSSGDYGQLGVQALEKQATDQGICIAFKDIVPFSAQVGDQRMQRLMRHLAQATATVVVVFSNRPLARVFFESVVLANLTGKVWVAS
Chlorocebus sabaceus	LLLQRFGWTWISLVGSSGDYGQLGVQALEKQAIDQGICIAFKDIVPFSAQVGDERMQRLMRHLAQATATVVVVFSNRPLARVFFESVVLANLTGKVWVAS
Erythrocebus patas	LLLQRFGWTWISLVGSSGDYGQLGVQALEKQATDQGICIAFKDIVPFSAQVGDERMQRLMRHLAQATATVVVVFSNRPLARVFFESVVLANLTGKVWVAS
Cercopithecus mitis	LLLQRFGWTWISLVGSSGDYGQLGVQALEKQATDQGICIAFKDIVPFSAQVGDERMQRLMRHLAQATATVVVVFSNRPLARVFFESVVLANLTGKVWVAS
Cercopithecus albogularis	LLLQRFGWTWISLVGSSGDYGQLGVQALEKQATDQGICIAFKDIVPFSAQVGDERMQRLMRHLAQATATVVVVFSNRPLARVFFESVVLANLTGKVWVAS
Pygathrix nigripes	LLLQRFGWTWISLVGSSGDYGQLGVQALEKQATDQGICIAFKDIVPFSAQVGDERMQRLMRHLAQSTATVVVVFSNRPLARVFFESVVLANLTGKVWVAS
Pygathrix nemaeus	LLLQRFGWTWISLVGSSGDYGQLGVQALEKQATDQGICIAFKDIVPFSAQVGDERMQRLMRHLAQSTATVVVVFSNRPLARVFFESVVLANLTGKVWVAS
Nasalis larvatus	LLLQRFGWTWISLVGSSGDYGQLGVQALEKQATDQGICIAFKDIVPFSAQVGDERMQRLMRHLAQATATVVVVFSNRPLARVFFESVLLANLTGKVWVAS
Rhinopithecus roxellana	LLLQRFGWTWISLVGSSGDYGQLGVQALEKQATDQGICIAFKDIVPFSAQVGDERMQRLMRHLAQATATVVVVFSNRPLARVFFESVVLANLTGKVWVAS
Rhinopithecus brelichi	LLLQRFGWTWISLVGSSGDYGQLGVQALEKQATDQGICIAFKDIVPFSAQVGDERMQRLMRHLAQATATVVVVFSNRPLARVFFESVVLANLTGKVWVAS
Rhinopithecus bieti	LLLQRFGWTWISLVGSSGDYGQLGVQALEKQATDQGICIAFKDIVPFSAQVGDERMQRLMRHLAQATATVVVVFSNRPLARVFFESVVLANLTGKVWVAS
Trachypithecus francoisi	LLLQRFGWTWISLVGSSGDYGQLGVQALEKQATDQGICIAFKDIVPFSAQVGDERMQRLMRHLAQATATVVVVFSNRPLARVFFESVVLANLTGKVWVAS
Semnopithecus vetulus	LLLQRFGWTWISLVGSSGDYGQLGVQALEKQATDQGICIAFKDIVPFSAQVGDERMQRLMRHLAQATATVVVVFSNRPLARVFFESVVLANLTGKVWVAS
Presbytis melalophos	LLLQRFGWTWISLVGSSGDYGQLGVQALEKQATDQGICIAFKDIVPFSAQVGDERMQRLMRHLAQATATVVVVFSNRPLARVFFESVVLANLTGKVWVAS
Colobus polykomos	LLLQRFGWTWISLVGSSGDYGQLGVQALEKQATDQGICIAFKDIVPFSAQVGDERMQRLMHHLAQATATVVVVFSNRPLARVFFESVVLANLTGKVWVAS
Pan troglodytes	LLLQKFGWTWISLVGSSDDYGQLGVQALENQATGQGICIAFKDIMPFSAQVGDERMQCLMRHLAQAGATVVVVFSSRQLARVFFESVVLTNLTGKVWVAS
Pan paniscus	LLLQKFGWTWISLVGSSDDYGQLGVQALENQATGQGICIAFKDIMPFSAQVGDERMQCLMRHLAQAGATVVVVFSSRQLARVFFESVVLTNLTGKVWVAS
Homo sapiens	LLLQKFGWTWISLVGSSDDYGQLGVQALENQATGQGICIAFKDIMPFSAQVGDERMQCLMRHLAQAGATVVVVFSSRQLARVFFESVVLTNLTGKVWVAS
Gorilla gorilla gorilla	LLLQKFGWTWISLVGSSDDYGQLGVQALENQATGQGICIAFKDIMPFSAQVGDERMQCIMRHLAQAGATVVVVFSSRQLARVFFESVVLTNLTGKVWVAS
Pongo abelii	LLLQKFRWTWISLVGSSDDYGQLGVQALENQATGQGICIAFKDIMPFSAQVGDERMQCLMRHLAQARATVVVVFSSRQLARVFFESVVLANLTGKVWVAS
Hoolock hoolock	LLLQKFGWTWISLVGSSDDYGQLGVQALENQATDQGICIAFKDIMPFSAHVGDERMQCLMRRLAQATATVVVVFSSRQLARVFFESVVLANLTGKVWVAS
Symphalangus syndactylus	LLLQKFGWTWISLVGSSDDYGQLGVQALENQATGQGICIAFKDIMPFSAHVGDERMQCLMRRLAQARATVVVVFSSRQLARVFFESVVLANLTGKVWVAS
Hylobates lar	LLLQKFGWTWISLVGSSDDYGQLGVQALENQATGQGICIAFKDIMPFSAHVGDERMQCLMRRLAQARATVVVVFSGRQLARVFFESVVLANLTGKVWVAS
Hylobates abbotti	LLLQKFGWTWISLVGSSDDYGQLGVQALENQATGQGICIAFKDIMPFSAHAGDERMQCLMRRLAQARATVVVVFSGRQLARVFFESVVLANLTGKVWVAS
Hylobates agilis	LLLQKFGWTWISLVGSSDDYGQLGVQALENQATGQGICIAFKDIMPFSAHVGDERMQCLMRRLAQARATVVVVFSGRQLARVFFESVVLANLTGKVWVAS
Hylobates pileatus	LLLQKFGWTWISLVGSSDDYGQLGVQALENQATGQGICIAFKDIMPFSAHVGDERMQCLMRRLAQARATVVVVFSGRQLARVFFESVVLANLTGKVWVAS
Nomascus leucogenys	LLLKKFGWTWISLVGSSDDYGQLGVQALENQATGQGICIAFKDIMPFSAHVGDERMQCLMRRLAQARATVVVVFSSRQLARVFFESVVLANLTGKVWVAS
Nomascus annamensis	LLLKKFGWTWISLVGSSDDYGQLGVQALENQATGQGICIAFKDIMPFSAHVGDERMQCLMRRLAQARATVVVVFSSRQLARVFFESVVLANLTGKVWVAS

Species	301 … 350 … 400
Mandrillus sphinx	EAWVLSRHITGVPGIQRIGTVLGVAIQKRIVPGLKAFEEAYARADKGAPRPCHKSSCCSSNQVCRECEAFTAHTMPKLKGFSMSSAYNAYRAVYAVAHGL
Cercocebus chrysogaster	EAWVLSRHITGVPGIQRIGTVLGVAIQKRTVPGLKAFEEAYVRADKGAPRPCHKSSCCSSNQVCRECEAFTAHTMPKLKGFSMSSAYNAYRAVYAVAHGL
Papio hamadryas	EAWVLSRHITGVPGIQRIGTVLGVAIQKRTVPGLKAFEEAYARADKGAPRPCHKSSCCSSNQVCRECEAFTAHTMPKLKGFSMSSAYNAYRAVYAVAHGL
Lophocebus aterrimus	EAWVLSRHITGVPGIQRIGTVLGVAIQKRTVPGLKAFEEAYARADKGAPRPCHKSSCCSSNQVCRECEAFTAHTMPKLKGFSMSSAYNAYRAVYAVAHGL
Theropithecus gelada	EAWVLSRHITGVPGIQRIGTVLGVAIQKRTVPGLKAFEEAYARADKGAPRPCHKSSCCSSNQVCRECEAFTAHTMPKLKGFSMSSAYNAYRAVYAVAHGL
Macaca fuscata	EAWVLSRHITGVPGIQRIGMVLGVAIQKRTVPGLKAFEEAYARADKGAPRPCHKSSCCSSNQVCRECEAFTAHTMPKLKGFSMSSAYNAYRAVYAVAHGL
Macaca arctoides	EAWVLSRHITGVPGIQRIGMVLGVAIQKRTVPGLKAFEEAYARADKGAPRPCHKSSCCSSNQVCRECEAFTAHTMPKLKGFSMSSAYNAYRAVYAVAHGL
Macaca assamensis	EAWVLSRHITGVPGIQRIGMVLGVAIQKRTVPGLKAFEEAYARADKGAPRPCHKSSCCSSNQVCRECEAFTAHTMPKLKGFSMSSAYNAYQAVYAVAHGL
Chlorocebus sabaceus	EAWVLSRHITGVPGIQRIGTVLGVAIQKRTVPGLKAFEEAYARADKGAPRPCHKSSCCSSNQVCRECEAFTAHTMPKLKGFSMSSAYNAYQAVYAVAHGL
Erythrocebus patas	EAWVLSRHITGVPGIQRIGTVLGVAIQKRTVPGLKAFEEAYARADKGAPRPCHKSSCCSSNQVCRECEAFTAHTMPKLKGFSMSSAYNAYQAVYAVAHGL
Cercopithecus mitis	EAWVLSRHITGVPGIQRIGTVLGVAIQKRTVPGLKAFEEAYARADKGAPRPCHKSSCCSSNQVCRECEAFTAHTMPKLKGFSMSSAYNAYCAVYAVAHGL
Cercopithecus albogularis	EAWVLSRHITGVPGIQRIGTVLGVAIQKRTVPGLKAFEEAYARADKGAPRPCHKSSCCSSNQVCRECEAFTAHTMPKLKGFSMSSAYNAYCAVYAVAHGL
Pygathrix nigripes	EAWALSRHITGVPGIQRIGTVLGVTIQKRTVPGLKAFEEAYARADKGAPRPCHKSSCCSSNQVCRECEAFTAHTMPKLKGFSMSSAYNAYQAVYAVAHGL
Pygathrix nemaeus	EAWALSRHITGVPGIQRIGTVLGVTIQKRTVPGLKAFEEAYARADKGAPRPCHKSSCCSSNQVCRECEAFTAHTMPKLKGFSMSSAYNAYQAVYAVAHGL
Nasalis larvatus	EAWALSRHITGVPGIQRIGMVLGVAIQKRTVPGLKAFEEAYARADKGAPRPCHKSSCCSSNQVCRECEAFTAHTMPKLKGFSMSSAYNAYQAVYAVAHGL
Rhinopithecus roxellana	EAWALSRHITGVPGIQRIGMVLGVAIQKRTVPGLKAFEEAYARADKGAPRPCHKSSCCSSNQVCRECEAFTAHTMPKLKGFSMSSAYNAYQAVYAVAHGL
Rhinopithecus brelichi	EAWALSRHITGVPGIQRIGMVLGVAIQKRTVPGLKAFEEAYARADKGAPRPCHKSSCCSSNQVCRECEAFTAHTMPKLKGFSMSSAYNAYQAVYAVAHGL
Rhinopithecus bieti	EAWALSRHITGVPGIQRIGMVLGVAIQKRTVPGLKAFEEAYARADKGAPRPCHKSSCCSSNQVCRECEAFTAHTMPKLKGFSMSSAYNAYQAVYAVAHGL
Trachypithecus francoisi	EAWALSRHITGVPGIQRIGMVLGVAIQKRTVPGLKAFEEAYARADKGAPRPCHKSSCCSSNQVCRECEAFTAHTMPKLKGFSMSSAYNAYQAVYAVAHGL
Semnopithecus vetulus	EAWALSRHITGVPGIQRIGMVLGVAIQKRTVPGLKAFEEAYARADKGAPRPCHKSSCCSNNQVCRECEAFTAHMMPKLKGFSMSSAYNAYQAVYAVAHGL
Presbytis melalophos	EAWVLSRHVTGVPGIQRIGTVLGVAIQKRTVPGLKAFEEAYARADKGDPRPCHKSSCCSSNQVCRECEAFTAHTMPKLKGFSMSSAYNAYRAVYAVAHGL
Colobus polykomos	EAWVLSRHITGVPGIQRIGMVLGVAIQKRTVPGLKAFEEAYARADKGAPRPCHNSSCCSSNQVCRECEAFTAHTMPKLKGFSMSSAYNAYQAVYAVAHGL
Pan troglodytes	EAWALSRHITGVPGIQRIGMVLGVAIQKRTVPRLKAFEEAYARADKEAPRPCHKGSWCSSNQLCRECQAFMAHTMPKLKAFSMSSAYNAYRAVYAVAHGL
Pan paniscus	EAWALSRHITGVPGIQRIGMVLGVAIQKRTVPRLKAFEEAYARADKEAPWPCHKGSWCSSNQLCRECQAFMAHTMPKLKAFSMSSAYNAYRAVYAVAHGL
Homo sapiens	EAWALSRHITGVPGIQRIGMVLGVAIQKRAVPGLKAFEEAYARADKEAPRPCHKGSWCSSNQLCRECQAFMAHTMPKLKAFSMSSAYNAYRAVYAVAHGL
Gorilla gorilla gorilla	EAWALSRHITGVPGIQRIGMVLGVAIQKRTVPGLKAFEEAYARADKEAPRPCHKGSWCSSNQLCRECQAFMAHTMPKLKAFSMSSAYNAYRAVYAVAHGL
Pongo abelii	EAWALSRHITGVPGIQRIGTVLGVAIQKRTVPGLKAFEEAYARADKGAPRPCHKGSWCSSNQLCRECQAFTAHKMPKLKAFSMSSAYNAYRAVYAVAHGL
Hoolock hoolock	EAWALSRHITGVPGIQRIGTVLGVAIQKRTVPGLKAFEEAYARADKGAPRPCHKGSWCSSNQLCRECQAFTAHRMPKLKAFSMSSAYNAYRAVYAVAHGL
Symphalangus syndactylus	EAWALSRHITGVPGIQRIGTVLGVAIQKRTVPGLKAFEEAYARADKGAPRPCHKGSWCSSNQLCRECQAFTAHRMPKLKAFSMSSAYNAYRAVYAVAHGL
Hylobates lar	EAWALSRHITGVPGIQRIGTVLGVAIQKRTVPGLKAFEEAYARADKGAPRPCHKGSWCSSNQLCRECQAFTAHRMPKLKAFSMSSAYNAYRAVYAVAHGL
Hylobates abbotti	EAWALSRHITGVPGIQRIGTVLGVAIQKRTVPGLKAFEEAYARADKGAPRPCHKGSWCSSNQLCRECQAFTAHRMPKLKAFSMSSAYNAYRAVYAVAHGL
Hylobates agilis	EAWALSRHITGVPGIQRIGTVLGVAIQKRTVPGLKAFEEAYARADKGAPRPCHKGSWCSSNQLCRECQAFTAHRMPKLKAFSMSSAYNAYRAVYAVAHGL
Hylobates pileatus	EAWALSRHITGVPGIQRIGTVLGVAIQKRTVPGLKAFEEAYARADKGAPRPCHKGSWCSSNQLCRECQAFTAHRMPKLKAFSMSSAYNAYRAVYAVAHGL
Nomascus leucogenys	EAWALSRHITGVPGIQRIGTVLGVAIQKRTVPGLKAFEEAYARADKGAPRPCHKGSWCSSNQLCRECQAFTAHRMPKLKAFSMSSAYNAYRAVYAVAHGL
Nomascus annamensis	EAWALSRHITGVPGIQRIGTVLGVAIQKRTVPGLKAFEEAYARADKGAPRPCHKGSWCSSNQLCRECQAFTAHRMPKLKAFSMSSAYNAYRAVYAVAHGL

	401 — 450 — 500
Mandrillus sphinx	HQLLGCASGVCSRGRVYPWQLLEQIHKVNFLLHKDTVMFNDNGDPLSSYNIIAWDWSGPKWTFTVLGSSTWSPVQLDINETKLQWHGKDNQVPKSVCSSD
Cercocebus chrysogaster	HQLLGCASGVCSRGRVYPWQLLEQIHKVNFLLHKDTVMFNDNGDPLSSYNIIAWDWSGPKWTFTVLGSSTWSPVQLNINETKIQWHGKDNQVPKSVCSSD
Papio hamadryas	HQLLGCASGVCSRGRVYPWQLLEQIHKVNFLLHKDTVMFNDNGDPLSSYNIIAWDWSGPKWTFTVLGSSTWSPVQLDINETKIQWHGKDNQVPKSVCSSD
Lophocebus aterrimus	HQLLGCASGVCSRGRVYPWQLLEQIHKVNFLLHKDTVMFNDNGDPLSSYNIIAWDWSGPKWTFTVLGSSTWSPVQLDINETKIQWHGKDNQVPKSVCSSD
Theropithecus gelada	HQLLGCASGVCSRGRVYPWQLLEQIHKVNFLLHKDTVMFNDNGDPLSSYNIIAWDWSGPKWTFTVLGSSTWSPVQLDINETKIQWHGKDNQVPKSVCSSD
Macaca fuscata	HQLLGCASGVCSRGRVYPWQLLEQIHKVNFLLHKDTVMFNDNGDPLSSYNIIAWDWSGPKWTFTVLGSSTWSPVQLDINETKIQWHGKDNQVPKSVCSSD
Macaca arctoides	HQLLGCASGVCSRGRVYPWQLLEQIHKVNFLLHKDTVMFNDNGDPLSSYNIIAWDWSGPKWTFTVLGSSTWSPVQLDINETKIQWHGKDNQVPKSVCSSD
Macaca assamensis	HQLLGCASGVCSRGRVYPWQLLEQIHKVNFLLHKDTVMFNDNGDPLSSYNIIAWDWSGPKWTFTVLGSSTWSPVQLDINETKIQWHGKDNQVPKSVCSSD
Chlorocebus sabaceus	HQLLGCASGVCSRGRVYPWQLLEQIHKVNFLLHKDTVMFNDNGDPLSSYNIIAWDWSGPKWTFTVLGSSTWSPVQLDINETKIQWHGKDNQVPKSVCSSD
Erythrocebus patas	HQLLGCASGVCSRGRVYPWQLLEQIHKVNFLLHKDTVMFNDNGDPLSSYNIIAWDWSGPKWTFTVLGSSTWSPVQLDINETKIQWHGKDNQVPKSVCSSD
Cercopithecus mitis	HQLLGCASGVCSRGRVYPWQLLEQIHKVNFLLHKDTVMFNDNGDPLSSYNIIAWDWSGPKWTFTVLGSSTWSPVQLDINETKIQWHGKDNQVPKSVCSSD
Cercopithecus albogularis	HQLLGCASGVCSRGRVYPWQLLEQIHKVNFLLHKDTVMFNDNGDPLSSYNIIAWDWSGPKWTFTVLGSSTWSPVQLDINETKIQWHGKDNQVPKSVCSSD
Pygathrix nigripes	HQLLGCASGVCSRGRVYPWQLLEQIHKVNFLLHEDTVMFNDNGDPLSSYNIIAWDWSGPKWTFTVLGSSTWYPVQLDINETKIQWHGKDNQVPKSVCSSD
Pygathrix nemaeus	HQLLGCASGVCSRGRVYPWQLLEQIHKVNFLLHEDTVMFNDNGDPLSSYNIIAWDWSGPKWTFTVLGSSTWYPVQLDINETKIQWHGKDNQVPKSVCSSD
Nasalis larvatus	HQLLGCASGVCSRGRVYPWQLLEQIHKVNFLLHEDTVMFNDNGDPLSSYNIIAWDWSGPKWTFTVLGSSTWSPVQLDINETKIQWHGKDNQVPKSVCSSD
Rhinopithecus roxellana	HQLLGCASGVCSRGRVYPWQLLEQIHKVNFLLHEDTVMFNDNGDPLSSYNIIAWDWSGPKWTFTVLGSSTWYPVQLDINETKIQWHGKDNQVPNSVCSSD
Rhinopithecus brelichi	HQLLGCASGVCSRGRVYPWQLLEQIHKVNFLLHEDTVMFNDNGDPLSSYNIIAWDWSGPKWTFTVLGSSTWYPVQLDINETKIQWHGKDNQVPNSVCSSD
Rhinopithecus bieti	HQLLGCASGVCSRGRVYPWQLLEQIHKVNFLLHEDTVMFNDNGDPLSSYNIIAWDWSGPKWTFTVLGSSTWYPVQLDINETKIQWHGKDNQVPNSVCSSD
Trachypithecus francoisi	HQLLGCASGVCSRGRVYPWQLLEQIHKVNFLLHEDTVMFNDNGDPLSSYNIIAWDWSGPKWTFTVLGSSTWSPVQLDINETKIQWHGKDNQVPKSVCSSD
Semnopithecus vetulus	HRLLGCASGVCSRGRVYPWQLLEQIHKVNFLLHEDTVMFNDNGDPLSSYNIIAWDWSGPKWTFTVLGSSTWSPVQLDINETKIQWHGKDNQVPKSVCSSD
Presbytis melalophos	HQLLGCASGVCSRGRVYPWQLLEQIHKVNFLLHEDTVMFNDNGDPLSSYNIIAWDWSGPKWTFTVLGSSTWSPVQLDINETKIQWHGKDNQVPKSVCSSD
Colobus polykomos	HQLLGCASGVCSRGRVYPWQLLEQIHKVNFLLHEDTVMFNDNGDPLSSYNIIAWDWSGPKWTFTVLGSSTWSPVQLDINETKIQWHGKDNQVPKSVCSSD
Pan troglodytes	HQLLGCASGACSRGRVYPWQLLEQIHKVHFLLHKDTVAFNDNGDPLSSYNIIAWDWNGPKWTFTVLGSSTWSPVQLNINETKIQWHGKDNQVPKSVCSSD
Pan paniscus	HQLLGCASGACSRGRVYPWQLLEQIHKVHFLLHKDTVAFNDNGDPLSSYNIIAWDWNGPKWTFTVLGSSTWSPVQLNINETKIQWHGKDNQVPKSVCSSD
Homo sapiens	HQLLGCASGACSRGRVYPWQLLEQIHKVHFLLHKDTVAFNDNRDPLSSYNIIAWDWNGPKWTFTVLGSSTWSPVQLNINETKIQWHGKDNQVPKSVCSSD
Gorilla gorilla gorilla	HQLLGCASGACSRGRVYPWQLLEQIHKVHFLLHKDTVAFNDNRDPLSSYNIIAWDWNGPKWTFTVLGSSTWSPVQLNINETKIQWHGKDNQVPKSVCSSD
Pongo abelii	HQLLGCASGACSRGRVYPWQLLEQIHKVNFLLHKDTVTFNDNRDPLSSYNIIAWDWNGPKWTFTVLGSSTWSPVQLNINETKIQWHGKDNQVPKSVCSSD
Hoolock hoolock	HQLLGCASGACSRGRVYPWQLLEQIYKVNFLLHKDTVMFDDNRDPLSSYNIIAWDWNGPKWTFTVLGSSTWSPVQLNINETKIQWHGKNNQVPKSVCSSD
Symphalangus syndactylus	HQLLGCASGACSRGRVYPWQLLEQIYKVNFLLHKDTVMFDDNRDPLSSYNIIAWDWNGPKWTFTVLGSSTWSPVQLNINETKIQWHGKNNQVPKSVCSSD
Hylobates lar	HQLLGCASGACSRGRVYPWQLLEQIYKVNFLLHKDTVMFDDNRDPLSSYNIIAWDWNGPKWTFTVLGSSTWSPVQLNINETKIQWHGKNNQVPKSVCSSD
Hylobates abbotti	HQLLGCASGACSRGRVYPWQLLEQIYKVNFLLHKDTVMFDDNRDPLSSYNIIAWDWNGPKWTFTVLGSSTWSPVQLNINETKIQWHGKNNQVPKSVCSSD
Hylobates agilis	HQLLGCASGACSRGRVYPWQLLEQIYKVNFLLHKDTVMFDDNRDPLSSYNIIAWDWNGPKWTFTVLGSSTWSPVQLNINETKIQWHGKNNQVPKSVCSSD
Hylobates pileatus	HQLLGCASGACSRGRVYPWQLLEQIYKVNFLLHKDTVMFDDNRDPLSSYNIIAWDWNGPKWTFTVLGSSTWSPVQLNINETKIQWHGKNNQVPKSVCSSD
Nomascus leucogenys	HQLLGCASGACSRGRVYPWQLLEQIYKVNFLLHKDTVMFDDNRDPLSSYNIIAWDWNGPKWTFTVLGSSTWSPVQLNINETKIQWHGKNNQVPKSVCSSD
Nomascus annamensis	HQLLGCASGACSRGRVYPWQLLEQIYKVNFLLHKDTVMFDDNRDPLSSYNIIAWDWNGPKWTFTVLGSSTWSPVQLNINETKIQWHGKNNQVPKSVCSSD

501 550 600

Mandrillus sphinx	CLEGHQRVVTGFHPCCFECVPCGAGTFLNKSDLYRCQPCGKEEWAPEGSQTCFPRTVVFLACQEHTSWVLLAANTLLLLLLLGTAGLFAWHLDTPVVRSA
Cercocebus chrysogaster	CLEGHQRVVTGFHPCCFECVPCGAGTFLNKSDLYRCQPCGKEEWAPEGSQTCFPRTVVFLACQEHTSWVLLAANTLLLLLLLGTAGLFAWHLDTPVVRSA
Papio hamadryas	CLEGHQRVVTGFHPCCFECVPCGAGTFLNKSDLYRCQPCGKEEWAPEGSQTCFPRTVVFLACQEHTSWVLLAANTLLLLLLLGTAGLFAWHLDTPVVRSA
Lophocebus aterrimus	CLEGHQRVVTGFHPCCFECVPCGAGTFLNKSDLYRCQPCGKEEWAPEGSQTCFPRTVVFLACQEHTSWVLLAANTLLLLLLLGTAGLFAWHLDTPVVRSA
Theropithecus gelada	CLEGHQRVVTGFHPCCFECVPCGAGTFLNKSDLYRCQPCGKEEWAPEGSQTCFPRTVVFLACQEHTSWVLLAANTLLLLLLLGTAGLFAWHLDTPVVRSA
Macaca fuscata	CLEGHQRVVTGFHPCCFECVPCGAGTFLNKSDLYRCQPCGKEEWAPEGSQTCFPRTVVFLACQEHTSWVLLAANTLLLLLLLGTAGLFAWHLDTPVVRSA
Macaca arctoides	CLEGHQRVVTGFHPCCFECVPCGAGSFLNKSDLYRCQPCGKEEWAPEGSQTCFPRTVVFLACQEHSSWVLLAANTLLLLLLLGTAGLFAWHLDTPVVRSA
Macaca assamensis	CLEGHQRVVTGFHPCCFECVPCGAGTFLNKSDLYRCQPCGKEEWAPEGSQTCFPRTVVFLACQEHSSWVLLAANTLLLLLLLGTAGLFAWHLDTPVVRSA
Chlorocebus sabaceus	CLEGHQRVVTGFHHCCFECVPCGAGTFLNKSDLYRCQPCGKEEWAPEGSQTCFPRTVVFLACQEHTSWVLLAANTLLLLLLLGTAGLFAWHLDTPVVRSA
Erythrocebus patas	CLEGHQRVVTGFHHCCFECVPCGAGTFLNKSDLYRCQPCGKEEWAPEGSQTCFPRTVVFLACQEHTSWVLLAANTLLLLLLLGTAGLFAWHLDTPVVRSA
Cercopithecus mitis	CLEGHQRVVTGFHHCCFECVPCGAGTFLNKSDLYRCQPCGKEEWAPEGSQTCFPRTVVFLACQEHTSWVLLAANTLLLLLLLGTAGLFAWHLDTPVVRSA
Cercopithecus albogularis	CLEGHQRVVTGFHHCCFECVPCGAGTFLNKSDLYRCQPCGKEEWAPEGSQTCFPRTVVFLACQEHTSWVLLAANTLLLLLLLGTAGLFAWHLDTPVVRSA
Pygathrix nigripes	CLEGHQRVVTGFHHCCFECVPCGAGTFLNKSDLYRCQPCGKEEWAPEGSQTCFPRTVVFLACQEHTSWVLLAANTLLLLLLLGTAGLFAWHLDTPVVRSA
Pygathrix nemaeus	CLEGHQRVVTGFHHCCFECVPCGAGTFLNKSDLYRCQPCGKEEWAPEGSQTCFPRTVVFLACQEHTSWVLLAANTLLLLLLLGTAGLFAWHLDTPVVRSA
Nasalis larvatus	CLEGHQRVVTGFHHCCFECVPCGAGTFLNKSDLYRCQPCGKEEWAPEGSQTCFPRTVVFLACQEHTSWMLLAANTLLLLLLLGTAGLFAWHLDTPVVRSA
Rhinopithecus roxellana	CLEGHQRVVTGFHHCCFECVPCGAGTFLNKSDLYRCQPCGKEEWAPEGSQTCFPRTVVFLACQEHTSWVLLAANTLLLLLLLGTAGLFAWHLDTPVVRSA
Rhinopithecus brelichi	CLEGHQRVVTGFHHCCFECVPCGAGTFLNKSDLYRCQPCGKEEWAPEGSQTCFPRTVVFLACQEHTSWVLLAANTLLLLLLLGTAGLFAWHLDTPVVRSA
Rhinopithecus bieti	CLEGHQRVVTGFHHCCFECVPCGAGTFLNKSDLYRCQPCGKEEWAPEGSQTCFPRTVVFLACQEHTSWVLLAANTLLLLLLLGTAGLFAWHLDTPVVRSA
Trachypithecus francoisi	CLEGHQRVVTGFHHCCFECVPCGAGTFLNKSDLYRCQPCGKEEWAPEGSQTCFPRTVVFLACQEHTSWVLLAANTLLLLLLLGTAGLFAWHLDTPVVRSA
Semnopithecus vetulus	CLEGHQRVVTGFHHCCFECVPCGAGTFLNKSDLYRCQPCGKEEWAPEGSQTCFPRTVVFLACQEHTSWVLLAANTLLLLLLLGTAGLFAWHLDTPVVRSA
Presbytis melalophos	CLEGHQRVVTGFHHCCFECVPCGAGTFLNKSDLYRCQPCGKEEWAPEGSQTCFPRTVVFLACQEHTSWVLLAANTLLLLLLLGTAGLFAWHLDTPVVRSA
Colobus polykomos	CLEGHQRVVTDFHRCCFECVPCGAGTFLNKSDLYRCQPCGKEEWAPEGSQTCFPRTVVFLACQEHTSWVLLAANTLLLLLLLGTAGLFAWHLDTPVVRSA
Pan troglodytes	CLEGHQRVVTGFHHCCFECVPCGAGTFLNKSDLYRCQPCGKEEWAPEGSQTCFPRTVVFLALHEHTSWVLLAANTLLLLLLLGTAGLFAWHLDTPVVRSA
Pan paniscus	CLEGHQRVVTGFHHCCFECVPCGAGTFLNKSDLYRCQPCGKEEWAPEGSQTCFPRTVVFLALHEHTSWVLLAANTLLLLLLLGTAGLFAWHLDTPVVRSA
Homo sapiens	CLEGHQRVVTGFHHCCFECVPCGAGTFLNKSDLYRCQPCGKEEWAPEGSQTCFPRTVVFLALREHTSWVLLAANTLLLLLLLGTAGLFAWHLDTPVVRSA
Gorilla gorilla gorilla	CLEGHQRVVTGFHHCCFECVPCGAGTFLNKSDLYRCQPCGKEEWAPEGSQTCFPRTVVFLALHEHTSWVLLAANTLLLLLLLGTAGLFAWHLDTPVVRSA
Pongo abelii	CLEGHQRVVMGFHHCCFECVPCGAGTFLNKSDLYRCQPCGKEEWAPEGSQTCFPRTVVFLALHEHTSWVLLAANTLLLLLLLGTAGLFAWHLDTPVVRSA
Hoolock hoolock	CLEGYQRVVMGFHHCCFECVPCGAGTFLNKSDLYRCQPCGKEEWAPEGSQTCFLRTVVFLAWHEHTTWVLLAANTVLLLLLLGTAGLFAWHLDTPVVRSA
Symphalangus syndactylus	CLEGYQRVVMGFHHCCFECVPCGAGTFLNKSDLYRCQPCGKEEWAPEGSQTCFLRTVVFLAWHEHTSWVLLAANTALLLLLLGTAGLFAWHLDTPVVRSA
Hylobates lar	CLEGYQRVVTGFHHCCFECVPCGAGTFLNKSDLYRCQPCGKEEWAPEGSQTCFVRTVVFLAWHEHTSWVLLAANTVLLLLLLGTAGLFAWHLDTPVVRSA
Hylobates abbotti	CLEGYQRVVTGFHHCCFECVPCGAGTFLNKSDLYRCQPCGKEEWAPEGSQTCFLRTVVFLAWHEHTSWVLLAANTVLLLLLLGTAGLFAWHLDTPVVRSA
Hylobates agilis	CLEGYQRVVTGFHHCCFECVPCGAGTFLNKSDLYRCQPCGKEEWAPEGSQTCFLRTVVFLAWHEHTSWVLLAANTVLLLLLLGTAGLFAWHLDTPVVRSA
Hylobates pileatus	CLEGYQRVVTGFHHCCFECVPCGAGTFLNKSDLYRCQPCGKEEWAPEGSQTCFLRTVVFLAWHEHTSWVLLAANTVLLLLLLGTAGLFAWHLDTPVVRSA
Nomascus leucogenys	CLEGYQRVVMGFHHCCFECVPCGAGTFLNKSDLYRCQPCGKEEWAPEGSQTCFLRTVAFLAWHEHTSWVLLAANTVLLLLLLGTAGLFAWHLDTPVVRSA
Nomascus annamensis	CLEGYQRVVMGVHHCCFECVPCGAGTFLNKSDFYRCQPCGKEEWAPEGSQTCFLRTVAFLAWHEHTSWVLLAANTVLLLLLLGTAGLFAWHLDTPVVRSA

	601 — 650 — 700
Mandrillus sphinx	GGRLCFLMLGSLAAGSGSLYGFFGEPTRPACLLRQALFALGFTIFLSCLTVRSFQLIIIFKFSTKAPTFYRAWVQNHGAGLFVMISSAAQLLICLTWLVV
Cercocebus chrysogaster	GGRLCFLMLGSLAAGSGSLYGFFGEPTRPACLLRQALFALGFTIFLSCLTVRSFQLIIIFKFSTKAPTFYRAWVQNHGAGLFVMISSAAQLLICLTWLVV
Papio hamadryas	GGRLCFLMLGSLAAGSGSLYGFFGEPTRPACLLRQALFALGFTIFLSCLTVRSFQLIIIFKFSTKAPTFYRAWVQNHGAGLFVMISSAAQLLICLTWLVV
Lophocebus aterrimus	GGRLCFLMLGSLAAGSGSLYGFFGEPTRPACLLRQALFALGFTIFLSCLTVRSFQLIIIFKFSTKAPTFYRAWVQNHGAGLFVMISSAAQLLICLTWLVV
Theropithecus gelada	GGRLCFLMLGSLAAGSGSLYGFFGEPTRPACLLRQALFALGFTIFLSCLTVRSFQLIIIFKFSTKAPTFYRAWVQNHGAGLFVMISSAAQLLICLTWLVV
Macaca fuscata	GGRLCFLMLGSLAAGSGSLYGFFGEPTRPACLLRQALFALGFTIFLSCLTVRSFQLIIIFKFSAKAPTFYRVWVQNHGAGLFVMISSAAQLLICLTWLVV
Macaca arctoides	GGRLCFLMLGSLAAGSGSLYGFFGEPTRPACLLRQALFALGFTIFLSCLTVRSFQLIIIFKFSAKAPTFYRVWVQNHGAGLFVMISSAAQLLICLTWLVV
Macaca assamensis	GGRLCFLMLGSLAAGSGSLYGFFGEPTRPACLLRQALFALGFTIFLSCLTVRSFQLIIIFKFSAKAPTFYRVWVQNHGAGLFVMISSAAQLLICLTWLVV
Chlorocebus sabaceus	GGHLCFLMLGSLAAGSGSLYGFFGEPTRPACLLRQALFALGFTIFLSCLTVRSFQLIIIFKFSTKAPTFYRAWVQNHGAGLFVMISSAAQLLICLTWLVV
Erythrocebus patas	GGRLCFLMLGSLAAGSGSLYGFFGEPTRPACLLRQALFALGFTIFLSCLTVRSFQLIIIFKFSTKAPTFYRAWVQNHGAGLFVMISSAAQLLICLTWLVV
Cercopithecus mitis	GGRLCFLMLGSLAAGSGSLYGFFGEPTRPACLLRQALFALGFTIFLSCLTVRSFQLIIIFKFSTKAPTFYRAWVQNHGAGLFVMISSAAQLLICLTWLVV
Cercopithecus albogularis	GGRLCFLMLGSLAAGSGSLYGFFGEPTRPACLLRQALFALGFTIFLSCLTVRSFQLIIIFKFSTKAPTFYRAWVQNHGAGLFVMISSAAQLLICLTWLVV
Pygathrix nigripes	GGRLCFLMLGSLAAGSSSLYGFFGEPTRPACLLRQALFALGFTIFLSCLTVRSFQLIIIFKFSTKAPTLYHAWVQNHGAGLFVMISSAAQLLICLTWLVV
Pygathrix nemaeus	GGRLCFLMLGSLAAGSSSLYGFLGEPTRPACLLRQALFALGFTIFLSCLTVRSFQLIIIFKFSTKAPTLYHAWVQNHGAGLFVMISSAAQLLICLTWLVV
Nasalis larvatus	GGRLCFLMLGSLAAGSSSLYGFFGEPTRPACLLRQALFALGFTIFLSCLTVRSFQLIIIFKFSTKAPTFYHAWVQNHGAGLFVMISSAAQLLICLTWLVV
Rhinopithecus roxellana	GGRLCFLMLGSLAAGSSSLYGFFGEPTRPACLLRQALFALGFTIFLSCLTVRSFQLIIIFKFSTKAPTFYHAWVQNHGAGLFVMIISASQLLICLTWLVV
Rhinopithecus brelichi	GGRLCFLMLGSLAAGSSSLYGFFGEPTRPACLLRQALFALGFTIFLSCLTVRSFQLIIIFKFSTKAPTFYHAWVQNHGAGLFVMIISASQLLICLTWLVV
Rhinopithecus bieti	GGRLCFLMLGSLAAGSSSLYGFFGEPTRPACLLRQALFALGFTIFLSCLTVRSFQLIIIFKFSTKAPTFYHAWVQNHGAGLFVMIISASQLLICLTWLVV
Trachypithecus francoisi	GGRLCFLMLGSLAAGSSSLYGFFGEPTRPACLLRQALFALGFTIFLSCLTVRSFQLIIIFKFSTKAPTFYHAWVQNHGAGLFVMISSAAQLLICLTWLVV
Semnopithecus vetulus	GGRLCFLMLGSLAAGSSSLYGFFGEPTRPACLLRQALFALGFTIFLSCLTVRSFQLIIIFKFSTKAPTFYHAWVQNHGAGLFVMISSAAQLLICLTWLVV
Presbytis melalophos	GGRLCFLMLGSLAAGSSSLYGFFGEPTRPACLLRQALFALGFTIFLSCLTVRSFQLIIIFKFSTKAPTFYHAWVQNHGAGLFVMISSAAQLLICLTWLVV
Colobus polykomos	GGRLCFLMLGSLAAGSSSLYGFFGEPTRPACLLRQALFALGFTIFLSCLTVRSFQLIIIFKFSTKAPTFYHAWVQNHGAGLFVMISSAAQLLICLTWLVV
Pan troglodytes	GGRLCFLMLGSLAAGSGSLYGFFGEPTRPACLLRQALFALGFTIFLSCLTVRSFQLIIIFKFSTKVPTFYHAWVQNHGAGLFVMISSVAQLLICLTWLVV
Pan paniscus	GGRLCFLMLGSLAAGSGSLYGFFGEPTRPACLLRQALFALGFTIFLSCLTVRSFQLIIIFKFSTKVPTFYHAWVQNHGAGLFVMISSVAQLLICLTWLVV
Homo sapiens	GGRLCFLMLGSLAAGSGSLYGFFGEPTRPACLLRQALFALGFTIFLSCLTVRSFQLIIIFKFSTKVPTFYHAWVQNHGAGLFVMISSAAQLLICLTWLVV
Gorilla gorilla gorilla	GGRLCFLMLGSLAAGSGSLYGFFGEPTRPACLLRQALFALGFTIFLSCLTVRSFQLIIIFKFSTKVPTFYHAWVQNHGAGLFVMISSAAQLLICLTWLVV
Pongo abelii	GGRLCFLMLGSLAAGSGSLYGFFGEPTRPACLLRQALFALGFTTFLSCLTVRSFQLIIIFKFSIKVPTFYHAWVQNHGAGLFVIISSAAQLLICLTWLVV
Hoolock hoolock	GGRLCFLMLGSLAAGSGSLYGFFGEPTRPACLLRQALFALGFTIFLSCLTVRSFQLIIIFKFSTKVPTFYHAWVQNHGAGLFVMISSAAQLLICLTWLVV
Symphalangus syndactylus	GGRLCFLMLGSLAAGSGSLYGFFGEPTTPACLLRQALFALGFTIFLSCLTVRSFQLIIIFKFSTKVPTFYHAWVQNHGAGLFVMISSAAQLLICLTWLVV
Hylobates lar	GGRLCFLMLGSLAAGSGSLYGFFGEPTRPACLLRQALFALGFTIFLSCLTVRSFQLIIIFKFSTKVPTFYHAWVQNHGAGLFVMISSAAQLLICLTWLVV
Hylobates abbotti	GGRLCFLMLGSLAAGSGSLYGFFGEPTRPACLLRQALFALGFTIFLSCLTVRSFQLIIIFKFSTKVPTFYHAWVQNHGAGLFVMISSAAQLLICLTWLVV
Hylobates agilis	GGRLCFLMLGSLAAGSGSLYGFFGEPTRPACLLRQALFALGFTIFLSCLTVRSFQLIIIFKFSTKVPTFYHAWVQNHGAGLFVMISSAAQLLICLTWLVV
Hylobates pileatus	GGRLCFLMLGSLAAGSGSLYGFFGEPTRPACLLRQALFALGFTIFLSCLTVRSFQLIIIFKFSTKVPTFYHAWVQNHGAGLFVMISSAAQLLICLTWLVV
Nomascus leucogenys	GGRLCFLMLGSLAAGSGSLYGFFGEPTRPACLLRQALFALGFTIFLSCLTVRSFQLIIIFKFSTKVPTFYHAWVQNHGAGLFVMISSAAQLLICLTWLVV
Nomascus annamensis	GGRLCFLMLGSLAAGSGSLYGFFGEPTRPACLLRQALFALGFTIFLSCLTVRSFQLIIIFKFSTKVPTFYHSWVQNHGAGLFVMISSAAQLLICLTWLVV

	701 750 800
Mandrillus sphinx	WTPLPTREYQRFPHLVMLECTEANSLGFILAFLYNGLLSISAFACSYLGKDLPENYNEAKCVTFSLLFNFMSWIAFFTTASVYDGKYLPAVNMLAGLSSL
Cercocebus chrysogaster	WTPLPTREYQRFPHLVMLECTEANSLGFILAFLYNGLLSISAFACSYLGKDLPENYNEAKCVTFSLLFNFMSWIAFFTTASVYDGKYLPAVNMLAGLSSL
Papio hamadryas	WTPLPTREYQRFPHLVMLECTEANSLGFILAFLYNGLLSISAFACSYLGKDLPENYNEAKCVTFSLLFNFMSWIAFFTTASVYDGKYVPAVNMLAGLSSL
Lophocebus aterrimus	WTPLPTREYQRFPHLVMLECTEANSLGFILAFLYNGLLSISAFACSYLGKDLPENYNEAKCVTFSLLFNFMSWIAFFTTASIYDGKYVPAVNMLAGLSSL
Theropithecus gelada	WTPLPTREYQRFPHLVMLECTEANSLGFILAFLYNGLLSISAFACSYLGKDLPENYNEAKCVTFSLLFNFMSWIAFFTTASVYDGKYVPAVNMLAGLSSL
Macaca fuscata	WTPLPTREYQRFPHLVMLECTEANSLGFILAFLYNGLLSISAFACSYLGKDLPENYNEAKCVTFSLLFNFMSWIAFFTTASVYDGKYLPAVNMLAGLSSL
Macaca arctoides	WTPLPTREYQRFPHLVMLECTEANSLGFILAFLYNGLLSISAFACSYLGKDLPENYNEAKCVTFSLLFNFMSWIAFFTTASVYDGKYLPAVNMLAGLSSL
Macaca assamensis	WTPLPTREYQRFPHLVMLECTEANSLGFILAFLYNGLLSISAFACSYLGKDLPENYNEAKCVTFSLLFNFMSWIAFFTTASVYDGKYLPSVNMLAGLSSL
Chlorocebus sabaceus	WTPLPTREYQRFPHLVMLECTEANSLGFILAFLYNGLLSISAFACSYLGKDLPENYNEAKCVTFSLLFNFMSWIAFFTTASVYDGKYLPAVNMLAGLSSL
Erythrocebus patas	WTPLPTREYQRFPHLVMLECTEANSLGFILAFLYNGLLSISAFACSYLGKDLPENYNEAKCVTFSLLFNFMSWIAFFTTASVYDGKYLPAVNMLAGLSSL
Cercopithecus mitis	WTPLPTREYQRFPHLVMLECTEANSLGFILAFLYNGLLSISAFACSYLGKDLPENYNEAKCVTFSLLFNFMSWIAFFTTASVYDGKYLPAVNMLAGLSSL
Cercopithecus albogularis	WTPLPTREYQRFPHLVMLECTEANSLGFILAFLYNGLLSISAFACSYLGKDLPENYNEAKCVTFSLLFNFMSWIAFFTTASVYDGKYLPAVNMLAGLSSL
Pygathrix nigripes	WTPLPTREYQRFPHLVMLECTEANSLGFILAFLYNGLLSISAFACSYLGKDLPENYNEAKCVTFSLLFNFMSWIAFFTTASIYDGKYLPAVNMLAGLSSL
Pygathrix nemaeus	WTPLPTREYQRFPHLVMLECTEANSLGFILAFLYNGLLSISAFACSYLGKDLPENYNEAKCVTFSLLFNFMSWIAFFTTASIYDGKYLPAVNMLAGLSSL
Nasalis larvatus	WTPLPTREYQRFPHLVMLECTEANSLGFILAFLYNGLLSISAFACSYLGKDLPENYNEAKCVTFSLLFNFMSWIAFFTTASIYEGKYLPAVNMLAGLSSL
Rhinopithecus roxellana	WTPLPTREYQRFPHLVMLECTEANSLGFILAFLYNGLLSISAFACSYLGKDLPENYNEAKCVTFSLLFNFMSWIAFFTTASIYDGKYLPAVNMLAGLSSL
Rhinopithecus brelichi	WTPLPTREYQRFPHLVMLECTEANSLGFILAFLYNGLLSISAFACSYLGKDLPENYNEAKCVTFSLLFNFMSWIAFFTTASIYDGKYLPAVNMLAGLSSL
Rhinopithecus bieti	WTPLPTREYQRFPHLVMLECTEANSLGFILAFVYNGLLSISAFACSYLGKDLPENYNEAKCVTFSLLFNFMSWIAFFTTASIYDGKYLPAVNMLAGLSSL
Trachypithecus francoisi	WTPLPTREYQRFPHLVMLECTEANSLGFILAFLYNGLLSISAFACSYLGKDLPENYNEAKCVTFSLLFNFMSWIAFFTTASIYDGKYLPAVNMLAGLSSL
Semnopithecus vetulus	WTPLPTREYQRFPHLVMLECTEANSLGFILAFLYNGLLSISAFACSYLGKDLPENYNEAKCVTFSLLFNFMSWIAFFTTASIYDGKYLPAVNMLAGLSSL
Presbytis melalophos	WTPLPTREYQRFPHLVMLECTEANSLGFILAFLYNGLLSISAFACSYLGKDLPENYNEAKCVTFSLLFNFMSWIAFFTTASIYDGKYLPAVNMLAGLSSL
Colobus polykomos	WTPLPTREYQRFPHLVMLECTEANSLGFILAFLYNGLLSISAFACSYLGKDLPENYNEAKCVTFSLLFNFMSWIAFFTTASIYDGKYLPAFNMLAGLSSL
Pan troglodytes	WTPLPTREYQRFPHLVMLECTETNSLGFILAFLYNGLLSISAFACSYLGKDLPENYNEAKCVTFSLLFNFVSWIAFFTTASVYDGKYLPAANMMAGLSSL
Pan paniscus	WTPLPTREYQRFPHLVMLECTETNSLGFILAFLYNGLLSISAFACSYLGKDLPENYNEAKCVTFSLLFNFVSWIAFFTTASVYDGKYLPAANMMAGLSSL
Homo sapiens	WTPLPAREYQRFPHLVMLECTETNSLGFILAFLYNGLLSISAFACSYLGKDLPENYNEAKCVTFSLLFNFVSWIAFFTTASVYDGKYLPAANMMAGLSSL
Gorilla gorilla gorilla	WTPLPTREYQRFPHLVMLDCTETNSLGFILAFLYNGLLSISAFACSYLGKDLPENYNEAKCVTFSLLFNFVSWIAFFTTASVYDGKYLPAANMMAGLSSL
Pongo abelii	WTPLPTREYQRFPHLVMLECTEANSLGFILAFLYNGLLSISAFACSYLGKDLPENYNEAKCVTFSLLFNFVSWIAFFTTASVYDGKYLPAANMMAGLSSL
Hoolock hoolock	WTPLPTREYQRFPHLVMLECTEANSLGFILAFLYNGLLSISAFACSYLGKDLPENYNEAKCVTFSLLFNFVSWIAFFTTASVYDGKYLPAANMMAGLSSL
Symphalangus syndactylus	WTPLPTREYQRFPHLVMLECTEANSLGFILAFLYNGLLSISAFACSYLGKDLPENYNEAKCVTFSLLFNFVSWIAFFTTASVYDGKYLPAANMMAGLSSL
Hylobates lar	WTPLPTREYQRFPRLVMLECTEANSLGFILAFLYNGLLSISAFACSYLGKDLPENYNEAKCVTFSLLFNFVSWIAFFTTASVYDGKYLPAANMMAGLSSL
Hylobates abbotti	WTPLPTREYQRFPRLVMLECTEANSLGFILAFLYNGLLSISAFACSYLGKDLPENYNEAKCVTFSLLFNFVSWIAFFTTASVYDGKYLPAANMMAGLSSL
Hylobates agilis	WTPLPTREYQRFPRLVMLECTEANSLGFILAFLYNGLLSISAFACSYLGKDLPENYNEAKCVTFSLLFNFVSWIAFFTTASVYDGKYLPAANMMAGLSSL
Hylobates pileatus	WTPLPTREYQRFPRLVMLECTEANSLGFILAFLYNGLLSISAFACSYLGKDLPENYNEAKCVTFSLLFNFVSWIAFFTSASVYDGKYLPAANMMAGLSSL
Nomascus leucogenys	WTPLPTREYQRFPHLVMLECTEANSLGFILAFLYNGLLSISAFACSYLGKDLPENYNEAKCVTFSLLFNFVSWIAFFTTASVYDGKYLPAVNMMAGLSSL
Nomascus annamensis	WTPLPTREYQRFPHLVMLECTEANSLGFILAFLYNGLLSISAFACSYLGKDLPENYNEAKCVTFSLLFNFVSWIAFFTTASVYDGKYLPAVNMMAGLSSL

	801 — 841
Mandrillus sphinx	SSGFGGYFLPKCYVILCRPDLNSTEHFQASIQDYTRHRGST
Cercocebus chrysogaster	SSGFGGYFLPKCYVILCRPDLNSTEHFQASIQDYTRHRGST
Papio hamadryas	SSGFGGYFLPKCYVILCRPDLNSTEHFQASIQDYTRHRGST
Lophocebus aterrimus	SSGFGGYFLPKCYVILCRPDLNSTEHFQASIQDYTRHRGST
Theropithecus gelada	SSGFGGYFLPKCYVILCRPDLNSTEHFQASIQDYTRHRGST
Macaca fuscata	SSGFGGYFLPKCYVILCRPDLNSTEHFQASIQDYTRHRGST
Macaca arctoides	SSGFGGYFLPKCYVILCRPDLNSTEHFQASIQDYTRHRGST
Macaca assamensis	SSGFGGYFLPKCYVILCRPDLNSREHFQASIQDYTRHRGST
Chlorocebus sabaceus	SSGFGGYFLPKCYVILCRPDLNSTEHFQASIQDYTRHRGST
Erythrocebus patas	SSGFGGYFLPKCYVILCRPDLNSTEHFQASIQDYTRHRGST
Cercopithecus mitis	SSGFGGYFLPKCYVILCRPDLNSTEHFQASIQDYTRHRGST
Cercopithecus albogularis	SSGFGGYFLPKCYVILCRPDLNSTEHFQASIQDYTRHRGST
Pygathrix nigripes	SSGFGGYFLPKCYVILCRPDLNSTEHFQASIQDYTRHRGST
Pygathrix nemaeus	SSGFGGYFLPKCYVILCRPDLNSTEHFQASIQDYTRHRGST
Nasalis larvatus	SSGFGGYFLPKCYVILCRPDLNSTEHFQASIQDYTRHRGST
Rhinopithecus roxellana	SSGFGGYFLPKCYVILCRPDLNSTEHFQASIQDYTRHRGST
Rhinopithecus brelichi	SSGFGGYFLPKCYVILCRPDLNSTEHFQASIQDYTRHRGST
Rhinopithecus bieti	SSGFGGYFLPKCYVILCRPDLNSTEHFQASIQDYTRHRGST
Trachypithecus francoisi	SSGFGGYFLPKCYVILCRPDLNSTEHFQASIQDYTRHRGTT
Semnopithecus vetulus	SSGFGGYFLPKCYVILCRPDLNSTEHFQASIQDYTRHRGST
Presbytis melalophos	SSGFGGYFLPKCYVILCRPDLNSTEHFQASIQDYTRHRGST
Colobus polykomos	SSGFGGYFLPKCYVILCRPDLNSTEHFQASIQDYTRHRGST
Pan troglodytes	SSGFGGYFLPKCYVILYRPDLNSTEHFQASIQDYTRRCGST
Pan paniscus	SSGFGGYFLPKCYVILYRPDLNSTEHFQASIQDYTRRCGST
Homo sapiens	SSGFGGYFLPKCYVILCRPDLNSTEHFQASIQDYTRRCGST
Gorilla gorilla gorilla	SSGFGGYFLPKCYVILCRPDLNSTEHFQASIQDYTRRCGST
Pongo abelii	GSGFGGYFLPKCYVILCRPDLNSTEHFQASIQDYTRRCGST
Hoolock hoolock	SSGFGGYFLPKCYVILCRPDLNSTEHFQASIQDYTRRCGST
Symphalangus syndactylus	SSGFGGYFLPKCYVILCRPDLNSTEHFQASIQDYTRRCGST
Hylobates lar	SSGFGGYFLPKCYVILCRPDLNSTEHFQASIQDYTRRCGST
Hylobates abbotti	SSGFGGYFLPKCYVILCRPDLNSTEHFQASIQDYTRRCGST
Hylobates agilis	SSGFGGYFLPKCYVILCRPDLNSTEHFQASIQDYTRRCGST
Hylobates pileatus	SSGFGGYFLPKCYVILCRPDLNSTEHFQASIQDYTRRCGST
Nomascus leucogenys	SSGFGGYFLPKCYVILCRPDLNSTEHFQASIQDYTRRCGST
Nomascus annamensis	SSGFGGYFLPKCYVILCRPDLNSTEHFQASIQDYTRRCGST
