# Supplementary material for: Differentiated adaptive evolution, episodic relaxation of selective constraints, and pseudogenization of umami and sweet taste genes TAS1Rs in catarrhine primates
Source: Front Zool. 2014 Oct 29;11:79. doi: 10.1186/s12983-014-0079-4 (PMC4226867; doi:10.1186/s12983-014-0079-4)
Supplement: Additional file 4: — Alignment of TAS1R2 amino acid sequences of 35 catarrhine primates. Variant residues are marked with red. [file 12983_2014_79_MOESM4_ESM.pdf]

Additional file 4. Alignment of TAS1R2 amino acid sequences of 35 catarrhine primates. Variant residues are marked with red.

	1 ... 50 ... 100
Macaca arctoides	MRPRATTICSLFFLLRVLAEPAKNSDFYLPGDYLLGGLFTLHANMKGIVHLDYLQVPMCKEYETKVIGYNLMQAMRFAVEEINNDSSLLPDVLLGYEMVD
Macaca fuscata	MRPRATTICSLFFLLWVLAEPAKNSDFYLPGDYLLGGLFTLHANMKGIVHLDYLQVPMCKEYETKVIGYNLMQAMRFAVEEINNDSSLLPDVLLGYEMVD
Macaca assamensis	MRPRATTICSLFFLLRVLAEPAKNSDFYLPGDYLLGGLFTLHANMKGIVHLDYLQVPMCKEYETKVIGYNLMQAMRFAVEEINNDSSLLPDVLLGYEMVD
Cercocebus chrysogaster	MRPRATTICSLFFLLQVLAEPAKNSDFYLPGDYLLGGLFTLHANMKGIVHLDYLQVPMCKEYETKVIGYNLMQAMRFAVEEINNDSSLLPDVLLGYEMVD
Mandrillus sphinx	MRPRATTICSLFFLLRVLAEPAKNSDFYLPGDYLLGGLFTLHANMKGIVHLDYLQVPMCKEYETKVIGYNLMQAMRFAVEEINNDSSLLPDVLLGYEMVD
Lophocebus aterrimus	MRPRATTICSLFFLLRVLAEPAKNSDFYLPGDYLLGGLFTLHANMKGIVHLDYLQVPMCKEYETKVIGYNLMQAMRFAVEEINNDSSLLPDVLLGYEMVD
Papio hamadryas	MRPRATTICSLFFLLRVLAEPAKNSDFYLPGDYLLGGLFTLHANMKGIVHLDYLQVPMCKEYETKVIGYNLMQAMRFAVEEINNDSSLLPDVLLGYEMVD
Theropithecus gelada	MRPRETTICSLFFLLRVLAEPAKNSDFYLPGDYLLGGLFTLHANVKGIVHLDYLQVPMCKEYETKVIGYNLMQAMRFAVEEINNDSSLLPDVLLGYEMVD
Cercopithecus mitis	MRPRATTICSLFFLLQVLAEPAKNSDFYLPGDYLLGGLFTLHANVKGIVHLDYLQVPMCKEYETKVIGYNLMQAMRFAVEEINNDSSLLPDVRLGYEMVD
Cercopithecus albogularis	MRPRATTICSLFFLLQVLAEPAKNSDFYLPGDYLLGGLFTLHANVKGIVHLDYLQVPMCKEYETKVIGYNLMQAMRFAVEEINNDSSLLPDVRLGYEMVD
Erythrocebus patas	MRPRATTICSLFFLLQVLAEPAKNSDFYLPGDYLLGGLFTLHANMKGIVHLDYLQVPMCKEYETKVIGYNLMQAMRFAVEEINNDSSLLPDVLLGYEMVD
Chlorocebus sabaceus	MRPRATTICSLFFLLQVLAEPAKNSDFYLPGDYLLGGLFTLHANMKGIVHLDYLQVPMCKEYETKVIGYNLMQAMRFAVEEINNDSSLLPDVLLGYEMVD
Colobus polykomos	MRPRATTVCSLFFLLRVLAEPAKNSDFYLPGDYLLGGLFTLHANVKGIVHLDFLQVPMCKEYETKVMGYNLMQAMRFAVEEINNDSSLLPDVLLGYEMVD
Presbytis melalophos	MWPRATTICSLFFLLRVLAEPAKNSDFYLPGDYLLGGLFTLHANVKGIVHLDFLQVPMCKEYETKVMGYNLMQAMRFAVEEINNDSSLLPDVLLGYEMVD
Trachypithecus francoisi	MRPRATAFCSLFFLLRVLAEPAKNSDFYLPGDYLLGGLFTLHANMKGIVHLNFLQVPMCKEYETKVMGYNLMQAMRFAVEEINNDSSLLPDVLLGYEMVD
Semnopithecus vetulus	MRPRATAICFLFFLLRVLAEPAKNSDFYLPGDYLLGGLFTLHANMKGIVHLNFLQVPMCKEYETKVMGYNLMQAMRFAVEEINNDSSLLPDVLLGYEMVD
Pygathrix nigripes	MRPRATTLCSLFFLLRVLAEPAKNSDFYLPGDYLLGGLFTLHANMKGIVHLDFLQVPMCKEYETKVMGYNLMQAMRFAVEEINNDSSLLPDVLLGYEMVD
Pygathrix nemaeus	MRPRATTLCSLFFLLRVLAEPAKNSDFYLPGDYLLGGLFTLHANMKGIVHLDFLQVPMCKEYETKVMGYNLMQAMRFAVEEINNDSSLLPDVLLGYEMVD
Nasalis larvatus	MPPRATTICSLFFLLRVLAEPAKNSDFYLPGDYLLGGLFTLHANMKGIVHLDFLQVPMCKEYETKVMGYNLMQAMRFAVEEINNDSSLLPDVLLGYEMVD
Rhinopithecus bieti	MRPRATTICSLFFLLRVLAEPAKNSDFYLPGDYLLGGLFTLHANMKGIVHLDFLQVPMCKEYETKVMGYNLMQAMRFAVEEINNDSSLLPDVLLGYEMVD
Rhinopithecus brelichi	MRPRATTICSLFFLLRVLAEPAKNSDFYLPGDYLLGGLFTLHANMKGIVHLDFLQVPMCKEYETKVMGYNLMQAMRFAVEEINNDSSLLPDVLLGYEMVD
Rhinopithecus roxellana	MRPRATTICSLFFLLRVLAEPAKNSDFYLPGDYLLGGLFTLHANMKGIVHLDFLQVPMCKEYETKVMGYNLMQAMRFAVEEINNDSSLLPDVLLGYEMVD
Hoolock hoollock	MGPRAMTICSLFFLLRILSESAENSDFYLPGDYLLGGLFSLHANMKGIVHLNFLQVPMCKEYEVKVIGYNLMQAMRFAVEEINNDSSLLPGVLLGYEIVD
Symphalangus syndactylus	MGPRAMTICSLFFLLRILSEPAENSDFYLPGDYLLGGLFSLHANMKGIVHLNFLQVPMCKEYEVKVIGYNLMQAMRFAVEEINNDSSLLPGVLLGYEIVD
Hylobates lar	MGPRATTICSLFFLLRILSESAENSDFYLPGDYLLGGLFSLHANMKGIVHLNFLQVPMCKEYEVKVIGYNLMQAMRFAVEEINNDSNLLPGVLLGYEIVD
Hylobates abbotti	MGPRATTICSLFFLLRILSESAENSDFYLPGDYLLGGLFSLHANMKGIVHLNFLQVPMCKEYEVKVIGYNLMQAMRFTVEEINNDSNLLPGVLLGYEIVD
Hylobates agilis	MGPRATTICSLFFLLRILSESAENSDFYLPGDYLLGGLFSLHANMKGIVHLNFLQVPMCKEYEVKVIGYNLMQAMRFTVEEINNDSNLLPGVLLGYEIVD
Hylobates pileatus	MGPRATTICSLFFLLRILSESAENSDFYLPGDYLLGGLFSLHANMKGIVHLNFLQVPTCKEYEVKVIGYNLMQAMRFAVEEINNDSNLLPGVLLGYEIVD
Nomascus leucogenys	MGPRATTICSLFFLLRILSEPAENSDFYLPGDYLLGGLFSLHANMKGIVHLNFLQVPMCKEYEVKVIGYNLMQAMRFAVEEINNDSSLLPGVLLGYEIVD
Nomascus annamensis	MGPRATTICSLFFLLRILSEPAENSDFYLPGDYLLGGLFSLHANMKGIVHLNFLQVPMCKEYEVKVIGYNLMQAMRFAVEEINNDSSLLPGVLLGYEIVD
Pan paniscus	MGPRAKTICSLFFLLWVLAEPAENSDFYLPGDYLLGGLFSLHANMKGIVHLNFLQVPMCKEYEVKVIGYNLMQAMRFAVEEINNDSSLLPGVLLGYEIVD
Pan troglodytes	MGPRAKTICSLFFLLWVLAEPAENSDFYLPGDYLLGGLFSLHANMKGIVHLNFLQVPMCKEYEVKVIGYNLMQAMRFAVEEINNDSSLLPGVLLGYEIVD
Homo sapiens	MGPRAKTICSLFFLLWVLAEPAENSDFYLPGDYLLGGLFSLHANMKGIVHLNFLQVPMCKEYEVKVIGYNLMQAMRFAVEEINNDSSLLPGVLLGYEIVD
Gorilla gorilla gorilla	MGTRATTICSLFFLLWVLAEPAENSDFYLPGDYLLGGLFSLHANMKGIVHLNFLQVPMCKEYEVKVIGYNLMQAMRFAVEEINNDSSLLPGVLLGYEIVD
Pongo pygmaeus	MGPRATTICSLFFLLWVLAEPAENSDFYLPGDYLLGGLFSLHANMKGIVHLNFLQVPMCKEYEVKVIGYNLMQDMRFSVEEINNDSSLLPGVLLGYEMVD

101 150 200

Macaca arctoides	VCYVSNNVQPVLYFLAQEDDLLPIQENYSNYVPRVVAVIGPDNSDAVMTVANFLSLFLLPQITYSAISDELRDKVRFPALLRTAPSADHHIEAMVQLMLH
Macaca fuscata	VCYVSNNVQPVLYFLAQEDDLLPIQENYSNYVPRVVAVIGPDNSDAVMTVANFLSLFLLPQITYSAISDELRDKVRFPALLRTAPSADHHIEAMVQLMLH
Macaca assamensis	VCYVSNNVQPVLYFLAQEDDLLPIQENYSNYVPRVVAVIGPDNSDAVMTVANFLSLFLLPQITYSAISDELRDKVRFPALLRTAPSADHHIEAMVQLMLH
Cercocebus chrysogaster	VCYVSNNVQPVLYFLAQEDDLLPIQENYSNYVPRVVAVIGPDNSDAVMTVANFLSLFLLPQITYSAISDELRDKVRFPALLRTAPSADHHIEAMVQLMLH
Mandrillus sphinx	VCYVSNNVQPVLYFLAQEDDLLPIQENYSNYMPRVVAVIGPDNSDAVMTVANFLSLFLLPQITYSAISDELRDKVRFPALLRTAPSADHHIEAMVQLMLH
Lophocebus aterrimus	VCYVSNNVQPVLYFLAQEDDLLPIQENYSNYVPRVVAVIGPDNSDAVMTVANFLSLFLLPQITYSAISDELRDKVRFPALLRTAPSADHHIEAMVQLMLH
Papio hamadryas	VCYVSNNVQPVLYFLAQEDDLLPIQENYSNYVPRVVAVIGPDNSDAVMTVANFLSLFLLPQITYSAISDELRDKVRFPALLRTAPSADHHIEAMVQLMLH
Theropithecus gelada	VCYVSNNVQPVLYFLAQEDDLLPIQENYSNYVPRVVAVIGPDNSDAVMTVANFLSLFLLPQITYSAISDELRDKVRFPALLRTAPSADHHIEAMVQLMLH
Cercopithecus mitis	VCYISNNVQPVLYFLAQEDDLLPIQENYSNYVPRVVAVIGPDNSDAVMTVANFLSLFLLPQITYSAISDELRDKVRFPALLRTAPSADHHIEAMVQLMLY
Cercopithecus albogularis	VCYISNNVQPVLYFLAQEDDLLPIQENYSNYVPRVVAVIGPDNSDAVMTVANFLSLFLLPQITYSAISDELRDKVRFPALLRTAPSADHHIEAMVQLMLY
Erythrocebus patas	VCYVSNNVQPVLYFLAQEDDLLPIQENYSNYVPRVVAVIGPDNSDAVMTVANFLSLFLLPQITYSAISDELRDKVRFPALLRTAPSADHHIEAMVQLMLY
Chlorocebus sabaceus	VCYVSNNVQPVLYFLAQEDDLLPIQENYSNYVPRVVAVIGPDNSDAVMTVANFLSLFLLPQITYSAISDELRDKVRFPALLRTAPSADHHIEAMVQLMLH
Colobus polykomos	VCYISNNVQPVLYFLAQDNNLLPIQEDYSNYMPRVVAVIGPDNSNAVMTVANFLSLFLLPQITYSANSDELRNKVRFPALLRTTPSADHHIEAMVQLMLH
Presbytis melalophos	VCYISNNVQPVLYFLAQDNDLLPIQEDYSNYMPRVVAVIGPDNSDAVMTVANFLSLFLLPQITYSAISDELRDKVRFPALLRTTPSADHHIEAMVQLMLH
Trachypithecus francoisi	VCYISNNVQPVLYFLAQDNDLLPIQEDYSNYMPRVVAVIGPDNSDAVMTVANFLSLFLLPQITYSAISDELRNKVRFPALLRTTPSADHHIEAMVQLMLH
Semnopithecus vetulus	VCYISNNVQPVLYFLAQDNDLLPIQEDYSNYMPRVVAVIGPDNSDAVVTVANFLSLFLLPQITYSANSDELRNKVRFPALLRTTPSADHHIEAMVQLMLH
Pygathrix nigripes	VCYISNNVQPVLYFLAQDNNLLPIHEDYSNYMPRVVAVIGPDNSDAVMTVANFLSLFLLPQITYSAISDGLRDKVRFPALLRTTPSADHHIEAMVQLMLH
Pygathrix nemaeus	VCYISNNVQPVLYFLAQDNNLLPIHEDYSNYMPRVVAVIGPDNSDAVMTVANFLSLFLLPQITYSAISDGLRDKVRFPALLRTTPSADHHIEAMVQLMLH
Nasalis larvatus	VCYISNNVQPVLYFLAQDNDLLPIQEDYSNYMPRVVAVIGPDNSDAVITVANFLSLFLLPQITYSAISDGLRDKVRFPALLRTIPSADHHIEAMVQLMLH
Rhinopithecus bieti	VCYISNNVQPVLYFLAQDNNLLPIHEDYSNYMPRVVAVIGPDNSDAVMTVANFLSLFLLPQITYSAISDGLRDKVRFPALLRTTPSADHHIEAMVQLMLH
Rhinopithecus brelichi	VCYISNNVQPVLYFLAQDNNLLPIHEDYSNYMPRVVAVIGPDNSDAVMTVANFLSLFLLPQITYSAISDGLRDKVRFPALLRTTPSADHHIEAMVQLMLH
Rhinopithecus roxellana	VCYISNNVQPVLYFLAQDNNLLPIHEDYSNYMPRVVAVIGPDNSDAVMTVANFLSLFLLPQITYSAISDGLRDKVRFPALLRTTPSADHHIEAMVQLMLH
Hoolock hoollock	VCYISNNVQPVLYFLAHEDNLLPIQEDYSNYSSRVVAVIGPDNSESVMTVANFLSLFLLPQITYSAISDELRDKARFPALLRTTPSADHHIEAMVQLMLH
Symphalangus syndactylus	VCYISNNVQPVLYFLAHEDNLLPIQEDYSNYSSRVVAVIGPDNSESVLTVANFLSLFLLPQITYSAISDELRDKARFPALLRTTPSADHHIEAMVQLMLH
Hylobates lar	VCYISNNVQPVLYFLAHEDNLLPIQEDYSNYSSRVVAVIGPDNSESVTTVANFLSLFLLPQITYSAISDELRDKARFPALLRTTPSADHHIEAMVQLMLH
Hylobates abbotti	VCYISNNVQPVLYFLAHEDNLLPIQEDYSNYSSRVVAVIGPDNSESVMTVANFLSLFLLPQITYSAIGDELRDKARFPALLRTTPSADHHIEAMVQLMLH
Hylobates agilis	VCYISNNVQPVLYFLAHEDNLLPIQEDYSNYSSRVVAVIGPDNSESVMTVANFLSLFLLPQITYSAIGDELRDKARFPALLRTTPSADHHIEAMVQLMLH
Hylobates pileatus	VCYISNNVQPVLYFLAHEDNLLPIQEDYSNYSSRVVAVIGPDNSESVTTVANFLSLFLLPQITYSAISDELRDKARFPALLRTTPSADHHIEAMVQLMLH
Nomascus leucogenys	VCYISNNVQPVLYFLAHEDNLLPIQEDYSNYSSRVVAVIGPDNSESVITVANFLSLFLLPQITYSAISDELRDKVRFPALLRTTPSADHHIEAMVQLMLH
Nomascus annamensis	VCYISNNVQPVLYFLAHEDNLLPIQEDYSNYSSRVVAVIGPDNSESVMTVANFLSLFLLPQITYSAISDELRDKVRFPALLRTTPSADHHIEAMVQLMLH
Pan paniscus	VCYISNNVQPVLYFLAHEDNLLPIQEDYSNYISRVVAVIGPDNSESVMTVANFLSLFLLPQITYSAIGDELRDKVRFPALLRTTPSADHHVEAMVQLMLH
Pan troglodytes	VCYISNNVQPVLYFLAHEDNLLPIQEDYSNYISRVVAVIGPDNSESVMTVANFLSLFLLPQITYSAIGDELRDKVRFPALLRTTPSADHHVEAMVQLMLH
Homo sapiens	VCYISNNVQPVLYFLAHEDNLLPIQEDYSNYISRVVAVIGPDNSESVMTVANFLSLFLLPQITYSAISDELRDKVRFPALLRTTPSADHHVEAMVQLMLH
Gorilla gorilla gorilla	VCYISNNVQPVLYFLAHEDNLLPIQEDYSNYISRVVAVIGPDNSESVMTVANFLSLFLLPQITYSAISDELQDKVRFPALLRTTPSADHHVEAMVQLMLH
Pongo pygmaeus	VCYVSNNVQPVLYFLAHEDNLLPIQEDYSDYVSRVVAVIGPDNSESVMTVANFLSLFLLPQITYSAISDELRDKVRFPALLRTTPSADHHIEAMVQLMLH

Species	201 — 250 — 300
Macaca arctoides	FRWNWIIVLVSGDTYGRDNGQLLGDRLARGDICIAFQETLPTVQPNQNMTSEERQRLVTIVDKLQQSTARVVVVFSPDLTLYNFFNEVLRQNFTGAVWIA
Macaca fuscata	FRWNWIIVLVSGDTYGRDNGQLLGDRLARGDICIAFQETLPTVQPNQNMTSEERQRLVTIVDKLQQSTARVVVVFSPDLTLYNFFNEVLRQNFTGAVWIA
Macaca assamensis	FRWNWIIVLVSGDTYGRDNGQLLGDRLARGDICIAFQETLPTVQPNQNMTSEERQRLVTIVDKLQQSTARVVVVFSPDLTLYNFFNEVLRQNFTGAVWIA
Cercocebus chrysogaster	FHWNWIIVLVSGDTYGRDNGQLLGDRLARGDICIAFQETLPTVQPNQNMTSEERQRLVTIVDKLQQSTARVVVVFSPDLTLYNFFNEVLRQNFTGAVWIA
Mandrillus sphinx	FRWNWIIVLVSGDTYGRDNGQLLGDRLARGDICIAFQETLPTVQPNQNMTSEERQRLVTIVDKLQQSTARVVVVFSPDLTLYNFFNEVLRQNFTGAVWIA
Lophocebus aterrimus	FRWNWIIVLVSGDTYGRDNGQLLGDRLARGDICIAFQETLPTVQPNQNMTSEERQRLVTIVDKLQQSTARVVVVFSPDLTLYDFFNEVLRQNFTGAVWIA
Papio hamadryas	FRWNWIIVLVSGDTYGRDNGQLLGDRLARGDICIAFQETLPTVQPNQNMTSEERQRLVTIVDKLQQSTARVVVVFSPDLTLYNFFNEVLRQNFTGAVWIA
Theropithecus gelada	FRWNWIIVLVSGDTYGRDNGQLLGDRLARGDICIAFQETLPTVQPNQNMTSEERQRLVTIVDKLQQSTARVVVVFSPDLTLYNFFNEVLRQNFTGAVWIA
Cercopithecus mitis	FHWNWIIVLVSGDTYGRDNGQLLGDRLARGDICIAFQETLPTVQPNQNMTSEERQRLVTIVDKLQQSTARVVVVFSPDLTLYNFFNEVLRQNFTGAVWIA
Cercopithecus albogularis	FHWNWIIVLVSGDTYGRDNGQLLGDRLARGDICIAFQETLPTVQPNQNMTSEERQRLVTIVDKLQQSTARVVVVFSPDLTLYNFFNEVLRQNFTGAVWIA
Erythrocebus patas	FHWNWIIVLVSGDTYGRDNGQLLGDRLARGDICIAFQETLPTVQPNQNMTSEERQRLVTIVDKLQQSTARVVVVFSPDLTLYNFFNEVLRQNFTGAVWIA
Chlorocebus sabaceus	FRWNWIIVLVSGDTYGRDNGQLLGDRLARGDICIAFQETLPTVQPNQNMTSEERQRLVTIVDKLQQSTARVVVVFSPDLTLYNFFNEVLRQNFTGAVWIA
Colobus polykomos	FRWNWIIVLVSSDTYGRDNGQLLGSRLARGDICIAFQETLPTVQPNQNMTSEERQRLVTIVDKLQQSTARVVVVFSPDLTLYNFFSEVLRQNFTGAVWIA
Presbytis melalophos	FRWNWIIVLVSSDTYGRDNGQLLGNRLARGDICIAFQETLPTVQPNQNMTSEERQRLVTIVDKLQQSTARVVVVFSPDLTLYNFFNEVLRQNFTGAVWIA
Trachypithecus francoisi	FRWNWIIVLVSSDTYGRDNGQLLGNRLARGDICIAFQETLPTVQPNQNMTSEERQRLVTIVDKLQQSTARVVVVFSPDLTLYNFFNEVLRQNFTGAVWIA
Semnopithecus vetulus	FRWNWIIVLVSSDTYGRDNGQLLGNRLARGDICIAFQETLPTVQPNQNMTSEERQRLVTIVDKLQQSTARVVVVFAPDLTLYNFFNEVLRQNFTGAVWIA
Pygathrix nigripes	FRWNWIVVLVSSDTYGRDNGQLLGNRLARGDICIAFQETLPTVQPNQNMTSEEHQRLVTIVDKLQQSTARVVVVFSPDLTLYNFFNEVLRQNFTGAVWIA
Pygathrix nemaeus	FRWNWIVVLVSSDTYGRDNGQLLGNRLARGDICIAFQETLPTVQPNQNMTSEEHQRLVTIVDKLQQSTARVVVVFSPDLTLYNFFNEVLRQNFTGAVWIA
Nasalis larvatus	FRWNWIIVLVSSDTYGRDNGQLLGNRLARGDICIAFQETLPTVQPNQNMTSEEHQRLVTIVDKLQQSTARVVVVFSPDLTLYNFFNEVLRQNFTGAVWIA
Rhinopithecus bieti	FRWNWIIVLVSSDTYGRDNGQLLGNRLARGDICIAFQETLPTMQPNQNMTSEERQRLVTIVDKLQQSTARVVVVFSPDLTLYNFFNEVLRQNFTGAVWIA
Rhinopithecus brelichi	FRWNWIIVLVSSDTYGRDNGQLLGNRLARGDICIAFQETLPTMQPNQNMTSEERQRLVTIVDKLQQSTARVVVVFSPDLTLYNFFNEVLRQNFTGAVWIA
Rhinopithecus roxellana	FRWNWIIVLVSSDTYGRDNGQLLGNRLARGDICIAFQETLPTMQPNQNMTSEERQRLVTIVDKLQQSTARVVVVFSPDLTLYNFFNEVLRQNFTGAVWIA
Hoolock hoollock	FHWNWIIVLVSSDTYGRDNGQLLGERLARRDICIAFQETLPTLQPNQNMTSDERQRLVTIVDKLQQSTARVVVVFSPDLTLYDFFNEVLRQNFTGAVWIA
Symphalangus syndactylus	FRWNWIIVLVSSDTYGRDNGQLLGERLARRDICIAFQETLPTLQPNQNMTSDERQRLVTIVDKLQQSTARVVVVFSPDLTLYDFFNEVLRQNFTGAVWIA
Hylobates lar	FRWNWIIVLVSSDTYGRDNGQLLGERLARRDICIAFQETLPTLQPNQNMTSDERQRLVTIVDKLQQSTARVVVVFSPDLTLYDFFNEVLRQNFTGAVWIA
Hylobates abbotti	FRWNWIIVLVSSDTYGRDNGQLLGERLARRDICIAFQETLPTLQPNQNMTSDERQRLVTIVDKLQQSTARVVVVFSPDLTLYDFFNEVLRQNFTGAVWIA
Hylobates agilis	FRWNWIIVLVSSDTYGRDNGQLLGERLARRDICIAFQETLPTLQPNQNMTSDERQRLVTIVDKLQQSTARVVVVFSPDLTLYDFFNEVLRQNFTGAVWIA
Hylobates pileatus	FRWNWIIVLVSSDTYGRDNGQLLGERLARRDICIAFQETLPTLQPNQNMTSDERQRLVTIVDKLQQSTARVVVVFSPDLTLYDFFNEVLRQNFTGAVWIA
Nomascus leucogenys	FRWNWIIVLVSSDTYGRDNGQLLGERLARRDICIAFQETLPTLQPNQNMTSDERQRLVTIVDKLQQSTARVVVVFSPDLTLYDFFNEVLRQNFTGAVWIA
Nomascus annamensis	FRWNWIIVLVSSDTYGRDNGQLLGERLARRDICIAFQETLPTLQPNQNMTSDERQRLVTIVDKLQQSTARVVVVFSPDLTLYDFFNEVLRQNFTGAVWIA
Pan paniscus	FRWNWIIVLVSSDTYGRDNGQLLGERLARRDICIAFQETLPALQPNQNMTSEERQRLVTIVDKLQQSTARVVVVFSPDLSLYDFFNEVLRQNFTGAVWIA
Pan troglodytes	FRWNWIIVLVSSDTYGRDNGQLLGERLARRDICIAFQETLPALQPNQNMTSEERQRLVTIVDKLQQSTARVVVVFSPDLSLYDFFNEVLRQNFTGAVWIA
Homo sapiens	FRWNWIIVLVSSDTYGRDNGQLLGERVARRDICIAFQETLPTLQPNQNMTSEERQRLVTIVDKLQQSTARVVVVFSPDLTLYHFFNEVLRQNFTGAVWIA
Gorilla gorilla gorilla	FRWNWIIVLVSSDTYGRDNGQLLGERLARRDICIAFQETLPTLQPNQNMTSEERQRLVTIVDKLQQSTARVVVVFSPDLSLYDFFNEVLRQNFTGAVWIA
Pongo pygmaeus	FRWNWIIVLVSSDTYGRDNGQLLGERLARRDICIAFQETLPTLQPNQNMTSEERQRLVTIVDKLQQSTARVVVVFSPDLTLYDFFNEVLRQNFTGAVWIA

	301 350 400
Macaca arctoides	SESWAIDPVLHNLTELRHMGTFLGITIQSVPIPGFSEFRVRDPQAGPPPLSRTSQRSTCNQECDSCLNGTLSFNNVLRLSGERVVYSVYSAVYAVAHALH
Macaca fuscata	SESWAIDPVLHNLTELRHMGTFLGITIQSVPIPGFSEFRVRDPQAGPPPLSRTSQRSTCNQECDSCLNGTLSFNNVLRLSGERVVYSVYSAVYAVAHALH
Macaca assamensis	SESWAIDPVLHNLTELRHMGTFLGITIQSVPIPGFSEFRVRDPQAGPPPLSRTSQRSTCNQECDSCLNGTLSFNNVLRLSGERVVYSVYSAVYAVAHALH
Cercocebus chrysogaster	SESWAIDPVLHNLTELRHMGTFLGITIQSVPIPGFSEFRVRDPQAGPPPLSRTSQRSTCNQECDSCLNGTLSFNNVLRLSGERVVYSVYAAVYAVAHALH
Mandrillus sphinx	SESWAIDPVLHNLTELRHMGTFLGITIQSVPIPGFSEFRVRDPQAGPPPLSRTSQRSTCNQECDSCLNGTLSFNNVLRLSGERVVYSVYSAVYAVAHALH
Lophocebus aterrimus	SESWAIDPVLHNLTELRHMGTFLGITIQSVPIPGFSEFRVRDPQAGPPPLSRTSQRSTCNQECDSCLNGTLSFNNVLRLSGERVVYSVYSAVYAVAHALH
Papio hamadryas	SESWAIDPVLHNLTELRHMGTFLGITIQSVPIPGFSEFRVRDPQAGPPPLSRTSQRSTCNQECDSCLNGTLSFNNVLRLSGERVVYSVYSAVYAVAHALH
Theropithecus gelada	SESWAIDPVLHNLTELRHVGTFLGITIQSVPIPGFSEFRVRDPQAGPPPLSRTSQRSTCNQECDSCLNGTLSFNNVLRLSGERVVYSVYSAVYAVAHALH
Cercopithecus mitis	SESWAIDPVLHNLTELRHMGTFLGITIQSVPIPGFSEFRVRDPQAGPPPLSRTSQRSTCNQECDSCLNGTLSFDNVLRLSGERVVYSVYSAVYAVAHALH
Cercopithecus albogularis	SESWAIDPVLHNLTELRHMGTFLGITIQSVPIPGFSEFRVRDPQAGPPPLSRTSQRSTCNQECDSCLNGTLSFDNVLRLSGERVVYSVYSAVYAVAHALH
Erythrocebus patas	SESWAIDPVLHNLTELRHMGTFLGITIQSVPIPGFSEFRVRDPQAGPPPLSRSSQRSTCNQECDSCLNGTLSFDNVLRLSGERVVYSVYSAVYAVAHALH
Chlorocebus sabaceus	SESWAIDPVLHNLTELRHMGTFLGITIQSVPIPGFSEFRVRDPQAGPPPLSRTSQRSTCNQECDSCLNGTLSFDNVLRLSGERVVYSVYSAVYAVAHALH
Colobus polykomos	SESWAIDPVLHNLTELRHTGTFLGITIQSVPIPGFSEFRVRGPQAGPSPLSRTSQRSTCNQECDNCLNGTLSFNTVLRLSGERVVYSVYSAVYAVAHALH
Presbytis melalophos	SESWAIDPVLHNLTELRHMGTFLGITIQSVPIPGFSEFRVRGPQAGPSPLSRTSQRSTCNQECDNCLNGTLSFNTILRLSGERVVYSVYSAVYAVAHALH
Trachypithecus francoisi	SESWAIDPVLHNLTELRHMGTFLGITIQSVPIPGFSEFRVQGPQAGPSPLSRTSQRSTCNQECDNCLNDTLSFNTVLRLSGERVVYSVYSAVYAVAHALH
Semnopithecus vetulus	SESWAIDPVLHNLTELRHMGTFLGITIQSVPIPGFSEFRVQGPQAGPSPLSRTSQRSTCNQECDNCLNDTLSFNTVLRLSGERVVYSVYSAVYAVAHALH
Pygathrix nigripes	SESWAIDPVLHNLTELRHVGTFLGITIQNVPIPGFSEFRVRGPQAGPSPLSRTSQRSTCNQECDNCLNGTLSFNTVLRLSGERVVYSVYSAVYAVAHALH
Pygathrix nemaeus	SESWAIDPVLHNLTELRHVGTFLGITIQNVPIPGFSEFRVRGPQAGPSPLSRTSQRSTCNQECDNCLNGTLSFNTVLRLSGERVVYSVYSAVYAVAHALH
Nasalis larvatus	SESWAIDPVLHNLTELRHMGTFLGITIQSVPIPGFSEFRVRDPQAGPSPLSRTSQRSTCNQECDNCLNGTLSFNTVLRLSGERVVYSVYSAVYAVAHALH
Rhinopithecus bieti	SESWAIDPVLHNLTELRHIGTFLGITIQSVPIPGFSEFRVRGPQAGPSPLSRTSQRSTCNQECDNCLNGTLSFNTVLRLSGERVVYSVYSAVYAVAHALH
Rhinopithecus brelichi	SESWAIDPVLHNLTELRHIGTFLGITIQSVPIPGFSEFRVRGPQAGPSPLSRTSQRSTCNQECDNCLNGTLSFNTVLRLSGERVVYSVYSAVYAVAHALH
Rhinopithecus roxellana	SESWAIDPVLHNLTELRHIGTFLGITIQSVPIPGFSEFRVRGPQAGPSPLSRTSQRSTCNQECDNCLNGTLSFNTVLRLSGERVVYSVYSAVYAVAHALH
Hoolock hoollock	SESWAIDPVLHNLTELRHMGTFLGITIQSVPIPGFSEFRERGPQAGPPPLSRSSQRSTCNQECDNCLNATLSFNTVLRLSGERVVYSVYSAVYAVAHALH
Symphalangus syndactylus	SESWAIDPVLHNLTELRHMGTFLGITIQSVPIQGFSEFRERGPQAGPPPLSRSSQRSTCNQECDNCLNATLSFNTVLRLSGERVVYSVYSAVYAVAHALH
Hylobates lar	SESWAIDPVLHNLTELRHMGTFLGITIQSVPIPGFSEFRERGQQAGPPPLSRTSQRSTCNQECDDCLNATLSFNTVLRLSGERVVYSVYSAVYAVAHALH
Hylobates abbotti	SESWAIDPVLHNLTELRHMGTFLGITIQSVPIPGFSEFRERGQQAGPPPLSRTSQRSTCNQECDDCLNATLSFNTVLRLSGERVVYSVYSAVYAVAHALH
Hylobates agilis	SESWAIDPVLHNLTELRHMGTFLGITIQSVPIPGFSEFRERSQQAGPPPLSRTSQRSTCNQECDDCLNATLSFNTVLRLSGERVVYSVYSAVYAVAHALH
Hylobates pileatus	SESWAIDPVLHNLTELRHMGTFLGITIQSVPIPGFSEFRERGQQAGPPPLSRTSQRSTCNQECDDCLNATLSFNTVLRLSGERVVYSVYSAVYAVAHALH
Nomascus leucogenys	SESWAIDPVLHNLTELRHMGTFLGITIQSVPIPGFSEFRERGPQAGPPPLSRSSQRSTCNQECDNCLNATLSFNTVLRLSGERVVYSVYSAVYAVAHALH
Nomascus annamensis	SESWAIDPVLHNLTELRHMGTFLGITIQSVPIPGFSEFRERGPQAGPPPLSRSSQRSTCNQECDNCLNATLSFNTVLRLSGERVVYSVYSAVYAVAHALH
Pan paniscus	SESWAIDPVLHNLTELRHLGTFLGITIQSVPIPGFSEFREWGPQAGPPPLSRTSQSYTCNQECDNCLNATLSFNTILRLSGERVVYSVYSAVYAVAHALH
Pan troglodytes	SESWAIDPVLHNLTELRHLGTFLGITIQSVPIPGFSEFREWGPQAGPPPLSRTSQSYTCNQECDNCLNATLSFNTILRLSGERVVYSVYSAVYAVAHALH
Homo sapiens	SESWAIDPVLHNLTELGHLGTFLGITIQSVPIPGFSEFREWGPQAGPPPLSRTSQSYTCNQECDNCLNATLSFNTILRLSGERVVYSVYSAVYAVAHALH
Gorilla gorilla gorilla	SESWAIDPVLHNLTELRHLGTFLGITIQSVPIPGFSEFREWSPQAGPPPLSRTSQSYTCNQECDNCLNATLSFNTILRLSGERVVYSVYSAVYAVAHALH
Pongo pygmaeus	SESWAIDPVLHNLTELRHTGTFLGITIQSVPIPGFSEFRERDSQAGPPPLGKTSQRSTCNQECDNCLNATLSFNTILRLSGERVVYSVYSAVYAVAHALH

	401 … 450 … 500
Macaca arctoides	SLLGCDHGTCTKREVYPWQLLKEIWKVNFTLLDHEISFDPQGDMALHLEIVQWQWGLSQNPFQSVASYYPLQRQLKKIQDISWHTINNTIPVSMCSKRCQ
Macaca fuscata	SLLGCDHGTCTKREVYPWQLLKEIWKVNFTLLDHEISFDPQGDMALHLEIVQWQWGLSQNPFQSVASYYPLQRQLKKIQDISWHTINNTIPVSMCSKRCQ
Macaca assamensis	SLLGCDHGTCTKREVYPWQLLKEIWKVNFTLLDHEISFDPQGDMALHLEIVQWQWGLSQNPFQSVASYYPLQRQLKKIQDISWHTINNTIPVSMCSKRCQ
Cercocebus chrysogaster	SLLGCDHGTCTKTEVYPWQLLKEIWKVNFTLLDHEISFDPQGDMALHLEIVQWQWDLSQNPFQSVASYYPLQRQLKTIQDISWHTINNTIPVSMCSKRCQ
Mandrillus sphinx	SLLGCDYGTCAKKEVYPWQLLKEIWKVNFTLLDHEISFDPQGDMALHLEIVQWQWDLSQNPFQSVASYYPLQRQLKTIQDISWHTINNTIPVSMCSKRCQ
Lophocebus aterrimus	SLLGCDHGTCAKTEVYPWQLLKEIWKVNFTLLDHQIAFDPQGDMALHLEIVQWQWDLSQNPFQSVASYYPLQRQLKTIQDISWHTINNTIPVSMCSKRCQ
Papio hamadryas	SLLGCDHGTCTKTEVYPWQLLKEIWKVNFTLLDHQIAFDPQGDMALHLEIVQWQWDLSQNPFQSVASYYPLQRQLKTIQDISWHTINNTIPVSMCSKRCQ
Theropithecus gelada	SLLGCDHGTCTKTEVYPWQLLKEIWKVNFTLLDHEISFDPQGDMALHLEIVQWQWDLSQNPFQSVASYYPLQRQLKKIQDISWHTINNTIPVSMCSKRCQ
Cercopithecus mitis	SLLGCDHGACIKKEVYPWKLLKEIWKVNFTLLDHQISFDPQGDMALHLEIVQWQWGLSQNPFQSVASYYPLQRQLKIIQDISWHTINNTIPVSMCSKRCQ
Cercopithecus albogularis	SLLGCDHGACIKKEVYPWKLLKEIWKVNFTLLDHQISFDPQGDMALHLEIVQWQWGLSQNPFQSVASYYPLQRQLKIIQDISWHTINNTIPVSMCSKRCQ
Erythrocebus patas	SLLGCDHGTCTKKEVYPWQLLKEIWKVNFTLLDHQISFDPQGDMALHLEIVQWQWGLSQNPFQSVASYYPLQRQLKKIQDISWHTINNTIPVSMCSKRCQ
Chlorocebus sabaceus	SLLGCDHGTCTKKEVYPWQLLKEIWKVNFTLLDHQISFDPQGDMALHLEIVQWQWGLSQNPFQSVASYYPLQRQLKKIQDISWHTINNTIPVSMCSKRCQ
Colobus polykomos	SLLGCDHGTCTKREVYPWQLLEKIWKVNFTLLDHQLFFDPQGDMALHLEIVQWQWDQSQNPFQSVASYYPLQRQLKKTQNISWHTINNTIPVSMCSKRCQ
Presbytis melalophos	SLLGCDHGTCTKREVYPWQLLEKIWKVNFTLLDHQLFFDPQGDMALHLEIVQWQWDLSQNPFQSVASYYPLQRRLKKIQDISWHTINNTVPVSMCSKRCQ
Trachypithecus francoisi	SLLGCDYGTCTKREVYPWQLLEKIWKVNFTLLDHQLFFDPQGDMALHLEIVQWQWDLSQNPFQSVASYYPLQRQLKKIQDISWHTINNTVPVSMCSKRCQ
Semnopithecus vetulus	SLLGCDHGTCTKREVYPWQLLEKIWKVNFTLLDHQLFFDPQGDMALHLEIVQWQWDLSQNPFQSVASYYPLQRQLKKIQNISWHTINNTVPVSMCSKRCQ
Pygathrix nigripes	SLLGCDRGTCTKREVYPWQLLEKIWKVNFTLLDHQLFFDPQGDMALHLEIVQWQWDLSQNPFQSVASYYPLQRQLKIIQDISWHTINNTVPVSMCSKRCQ
Pygathrix nemaeus	SLLGCDRGTCTKREVYPWQLLEKIWKVNFTLLDHQLFFDPQGDMALHLEIVQWQWDLSQNPFQSVASYYPLQRQLKIIQDISWHTINNTVPVSMCSKRCQ
Nasalis larvatus	SLLGCDHGTCTKREVYPWQLLEKIWKVNFTLLDHQLFFDPQGDMALHLEIVQWQWDLSQNPFQSVASYYPLQRQLKTIQDISWHTINNTVPVSMCSKRCQ
Rhinopithecus bieti	SLLGCDHGTCTKREVYPWQLLEKIWKVNFTLLDHQLFFDPQGDMALHLEIVQWQWDLSQNPFQSVASYYPLQRQLKTIQDISWHTINNTVPVSMCSKRCQ
Rhinopithecus brelichi	SLLGCDHGTCTKREVYPWQLLEKIWKVNFTLLDHQLFFDPQGDMALHLEIVQWQWDLSQNPFQSVASYYPLQRQLKTIQDISWHTINNTVPVSMCSKRCQ
Rhinopithecus roxellana	SLLGCDHGTCTKREVYPWQLLEKIWKVNFTLLDHQLFFDPQGDMALHLEIVQWQWDLSQNPFQSVASYYPLQRQLKTIQDISWHTINNTVPVSMCSKRCQ
Hoolock hoollock	SLLGCDHSTCTKREVYPWQLLEEVWKVNFTLLDHQIFFDSQGDLALHLEIVQWQWDKSQNPFQSVASYYPLQRQLKNIQDISWHTINNTIPVSMCSKRCQ
Symphalangus syndactylus	SLLGCDHSTCTKREVYPWQLLEEVWKVNFTLLDHQIFFDSQGDLALHLEIVQWQWDQSQNPFQSVASYYPLQRQLKNIQDISWHTINNTIPVSMCSKRCQ
Hylobates lar	SLLDCDHSTCTKREVYPWQLLEEVWKVNFTLLDHQIFFDSQGDLALHLEIVQWQWDQSQNPFQSVASYYPLQRQLKNIQDISWHTINNTIPVSMCSKRCQ
Hylobates abbotti	SLLDCDHSTCTKREVYPWQLLEEVWKVNFTLLDHQIFFDSQGDLALHLEIVQWQWDQSQNPFQSVASYYPLQRQLKNIQDISWHTINNTIPVSMCSKRCQ
Hylobates agilis	SLLDCDHSTCTKREVYPWQLLEEVWKVNFTLLDHQIFFDSQGDLALHLEIVQWQWDQSQNPFQSVASYYPLQRQLKNIQDISWHTINNTIPVSMCSKRCQ
Hylobates pileatus	SLLDCDHSTCTKREVYPWQLLEEVWKVNFTLLDHQIFFDSQGDLALHLEFVQWQWDQSQNPFQSVASYYPLQRQLKNIQDISWHTINNTIPVSMCSKRCQ
Nomascus leucogenys	SLLNCDHSTCTKREVYPWQLLEEVWKVNFTLLDHQIFFDSQGDLALHLEIVQWQWDQSQNPFQSVASYYPLQRQLKNIQDISWHTINNTIPVSMCSKRCQ
Nomascus annamensis	SLLNCDHSTCTKREVYPWQLLEEVWKVNFTLLDRQIFFDSQGDLALHLEIVQWQWDQSQNPFQSVASYYPLQRQLKNIQDISWHTINNTIPVSMCSKRCQ
Pan paniscus	SLLGCDNSTCTKRVVYPWQLLEEIWKVNFTLLDHQIFFDPQGDVALHLEIVQWQWDRSQNPFQSVASYYPLQRQLKNIQDISWHTINNTIPVSMCSKRCQ
Pan troglodytes	SLLGCDNSTCTKRVVYPWQLLEEIWKVNFTLLDHQIFFDPQGDVALHLEIVQWQWDRSQNPFQSVASYYPLQRQLKNIQDISWHTINNTIPVSMCSKRCQ
Homo sapiens	SLLGCDKSTCTKRVVYPWQLLEEIWKVNFTLLDHQIFFDPQGDVALHLEIVQWQWDRSQNPFQSVASYYPLQRQLKNIQDISWHTVNNTIPMSMCSKRCQ
Gorilla gorilla gorilla	SLLGCDNSTCTKRVVYPWQLLEEIWKVNFTLLDHQIFFDPQGDVALHLEIVQWQWDRSQNPFQSVASYYPLQRQLKHIQDISWHTINNTIPVSMCSKRCQ
Pongo pygmaeus	SLLGCDHSTCTKRVVYPWQLLEEIWKVNFTLLDHQIFFDPQGDMALHLEIVQWQWDRSQNPFQSVASYHPLQRQLKNIQDISWHTINNTIPVSMCSKRCQ

	501 … 550 … 600
Macaca arctoides	SGQKKKPVGIHICCFECIDCLPGTFLNQTEDEFECQACPSNEWSHQSEASCFKRRLAFLEWHEAPTIVVALLAALGFLSTLAILVIFWRHFQTPMVRSAG
Macaca fuscata	SGQKKKPVGIHICCFECIDCLPGTFLNQTEDEYECQACPSNEWSHQSEASCFKRRLAFLEWHEAPTIVVALLAALGFLSTLAILVIFWRHFQTPMVRSAG
Macaca assamensis	SGQKKKPVGIHICCFECIDCLPGTFLNQTEDEYECQACPSNEWSHQSEASCFKRRLAFLEWHEAPTIVVALLAALGFLSTLAILVIFWRHFQTPMVRSAG
Cercocebus chrysogaster	SGQKKKPVGIHICCFECIDCLPGTFLNQTEDEYECQACPSNEWSHQSEASCFKRRLAFLEWHEAPTIVVALLAALGFLSTLAILVIFWRHFQTPMVRSAG
Mandrillus sphinx	SGQKKKPVGIHICCFECIDCLPGTFLNQTEDEYECQACPSNEWSHQSEASCFKRRLAFLEWHEAPTIVVALLAALGFLSTLAILVIFWRHFQTPMVRSAG
Lophocebus aterrimus	SGQKKKPVGIHICCFECIDCLPGTFLNQTEDEYECQACPSNEWSHQSEASCFKRRLAFLEWHEAPTIVVALLAALGFLSTLAILVIFWRHFQTPMVRSAG
Papio hamadryas	SGQKKKPVGIHICCFECIDCLPGTFLNQTEDEYECQACPSNEWSHQSEASCFKRRLAFLEWHEAPTIVVALLAALGFLSTLAILVIFWRHFQTPMVRSAG
Theropithecus gelada	SGQKKKPVGIHICCFECIDCLPGTFLNQTEDEYECQACPSNEWSHQSEASCFKRRLAFLEWHEAPTIVVALLAALGFLSTLAILVIFWRHFQTPMVRSAG
Cercopithecus mitis	SGQKKKPVGIHICCFECIDCLPGTFLNQTEDEYECQACPSNEWSHQSEASCFKRRLAFLEWHEAPTIVVALLAALGFLSTLAILVIFWRHFQTPMVRSAG
Cercopithecus albogularis	SGQKKKPVGIHICCFECIDCLPGTFLNQTEDEYECQACPSNEWSHQSEASCFKRRLAFLEWHEAPTIVVALLAALGFLSTLAILVIFWRHFQTPMVRSAG
Erythrocebus patas	SGQKKKPVGIHICCFECIDCLPGTFLNQTEDEYECQACPSNEWSHQSEASCFKRRLAFLEWHEAPTIVVALLAALGFLSTLAILVIFWRHFQTPMVRSAG
Chlorocebus sabaceus	SGQKKKPVGIHICCFECIDCLPGTFLNQTEDEYECQACPSNEWSHQSEASCFKRRLAFLEWHEAPTIVVALLAALGFLSTLAILVIFWRHFQTPMVRSAG
Colobus polykomos	LGQKKKPVGIHICCFECIDCLPGTFLNQTEDEYECQACPSNEWSHQREASCFKRRLAFLEWHEAPTIVVALLAALGFLSTLAILVIFWRHFQTPMVRSAG
Presbytis melalophos	SGQKKKPVGVHICCFECIDCLPGTFLNQTEDEYECQACPSNEWSHQREASCFKRQLAFPEWHEAPTIVVALLAALGFLSTLAILVIFWRHFQTPMVRSAG
Trachypithecus francoisi	LGQKKKPVGIHICCFECIDCLPGTFLNQTEDEYECQACPSNEWSHQREASCFKRQLAFLEWHEAPTIVVALLAALGFLSTLAILVIFWRHFQTPMVRSAG
Semnopithecus vetulus	LGQKKKPVGIHICCFECIDCLPGTFLNQTEDEYECQACPSNEWSHQREASCFKRQLAFLEWHEAPTIVVALLAALGFLSTLAILVIFWRHFQTPMVRSAG
Pygathrix nigripes	SGQKKKPVGVHICCFECIDCLPGTFLNQTEDAYECQACPSNEWSHQREASCFKRQLAFLEWHEAPTIVVALLAALGFLSTLAILVIFWRHFQTPMVRSAG
Pygathrix nemaeus	SGQKKKPVGVHICCFECIDCLPGTFLNQTEDAYECQACPSNEWSHQREASCFKRQLAFLEWHEAPTIVVALLAALGFLSTLAILVIFWRHFQTPMVRSAG
Nasalis larvatus	SGQKKKPVGIHICCFECIDCLPGTFLNQTEDEYECQACPSNEWSHQREASCFKRQLAFPEWHEAPTIVVALLAALGFLSTLAILVIFWRHFQTPMVRSAG
Rhinopithecus bieti	SGQKKKPVGVHICCFECIDCLPGTFLNQTEDEYECQACPSNEWSHQREASCFKRQLAFLEWHEAPTIVVALLAALGFLSTLAILVIFWRHFQTPMVRSAG
Rhinopithecus brelichi	SGQKKKPVGVHICCFECIDCLPGTFLNQTEDEYECQACPSNEWSHQREASCFKRQLAFLEWHEAPTIVVALLAALGFLSTLAILVIFWRHFQTPMVRSAG
Rhinopithecus roxellana	SGQKKKPVGVHICCFECIDCLPGTFLNQTEDEYECQACPSNEWSHQREASCFKRQLAFLEWHEAPTIVVALLAALGFLSTLAILVIFWRHFQTPMVRSAG
Hoolock hoollock	SGQKKKPVGIHVCCFECIDCLPGTFLNHTEDEYECQACPSNEWSHQSETSCFKRQLVFLEWHEAPTIAVALLAALGFLSTLAILVVFWRHFQTPMVRSAG
Symphalangus syndactylus	SGQKKKPVGIHVCCFECIDCLPGTFLNHTEDEYECQACPSNEWSHQSETSCFKRQLVFLEWHEAPTIAVALLAALGFLSTLAILVVFWRHFQTPMVRSAG
Hylobates lar	SGQKKKPVGIHVCCFECIDCLPGTFLNHTEDEYECQACPNNEWSHQSETSCFKRQLVFLEWHEAPTIAVALLAALGFLSTLAILVVFWRHFQTPMVRSAG
Hylobates abbotti	SGQKKKPVGIHVCCFECIDCLPGTFLNHTEDEYECQACPNNEWSHQSETSCFKRQLVFLEWHEAPTIAVALLAALGFLSTLAILVVFWRHFQTPMVRSAG
Hylobates agilis	SGQKKKPVGIHVCCFECIDCLPGTFLNHTEDEYECQACPNNEWSHQSETSCFKRQLVFLEWHEAPTIAVALLAALGFLSTLAILVVFWRHFQTPMVRSAG
Hylobates pileatus	SGQKKKPVGIHVCCFECIDCLPGTFLNHTEDEYECQACPNNEWSHQSETSCFKRQLVFLEWHEAPTIAVALLAALGFLSTLAILVVFWRHFQTPMVRSAG
Nomascus leucogenys	SGQKKKPVGIHVCCFECIDCLPGTFLNHTEDEYECQACPNNEWSHQSETSCFKRQLVFLEWHEAPTIAVALLAALGFLSTLAILVVFWRHFQTPMVRSAG
Nomascus annamensis	SGQKKKPVGIHVCCFECIDCLPGTFLNHTEDEYECQACPNNEWSHQSETSCFKRQLVFLEWHEAPTIAVALLAALGFLSTLAILVVFWRHFQTPMVRSAG
Pan paniscus	SGQKKKPVGIHVCCFECIDCLPGTFLNHTEDEYECQACPNNEWSYQSETSCFKRQLVFLEWHEAPTIAVALLAALGFLSTLAILVIFWRHFQTPIVRSAG
Pan troglodytes	SGQKKKPVGIHVCCFECIDCLPGTFLNHTEDEYECQACPNNEWSYQSETSCFKRQLVFLEWHEAPTIAVALLAALGFLSTLAILVIFWRHFQTPIVRSAG
Homo sapiens	SGQKKKPVGIHVCCFECIDCLPGTFLNHTEDEYECQACPNNEWSYQSETSCFKRQLVFLEWHEAPTIAVALLAALGFLSTLAILVIFWRHFQTPIVRSAG
Gorilla gorilla gorilla	SGQKKKPVGIHVCCFECIDCLPGTFLNHTEDEYECQACPNNEWSYQSETSCFKRQLVFLEWHEAPTIAVALLAALGFLSTLAILVIFWRHFQTPIVRSAG
Pongo pygmaeus	SGQKKKPVGIHVCCFECIDCLPGTFLNHTEDEYECQACPSNEWSYQSETSCFKRQLAFLEWHEAPTIAVALLAALGFLSTLAILVIFWRHFQTPMVRSAG

Species	601 — 650 — 700
Macaca arctoides	GPMCFLMLTLLLVAYMVVPVYVGPPKVSTCFCRQALFPLCFTICISCIAVRSFQIVCVFKMASRFPRAYSYWVRYQGPYVSMAFITVLKMVTVVIGMLAT
Macaca fuscata	GPMCFLMLTLLLVAYMVVPVYVGPPKVSTCFCRQALFPLCFTICISCIAVRSFQIVCVFKMASRFPRAYSYWVRYQGPYVSMAFITVLKMVTVVIGMLVT
Macaca assamensis	GPMCFLMLTLLLVAYMVVPVYVGPPKVSTCFCRQALFPLCFTICISCIAVRSFQIVCVFKMASRFPRAYSYWVRYQGPYVSMAFITVLKMVTVVIGMLVT
Cercocebus chrysogaster	GPMCFLMLTLLLVAYMVVPVYVGPPKVSTCFCRQALFPLCFTICISCIAVRSFQIVCVFKMASRFPRAYSYWVRYQGPYVSMAFITVLKMVTVVIGMLAT
Mandrillus sphinx	GPMCFLMLTLLLVAYMVVPVYVGPPKVSTCFCRQALFPLCFTICISCIAVRSFQIVCVFKMASRFPRAYSYWVRYQGPYVSMAFITVLKMVTVVIGMLAT
Lophocebus aterrimus	GPMCFLMLTLLLVAYMVVPVYVGPPKVSTCFCRQALFPLCFTICISCIAVRSFQIVCVFKMASRFPRAYSYWVRYQGPYVSMAFITVLKMVTVVIGMLAT
Papio hamadryas	GPMCFLMLTLLLVAYMVVPVYVGPPKVSTCFCRQALFPLCFTICISCIAVRSFQIVCVFKMASRFPRAYSYWVRYQGPYVSMAFITVLKMVTVVIGMLAT
Theropithecus gelada	GPMCFLMLTLLLVAYMVVPVYVGPPKVSTCFCRQALFPLCFTICISCIAVRSFQIVCVFKMASRFPRAYSYWVRYQGPYVSMAFITVLKMVTVVIGMLAT
Cercopithecus mitis	GPMCFLMLTLLLVAYMVVPVYVGPPKVSTCFCRQALFPLCFTICISCIAVRSFQIVCVFKMASRFPRAYSYWVRYQGPYVSMAFITVLKMVTVVIGMLAT
Cercopithecus albogularis	GPMCFLMLTLLLVAYMVVPVYVGPPKVSTCFCRQALFPLCFTICISCIAVRSFQIVCVFKMASRFPRAYSYWVRYQGPYVSMAFITVLKMVTVVIGMLAT
Erythrocebus patas	GPMCFLMLTLLLVAYMVVPVYVGPPKVSTCFCRQALFPLCFTICISCIAVRSFQIVCVFKMASRFPRAYSYWVRYQGPYVSMAFITVLKMVTVVIGMLAT
Chlorocebus sabaceus	GPMCFLMLTLLLVAYMVVPVYVGPPKVSTCFCRQALFPLCFTICISCIAVRSFQIVCVFKMASRFPRAYSYWVRYQGPYVSMAFITVLKMVTVVIGMLAT
Colobus polykomos	GPMCFLMLTLLLVAYVVVPVYVGPPKVSTCLCRQVLFPLCFTICISCITVRSFQIVCVFKMASRFPRAYSYWVRYQGPYVSMAFIMVLKMVTVVIGILAT
Presbytis melalophos	GPMCFLMLTLLLVAYVVVPVYVGPPKVSTCLCRQVLFPLCFTICISCITVRSFQIVCVFKMASRFPRAYSYWVRYQGPYVSMAFIMVLKMVTMVIGILAT
Trachypithecus francoisi	GPMCFLMLTLLLVAYVVVPVYVGPPKVSTCLCRQVLFPLCFTICISCITVRSFQIVCVFKMASRFPRAYSYWVRYQGPYVSMAFIMVLKMVTMVIGILVT
Semnopithecus vetulus	GPMCFLMLTLLLVAYVVVPVYVGPPKVSTCLCRQVLFPLCFTTCISCITVRSFQIVCVFKMASRFPRAYSYWVRYQGPYVSMAFIMVLKMVTMVIGILVT
Pygathrix nigripes	GSMCFLMLTLLLVAYVVVPVYVGPPKVSTCLCRQVLFPLCFTICISCITVRSFQIVCVFKMASRFPRAYSYWVRYQGPYVSMAFIMVLKMVTVVISILAT
Pygathrix nemaeus	GSMCFLMLTLLLVAYVVVPVYVGPPKVSTCLCRQVLFPLCFTICISCITVRSFQIVCVFKMASRFPRAYSYWVRYQGPYVSMAFIMVLKMVTVVISILAT
Nasalis larvatus	GPMCFLMLTLLLVAYVVVPVYVGPPKVSTCLCRQVLFPLCFTICISCITVRSFQIVCVFKMASRFPRAYSYWVRYQGPYVSMAFIMVLKMVTMVIGILVT
Rhinopithecus bieti	GPMCFLMLTLLLVAYVVVPVYVGPPKVSTCLCRQVLFPLCFTICISCITVRSFQIVCVFKMASRFPRAYSYWVRYQGPYVSMAFIMVLKMVTMVIGILAT
Rhinopithecus brelichi	GPMCFLMLTLLLVAYVVVPVYVGPPKVSTCLCRQVLFPLCFTICISCITVRSFQIVCVFKMASRFPRAYSYWVRYQGPYVSMAFIMVLKMVTMVIGILAT
Rhinopithecus roxellana	GPMCFLMLTLLLVAYVVVPVYVGPPKVSTCLCRQVLFPLCFTICISCITVRSFQIVCVFKMASRFPRAYSYWVRYQGPYVSMAFIMVLKMVTMVIGILAT
Hoolock hoollock	GPMCFLMLTLLLVAYMVVPVYMGPPKVSTCLCRQALFPLCFTICISCIAVRSFQIVCAFKMASRFPRAYSYWVRYQGPYVSMAFITVLKMVIVVIGMLAT
Symphalangus syndactylus	GPMCFLMLTLLLVAYMVVPVYMGPPKVSTCLCRQALYPLCFTICISCIAVRSFQIVCAFKMASRFPRAYSYWVRYQGPYVSMAFITVLKMVIVVIGMLAT
Hylobates lar	GPMCFLMLTLLLVAYMVVPVYMGPPKVSTCLCRQALFPLCFTICISCITVRSFQIVCAFKMASRFPRAYSYWVRYQGPYVSMAFITVLKMVIVVIGMLAT
Hylobates abbotti	GPMCFLMLTLLLVAYMVVPVYMGPPKVSTCLCRQALFPLCFTICISCITVRSFQIVCAFKMASRFPRAYSYWVRYQGPYVSMAFITVLKMVIVVIGMLAT
Hylobates agilis	GPMCFLMLTLLLVAYMVVPVYMGPPKVSTCLCRQALFPLCFTICISCITVRSFQIVCAFKMASRFPRAYSYWVRYQGPYVSMAFITVLKMVIVVIGMLAT
Hylobates pileatus	GPMCFLMLTLLLVAYMVVPVYMGPPKVSTCLCRQALFPLCFTICISCITVRSFQIVCAFKMASRFPRAYSYWVRYQGPYVSMAFITVLKMVIVVIGMLAT
Nomascus leucogenys	GPMCFLMLTLLLVAYMVVPVYMGPPKVSTCLCRQALFPLCFTICISCIAVRSFQIVCAFKMASRFPRAYSYWVRYQGPYVSMAFITVLKMVIVVIGMLAT
Nomascus annamensis	GPMCFLMLTLLLVAYMVVPVYMGPPKVSTCLCRQALFPLCFTICISCIAVRSFQIVCAFKMASRFPRAYSYWVRYQGPYVSMAFITVLKMVIVVIGMLAT
Pan paniscus	GPMCFLMLTLLLVAYMVVPVYVGPPKVSTCLCRQALFPLCFTICISCIAVRSFQIVCAFKMASRFPRAYSYWVRYQGPYVSMTFITVLKMVIVVIGMLAT
Pan troglodytes	GPMCFLMLTLLLVAYMVVPVYVGPPKVSTCLCRQALFPLCFTICISCIAVRSFQIVCAFKMASRFPRAYSYWVRYQGPYVSMAFITVLKMVIVVIGMLAT
Homo sapiens	GPMCFLMLTLLLVAYMVVPVYVGPPKVSTCLCRQALFPLCFTICISCIAVRSFQIVCAFKMASRFPRAYSYWVRYQGPYVSMAFITVLKMVIVVIGMLAT
Gorilla gorilla gorilla	GPMCFLMLTLLLVAYMVVPVYVGPPKVSTCLCRQALFPLCFTICISCIAVRSFQIVCAFKMASRFPRAYSYWVRYQGPYVSMAFITVLKMVIVVIGMLAT
Pongo pygmaeus	GPMCFLMLTLLLVAYMVVPVYVGPPKVSTCLCRQALFPLCFTICISCIAVRSFQIICAFKMASRFPRAYSYWVRYQGPYVSMAFITVLKMVIVVIGMLAT

Species	701 — 750 — 800
Macaca arctoides	GLNPTTRIDPDDDPKIMIVSCNPNYRNSLFFNTGLDLLLSVVGFSFAYMGKELPTNYNEAKFITLSMTFYFTSSVSLCTFMSAYNGVLVTIMDLLVTVLNL
Macaca fuscata	GLNPTTRIDPDDDPKIMIVSCNPNYRNSLFFNTGLDLLLSVVGFSFAYMGKELPTNYNEAKFITLSMTFYFTSSVSLCTFMSAYNGVLVTIMDLLVTVLNL
Macaca assamensis	GLNPTTRIDPDDDPKIMIVSCNPNYRNSLFFNTGLDLLLSVVGFSFAYMGKELPTNYNEAKFITLSMTFYFTSSVSLCTFMSAYNGVLVTIMDLLVTVLNL
Cercocebus chrysogaster	GLNPTTRIDPDDDPKIMIVSCNPNYRNSLFFNTSLDLLLSVVGFSFAYMGKELPTNYNEAKFITLSMTFYFTSSVSLCTFMSAYNGVLVTIMDLLVTVLNL
Mandrillus sphinx	GLNPTTRIDPDDDPKIMIVSCNPNYRNSLFFNTSLDLLLSVVGFSFAYMGKELPTNYNEAKFITLSMTFYFTSSVSLCTFMSVYNGVLVTIMDLLVTVLNL
Lophocebus aterrimus	GLNPTTRIDPDDDPKIMIVSCNPNYRNSLFFNTSLDLLLSVVGFSFAYMGKELPTNYNEAKFITLSMTFYFTSSVSLCTFMSAYNGVLVTIMDLLVTVLNL
Papio hamadryas	GLNPTTRIDPDDDPKIMIVSCNPNYRNSLFFNTGLDLLLSVVGFSFAYMGKELPTNYNEAKFITLSMTFYFTSSVSLCTFMSAYNGVLVTIMDLLVTVLNL
Theropithecus gelada	GLNPTTRIDPDDDPKIMIVSCNPNYRNSLFFNTGLDLLLSVVGFSFAYMGKELPTNYNEAKFITLSMTFYFTSSVSLCTFMSAYNGVLVTIMDLLVTVLNL
Cercopithecus mitis	GLNPTTRIDPDDDPKIMIVSCNPNYRNSLFFNTSLDLLLSVVGFSFAYMGKELPTNYNEAKFITLSMTFYFTSSVSLCTFMSAYNGVLVTIMDLLVTVLNL
Cercopithecus albogularis	GLNPTTRIDPDDDPKIMIVSCNPNYRNSLFFNTSLDLLLSVVGFSFAYMGKELPTNYNEAKFITLSMTFYFTSSVSLCTFMSAYNGVLVTIMDLLVTVLNL
Erythrocebus patas	GLNPTTRIDPDDDPKIMIVSCNPNYRNSLFFNTSLDLLLSVVGFSFAYMGKELPTNYNEAKFITLSMTFYFTSSVSLCTFMSAYNGVLVTIMDLLVTVLNL
Chlorocebus sabaceus	GLNPTTRIDPDDDPKIMIVSCNPNYRNSLFFNTSLDLLLSVVGFSFAYMGKELPTNYNEAKFITLSMTFYFTSSVSLCTFMSAYNGVLVTIMDLLVTVLNL
Colobus polykomos	GLNPTTRIDPDDDPKIMIISCNPNYRNSLLFNTSLDLLLSVLGFSFAYMGKELPTNYNEAKFITLSMTFYFTSSVSLCTFMSAYNGVLVTIMDLLVTVLNL
Presbytis melalophos	GLNPTTRVDPDDDPKIMIVSCNPNYRNSLLFNTSLDLLLSVLGFSFAYMGKELPTNYNEAKFITLSMAFYFTSSVSLCTFMSAYNGVLVTIMDLLVTVLNL
Trachypithecus francoisi	GLNPTTRIDPDDDPKIMIVSCNPNYRNSLLFNTSLDLLLSVLGFSFAYMGKELPTNYNEAKFITLSMTFYFTSSVSLCTFMSAYNGVLVTIMDLLVTVLNL
Semnopithecus vetulus	GLNPTTRIDPDDDPKIMIVSCNPNYRNSLLFNTSLDLLLSVLGFSFAYMGKELPTNYNEAKFITLSMTFYFTSSVSLCTFMSAYNGVLVTIMDLLVTVLNL
Pygathrix nigripes	GLNPTTRVDPDDDPKIMIVSCNPNYRNSLLFNTSLDLLLSVLGFSFAYMGKELPTNYNEAKFITLSMTFYFTSSISLCTFMSAYNGVLVTIMDLLVTVLNL
Pygathrix nemaeus	GLNPTTRVDPDDDPKIMIVSCNPNYRNSLLFNTSLDLLLSVLGFSFAYMGKELPTNYNEAKFITLSMTFYFTSSVSLCTFMSAYNGVLVTIMDLLVTVLNL
Nasalis larvatus	GLNPTTRVDPDDDPKIMIVSCNPNYRNSLLFNTSLDLLLSVLGFSFAYVGKELPTNYNEAKFITLSMTFYFTSSVSLCTFMSAYNGVLVTIMDLLVTVLNL
Rhinopithecus bieti	GLNPTTHVDPDDDPKIMIVSCNPNYRNSLLFNTSLDLLLSVLGFSFAYVGKELPTNYNEAKFITLSMTFYFTSSVSLCTFMSAYNGVLVTIMDLLVTVLNL
Rhinopithecus brelichi	GLNPTTHVDPDDDPKIMIVSCNPNYRNSLLFNTSLDLLLSVLGFSFAYVGKELPTNYNEAKFITLSMTFYFTSSVSLCTFMSAYNGVLVTIMDLLVTVLNL
Rhinopithecus roxellana	GLNPTTHVDPDDDPKIMIVSCNPNYRNSLLFNTSLDLLLSVLGFSFAYVGKELPTNYNEAKFITLSMTFYFTSSVSLCTFMSAYNGVLVTIMDLLVTVLNL
Hoolock hoollock	GLSPTTRTDPDDDPKITIVSCNPNYRNSLLFNTSLDLLLSVVGFSFAYMGRELPTNYNEAKFITLSMTFYFTSSVSLCTFMSAYNGVLVTIVDLLVTVLNL
Symphalangus syndactylus	GLSPTTRTDPDDDPKITIVSCNPNYRNSLLFNTSLDLLLSVVGFSFAYMGRELPTNYNEAKFITLSMTFYFTSSVSLCTFMSAYNGVLVTIVDLLVTVLNL
Hylobates lar	GLSPTTRTDPDDDPKITIVSCNPNYRNSLLFNTSLDLLLSVVGFSFAYMGRELPTNYNEAKFITLSMTFYFTSSVSLCTFMSAYNGVLVTIVDLLVTVLNL
Hylobates abbotti	GLSPTTRTDPDDDPKITIVSCNPNYRNSLLFNTSLDLLLSVVGFSFAYMGRELPTNYNEAKFITLSMTFYFTSSVSLCTFMSAYNGVLVTIVDLLVTVLNL
Hylobates agilis	GLSPTTRTDPDDDPKITIVSCNPNYRNSLLFNTSLDLLLSVVGFSFAYMGRELPTNYNEAKFITLSMTFYFTSSVSLCTFMSAYNGVLVTIVDLLVTVLNL
Hylobates pileatus	GLSPTTRTDPDDDPKITIVSCNPNYRNSLLFNTSLDLLLSVVGFSFAYMGRELPTNYNEAKFITLSMTFYFTSSVSLCTFMSAYNGVLVTIVDLLVTVLNL
Nomascus leucogenys	GLSPTTRTDPDDDPKITIVSCNPNYRNSLLFNTSLDLLLSVVGFSFAYMGRELPTNYNEAKFITLSMTFYFTSSVSLCTFMSAYNGVLVTIVDLLVTVLNL
Nomascus annamensis	GLSPTTRTDPDDDPKITIVSCNPNYRNSLLFNTSLDLLLSVVGFSFAYMGRELPTNYNEAKFITLSMTFYFTSSVSLCTFMSAYNGVLVTIVDLLVTVLNL
Pan paniscus	GLSPTTRTDPDDDPKITIVSCNPNYRNSLLFNTSLDLLLSVVGFSFAYMGKELPTNYNEAKFITLSMTFYFTSSVSLCTFMSAYSGVLVTIVDLLVTVLNL
Pan troglodytes	GLSPTTRTDPDDDPKITIVSCNPNYRNSLLFNTSLDLLLSVVGFSFAYMGKELPTNYNEAKFITLSMTFYFTSSVSLCTFMSAYSGVLVTIVDLLVTVLNL
Homo sapiens	GLSPTTRTDPDDDPKITIVSCNPNYRNSLLFNTSLDLLLSVVGFSFAYMGKELPTNYNEAKFITLSMTFYFTSSVSLCTFMSAYSGVLVTIVDLLVTVLNL
Gorilla gorilla gorilla	GLSPTTRTDPDDDPKITIVSCNPNYRNSLLFNTSLDLLLSVVGFSFAYMGKELPTNYNEAKFITLSMTFYFTSSVSLCTFMSAYSGVLVTIVDLLVTVLNL
Pongo pygmaeus	GLNPTTRTDPDDDPKIMIVSCNPNYRNSLLFNTSLDLLLSVVGFSFANMGKELPTNYNEAKFITLSMTFYFTSSISLCTFMSAYSGVLVTIVDLLVTVLNL

	801 839
Macaca arctoides	LAISLGYFGPKCYMILFYPERNTPAYFNSMIQGYTMRRD
Macaca fuscata	LAISLGYFGPKCYMILFYPERNTPAYFNSMIQGYTMRRD
Macaca assamensis	LAISLGYFGPKCYMILFYPERNTPAYFNSMIQGYTMRRD
Cercocebus chrysogaster	LAISLGYFGPKCYMILFYPERNTPAYFNSMIQGYTMRRD
Mandrillus sphinx	LAISLGYFGPKCYMILFYPERNTPAYFNSMIQGYTMRRD
Lophocebus aterrimus	LAISLGYFGPKCYMILFYPERNTPAYFNSMIQGYTMRRD
Papio hamadryas	LAISLGYFGPKCYMILFYPERNTPAYFNSMIQGYTMRRD
Theropithecus gelada	LAISLGYFGPKCYMILFYPERNTPAYFNSMIQGYTMRRD
Cercopithecus mitis	LAISLGYFGPKCYMILFYPERNTPAYFNSMIQGYTMRRD
Cercopithecus albogularis	LAISLGYFGPKCYMILFYPERNTPAYFNSMIQGYTMRRD
Erythrocebus patas	LAISLGYFGPKCYMILFYPERNTPAYFNSMIQGYTMRRD
Chlorocebus sabaceus	LAISLGYFGPKCYMILFYPERNTPAYFNSMIQGYTMRRD
Colobus polykomos	LAISLGYFGPKCYMILFYPERNTPAYFNSMIQGYTMRRD
Presbytis melalophos	LAISLGYFGPKCYMILFYPERNTPAYFNSMIQGYTMRRD
Trachypithecus francoisi	LAISLGYFGPKCYMILFYPERNTPAYFNSMIQGYTMRRD
Semnopithecus vetulus	LAISLGYFGPKCYMILFYPERNTPAYFNSMIQGYTMRRD
Pygathrix nigripes	LAISLGYFGPKCYMILFYPERNTPAYFNSMIQGYTMRRD
Pygathrix nemaeus	LAISLGYFGPKCYMILFYPERNTPAYFNSMIQGYTMRRD
Nasalis larvatus	LAISLGYFGPKCYMILFYPERNTPAYFNSMIQGYTMRRD
Rhinopithecus bieti	LAISLGYFGPKCYMILFYPERNTPAYFNSMIQGYTMRRD
Rhinopithecus brelichi	LAISLGYFGPKCYMILFYPERNTPAYFNSMIQGYTMRRD
Rhinopithecus roxellana	LAISLGYFGPKCYMILFYPERNTPAYFNSMIQGYTMRRD
Hoolock hoollock	LAISLGYFGPKCYMILFYPERNTPAYFNSMIQGYTMRRD
Symphalangus syndactylus	LAISLGYFGPKCYMILFYPERNTPAYFNSMIQGYTMRRD
Hylobates lar	LAISLGYFGPKCYMILFYPERNTPAYFNSMIQGYTMRRD
Hylobates abbotti	LAISLGYFGPKCYMILFYPERNTPAYFNSMIQGYTMRRD
Hylobates agilis	LAISLGYFGPKCYMILFYPERNTPAYFNSMIQGYTMRRD
Hylobates pileatus	LAISLGYFGPKCYMILFYPERNTPAYFNSMIQGYTMRRD
Nomascus leucogenys	LAISLGYFGPKCYMILFYPERNTPAYFNSMIQGYTMRRD
Nomascus annamensis	LAISLGYFGPKCYMILFYPERNTPAYFNSMIQGYTMRRD
Pan paniscus	LAISLGYFGPKCYMILFYPERNTPAYFNSMIQGYTMRRD
Pan troglodytes	LAISLGYFGPKCYMILFYPERNTSAYFNSMIQGYTMRRD
Homo sapiens	LAISLGYFGPKCYMILFYPERNTPAYFNSMIQGYTMRRD
Gorilla gorilla gorilla	LAISLGYFGPKCYMILFYPERNTPAYFNSMIQGYTMRRD
Pongo pygmaeus	LAISLGYFGPKCYMILFYPERNTPAYFNSVIQGYTMTRD
