# Supplementary material for: Differentiated adaptive evolution, episodic relaxation of selective constraints, and pseudogenization of umami and sweet taste genes TAS1Rs in catarrhine primates
Source: Front Zool. 2014 Oct 29;11:79. doi: 10.1186/s12983-014-0079-4 (PMC4226867; doi:10.1186/s12983-014-0079-4)
Supplement: Additional file 5: — Alignment of TAS1R3 amino acid sequences of 35 catarrhine primates. Variant residues are marked with red. [file 12983_2014_79_MOESM5_ESM.pdf]

Additional file 5. Alignment of TAS1R3 amino acid sequences of 35 catarrhine primates. Variant residues are marked with red.

	1 … 50 … 100
Theropithecus gelada	MLRPAVLGLSLWALLHLGTGAPLCLSQQLRMKGDYVLGGLFPLGEAEEAGLSSRTRPSSPVCTRLSSNGLLWALAMKMAVEEINNRSDLLPGLRLGHDLF
Papio hamadryas	MLRPAVLGLSLWALLHLGTGAPLCLSQQLRMKGDYVLGGLFPLGEAEEAGLGSRTRPSSPVCTRFSSNGLLWALAMKMAVEEINNRSDLLPGLRLGHDLF
Cercocebus chrysogaster	MLRPAVLGLSLWAVLHLGTGAPLCLSQQLRMKGDYVLGGLFPLGEAEEAGLGSRTRPSSPVCTRFSSNGLLWALAMKMAVEEINNRSDLLPGLRLGHDLF
Mandrillus sphinx	MLRPAVLGLSLWAVLHLGTGAPLCLSQQLRMKGDYVLGGLFPLGEAEEAGLGSRTRPSSPVCTRFSSNGLLWALAMKMAVEEINNRSDLLPGLRLGHDLF
Macaca assamensis	MLCPAVLGLSLWALLHLGTGAPLCLSQQLRMKGDYVLGGLFPLGEAEEAGLGSRTRPSSPVCTRFSSNGLLWALAMKMAVEEINNRSDLLPGLRLGHDLF
Macaca arctoides	MLCPAVLGLSLWALLHLGTGAPLCLSQQLRMKGDYVLGGLFPLGEAEEAGLGSRTRPSSPVCTRFSSNGLLWALAMKMAVEEINNRSDLLPGLRLGHDLF
Macaca fuscata	MLRPAVLGLSLWALLHLGTGAPLCLSQQLRMKGDYVLGGLFPLGEAEEAGLGSRTRPSSPVCTRFSSNGLLWALAMKMAVEEINNRSDLLPGLRLGHDLF
Cercopithecus mitis	MLRPAVLGLSLWALLHLGTGAPLCLSQQLRMKGDYVLGGLFPLGEAEEAGLGSRTRPSSPVCTRFSSNGLLWALAMKMAVEEINNRSDLLPGLRLGHDLF
Cercopithecus albogularis	MLRPAVLGLSLWALLHLGTGAPLCLSQQLRMKGDYVLGGLFPLGEAEEAGLGSRTRPSSPVCTRFSSNGLLWALAMKMAVEEINNRSDLLPGLRLGHDLF
Erythrocebus patas	MLRPAVLGLSLWALLHLGTGAPLCLSQQLRMKGDYVLGGLFPLGEAEEVGLGSRTRPSSPVCTRFSSNGLLWALAMKMAVEEINNRSDLLPGLRLGHDLF
Chlorocebus sabaceus	MLHPAVLGLSLWALLHLGTGAPLCLSQQLRMKGDYVLGGLFPLGEAEEAGLGSRTRPSSPVCTRFSSNGLLWALAMKMAVEEINNRSDLLPGLRLGHDLF
Nasalis larvatus	MLGPAVLGLSLWALLHLGTGAPLCLSQQLRMKGDYVLGGLFPLSEAGEAGLGSRTQPSSPVCTRFSSNGLLWALAMKMAVEEINNRSDLLPGLRLGYDLF
Pygathrix nemaeus	MLGPAVLGLSLWALLHLGTGAPLCLSQQLRMKGDYVLGGLFPLSEAGEAGLGSRTQPSSPVCTRFSSDGLLWALAMKMAVEEINNRSDLLPGLRLGYDLF
Pygathrix nigripes	MLGPAVLGLSLWALLHLGTGAPLCLSQQLRMKGDYVLGGLFPLSEAGEAGLGSRTQPSSPVCTRFSLDGLLWALAMKMAVEEINNRSDLLPGLRLGYDLF
Rhinopithecus bieti	MLGPAVLGLSLWAVLHLGTGAPLCLSQQLRMKGDYVLGGLFPLSEAGEAGLGSRTQPSSPVCTRFSSNGLLWALAMKMAVEEINNRSDLLPGLHLGYDLF
Rhinopithecus brelichi	MLGPAVLGLSLWAVLHLGTGAPLCLSQQLRMKGDYVLGGLFPLSEAGEAGLGSRTQPSSPVCTRFSSNGLLWALAMKMAVEEINNRSDLLPGLHLGYDLF
Rhinopithecus roxellana	MLGPAVLGLSLWAVLHLGTGAPLCLSQQLRMKGDYVLGGLFPLSEAGEAGLGSRTQPSSPVCTRFSSNGLLWALAMKMAVEEINNRSDLLPGLRLGYDLF
Trachypithecus francoisi	MLGPAVLGLSLWALLHLGTGAPLCLSQQLRMKGDYVLGGLFPLSEAGEAGLSSRTRPSSPVCTRFSSNGLLWALAMKMAVEEINNRSDLLPGLRLGYDLF
Semnopithecus vector	MLGPAVLGLSLWALLHLGTGAPLCLSQQLRMKGDYVLGGLFPLSEAGEAGLSSRTRPSSPVCTRFSSNGLLWALAMKMAVEEINNRSDLLPGLRLGYDLF
Presbytis melalophos	MLCPAVLGLSLWALLHLGTGAPLCLSQQLRMKGDYVLGGLFPLSEAGEAGLGSRTRPSSPVCTRFSSNGLLWALAMKMAVEEINNRSDLLPGLRLGYDLF
Colobus polykomos	MLGPAVLGLSLWALLHLGTGAPLCLSQQLRMNGDYVLGGLFPLSEAGEAGLGSRTRPSSPVCTRFSSNGLLWALAMKMAVEEINNRSDLLPGLRLGYDLF
Hoolock hoolock	MLGPAVLGLSLWALLHPGMGAPLCLSQQLRMKGDYVLGGLFPLGEAEEAGIHSRTRPSSPVCTRFSSNGLLWALAMKMAVEEINNKSDLLPGLRLGYDLF
Symphalangus syndactylus	MLGPAVLGLSLWALLHPGTGAPLCLSQQLRMKGDYMLGGLFPLGEAEEAGIRSRTRPSSPVCTRFSSNGLLWALAMKMAVEEINNKSDLLPGLRLGYDLF
Hylobates pileatus	MLGPAVLGLSLWALLHPGTGAPLCLSQQLRMKGDYMLGGLFPLGEAEEAGIRSRTRPSSPVCTRFSSNGLLWALAMKMAVEEINNKSDLLPGLRLGYDLF
Hylobates lar	MLGPAVLGLSLWALLHPGTGAPLCLSQQLRMKGDYMLGGLFPLGEAEEAGIRSQTRPSSPVCTRFSSNGLLWALAMKMAVEEINNKSDLLPGLRLGYDLF
Hylobates abbotti	MLGPAVLGLSLWALLHPGTGAPLCLSQQLRMKGDYMLGGLFPLGEAEEAGIRSRTRPSSPVCTRFSSNGLLWALAMKMAVEEINNKSDLLPGLRLGYDLF
Hylobates agilis	MLGPAVLGLSLWALLHPGTGAPLCLSQQLRMKGDYMLGGLFPLGEAEEAGIRSRTRPSSPVCTRFSSNGLLWALAMKMAVEEINNKSDLLPGLRLGYDLF
Nomascus annamensis	MLGPAVLGLSLWALLHPGTGAPLCLSQQLRMKGDYVLGGLFPLGEAEEAGIRSRTRPSSPVCTRFSSNGLLWALAMKMAVEEINNKSDLLPGLRLGYDLF
Nomascus leucogenys	MLGPAVLGLSLWALLHPGTGAPLCLSQQLRMKGDYVLGGLFPLGEAEEAGIRSRTRPSSPVCTRFSSNGLLWALAMKMAVEEINNKSDLLPGLRLGYDLF
Pan troglodytes	MLGPAVLGLSLWALLHPGTGAPLCLSQQLRMKGDYVLGGLFPLGEAEEAGLRSRTRPSSPVCTRFSSNGLLWALAMKMAVEEINNKSDLLPGLRLGYDLF
Homo sapiens	MLGPAVLGLSLWALLHPGTGAPLCLSQQLRMKGDYVLGGLFPLGEAEEAGLRSRTRPSSPVCTRFSSNGLLWALAMKMAVEEINNKSDLLPGLRLGYDLF
Gorilla gorilla gorilla	MLGPAVLGLSLWALLQPGAGAPLCLSQQLRMKGDYMLGGLFPLGEAEEAGFRSRTRPSSPVCTRFSSNGLLWALAMKMAVEEINNKSDLLPGLRLGYDLF
Pongo abelii	MLGPAVLGLSLWALLHSGTGAPLCLSQQLRMKGDYVLGGLFPLGEAEEAGLRSRTRPSSPVCTRFSSNGLLWALAMKMAVEEINNKSDLLPGLRLGYDLF
Pongo pygmaeus	MLGPAVLGLSLWALLHSGTGAPLCLSQQLRMKGDYVLGGLFPLGEAEEAGLRSRTRPSSPVCTRFSSNGLLWALAMKMAVEEINNKSDLLPGLRLGYDLF
Lophocebus aterrimus	MLRPAVLGLSLWALLHLGTGAPLCLSQQLRMKGDYMLGGLFPLGEAEEAGLGSRTQPSSPVCTRFSSNGLLWALAMKMAVEEINNRSDLLPGLRLGHDLF

Species	101 – 150 – 200
Theropithecus gelada	DTCSEPVVAMKPSLMFLAKADSRNIAAYCNYTQYQPRVLAVIGPHSSELAVVTGKFFGFFLMPQVSYGASMELLSARETFPSFFRTVPSDRVQLTAAAEL
Papio hamadryas	DTCSEPVVAMKPSLMFLAKADSRNIAAYCNYTQYQPRVLAVIGPHSSELAVVTGKFFGFFLMPQVSYGASMELLSARETFPSFFRTVPSDRVQLTAAAEL
Cercocebus chrysogaster	DTCSEPVVAMKPSLMFLAKADSRDIAAYCNYTQYQPRVLAVIGPHSSELAVVTGKFFGFFLMPQVSYGASMELLSARETFPSFFRTVPSDRVQLTAAAEL
Mandrillus sphinx	DTCSEPVVAMKPSLMFLAKADSRDIAAYCNYTQYQPRVLAVIGPHSSELAVVTGKFFGFFLMPQVSYGASMELLSARETFPSFFRTVPSDRVQLTAAAEL
Macaca assamensis	DTCSEPVVAMKPSLMFLAKADSRDIAAYCNYTQYQPRVLAVIGPHSSELAVVTGKFFGFFLMPQVSYGAGMELLSARETFPSFFRTVPSDRVQLVAAAEL
Macaca arctoides	DTCSEPVVAMKPSLMFLAKADSRDIAAYCNYTQYQPRVLAVIGPHSSELAVVTGKFFGFFLMPQVSYGAGMELLSARETFPSFFRTVPSDRVQLVAAAEL
Macaca fuscata	DTCSEPVVAMKPSLMFLAKADSRDIAAYCNYTQYQPRVLAVIGPHSSELAVVTGKFFGFFLMPQVSYGAGMELLSARETFPSFFRTVPSDRVQLVAAAEL
Cercopithecus mitis	DTCSEPVVAMKPSLMFLAKADSRDIAAYCNYTQYQPRVLAVIGPHSSELAVVTGKFFGFFLMPQVSYGASMELLSARETFPSFFRTVPSDRVQLTAAAEL
Cercopithecus albogularis	DTCSEPVVAMKPSLMFLAKADSRDIAAYCNYTQYQPRVLAVIGPHSSELAVVTGKFFGFFLMPQVSYGASMELLSARETFPSFFRTVPSDRVQLTAAAEL
Erythrocebus patas	DTCSEPVVAMKPSLMFLAKADSRNIAAYCNYTQYQPRVLAVIGPHSSELAVVTGKFFGFFLMPQVSYGASMELLSARETFPSFFRTVPSDRVQLTAAAEL
Chlorocebus sabaceus	DTCSEPVVAMKPSLMFLAKADSRNIAAYCNYTQYQPRVLAVIGPHSSELAVVTGKFFGFFLMPQVSYGASMELLSARETFPSFFRTVPSDRVQLTAAAEL
Nasalis larvatus	DTCSEPVVAMKPSLMFLAKADSRDIAAYCNYTQYQPRVLAVIGPHSSELAVVTGKFFGFFLVPQVSYGASMELLSARETFPSFFRTVPSDRVQLTAAVEL
Pygathrix nemaeus	DTCSEPVVAMKPSLMFLAKADSRDIAAYCNYTQYQPRVLAVIGPYSSELAVVTGRFFGFFLMPQVSYGASMELLSARETFPSFFRTVPSDRVQLTAAVEL
Pygathrix nigripes	DTCSEPVVAMKPSLMFLAKADSRDIAAYCNYTQYQPRVLAVIGPYSSELAVVTGRFFGFFLMPQVSYGASMELLSARETFPSFFRTVLSDRVQLTAAVEL
Rhinopithecus bieti	DTCSEPVVAMKPSLMFLAKADSHDIAAYCNYTQYQPRVLAVIGPHSSELAVVTGKFFGFFLMPQVSYGASMELLSARETFPSFFRTVPSDRVQLTAAVEL
Rhinopithecus brelichi	DTCSEPVVAMKPSLMFLAKADSHDIAAYCNYTQYQPRVLAVIGPHSSELAVVTGKFFGFFLMPQVSYGASMELLSARETFPSFFRTVPSDRVQLTAAVEL
Rhinopithecus roxellana	DTCSEPVVAMKPSLMFLAKADSHDIAAYSNYTQYQPRVLAVIGPHSSELAVVTGKFFGFFLMPQVSYGASMELLSARETFPSFFRTVPSDRVQLTAAVEL
Trachypithecus francoisi	DTCSEPVVAMKPSLMFLAKADSRDIAAYCNYTQYQPRVLAVIGPHSSELAVVTGKFFGFFLMPQVSYGASMELLSARETFPSFFRTVPSDRVQLTAAVEL
Semnopithecus vector	DTCSEPVVAMKPSLMFLAKADSRDIAAYCNYTRYQPRVLAVIGPHSSELAVVTGKFFGFFLMPQVSYGASMELLSARETFPSFFRTVPSDRVQLTAAVEL
Presbytis melalophos	DTCSEPVVAMKPSLMFLAKADSRDIAAYCNYTQYQPRVLAVIGPHSSELAVVTGKFFGFFLMPQVSYGASMELLSARETFPSFFRTVPSDRVQLTAAVEL
Colobus polykomos	DTCSEPVLAMKPSLMFLAKADSHDIAAYCNYTQYQPRVLAVIGPHSSELSVVTGKFFGFFLMPQVSYGASMELLSARETFPSFFRTVPSDRVQLTAAVEL
Hoolock hoolock	DTCSEPVVAMKPSLMFLARADSRDIAAYCNYTQYQPRVLAVIGPHSSELALVTGKFFSFFLMPQVSYGASMELLSARETFPSFFRTVPSDRVQLTAAAEL
Symphalangus syndactylus	DTCSEPVVAMKPSLMFLARADSRDIAAYCNYTRYQPRVLAVIGPHSSELALVTGKFFSFFLMPQVSYGASMELLSARETFPSFFRTVPSDRVQLTAAAEL
Hylobates pileatus	DTCSEPVVAMKPSLMFLARADSRDIAAYCNYTQYQPRVLAVIGPHSSELALVTGKFFSFFLMPQVSYGASMELLSARETFPSFFRTVPSDRVQLTAAAEL
Hylobates lar	DTCSEPVVAMKPSLMFLARADSRDIAAYCNYTQYQPRVLAVIGPHSSELALVTGKFFSFFLMPQVSYGASMELLSARETFPSFFRTVPSDRVQLTAAAEL
Hylobates abbotti	DTCSEPVVAMKPSLMFLARADSRDIAAYCNYTQYQPRVLAVIGPHSSELALVTGKFFSFFLMPQVSYGASMELLSARETFPSFFRTVPSDRVQLTAAAEL
Hylobates agilis	DTCSEPVVAMKPSLMFLARADSRDIAAYCNYTQYQPRVLAVIGPHSSELALVTGKFFSFFLMPQVSYGASMELLSARETFPSFFRTVPSDRVQLTAAAEL
Nomascus annamensis	DTCSEPVVAMKPSLMFLARADSRDIAAYCNYTQYQPRVLAVIGPHSSELALVTGKFFSFFLMPQVSYGASMELLSARETFPSFFRTVPSDRVQLTAAAEL
Nomascus leucogenys	DTCSEPVVAMKPSLMFLARADSRDIAAYCNYTQYQPRVLAVIGPHSSELALVTGKFFSFFLMPQVSYGASMELLSARETFPSFFRTVPSDRVQLTAAAEL
Pan troglodytes	DTCSEPVVAMKPSLVFLAKAGSRDIAAYCNYTQYQPRVLAVIGPHSSELAMVTGKFFSFFLMPQVSYGASMELLSARETFPSFFRTVPSDRVQLTAAAEL
Homo sapiens	DTCSEPVVAMKPSLMFLAKAGSRDIAAYCNYTQYQPRVLAVIGPHSSELAMVTGKFFSFFLMPQVSYGASMELLSARETFPSFFRTVPSDRVQLTAAAEL
Gorilla gorilla gorilla	DTCSEPVVAMKPSLMFLAKAGSRDIAAYCNYTQYQPRVLAVIGPHSSELAMVTGKFFSFFLMPQVSYGASMELLSARETFPSFFRTVPSDRVQLTAAAEL
Pongo abelii	DTCSEPVVAMKPSLMFLAKADSRDIAAYCNYTQYQPRVLAVIGPHSSELALVTGKFFSFFLMPQVSYGASMELLSARETFPSFFRTVPSDRVQLTAAAEL
Pongo pygmaeus	DTCSEPVVAMKPSLMFLAKADSRDIAAYCNYTQYQPRVLAVIGPHSSELALVTGKFFSFFLMPQVSYGASMELLSARETFPSFFRTVPSDRVQLTAAADL
Lophocebus aterrimus	DTCSEPVVAMKPSLMFLAKADSRNIAAYCNYTQYQPRVLAVIGPHSSELAVVTGKFFGFFLMPQVSYGASMELLSARETFPSFFRTVPSDRVQLTAAAEL

	201 250 300
Theropithecus gelada	LQEFGWNWVAALGSDDEYGRQGLSIFSALAAARGICIAHEGLVPLPRANSPLLGKVQEVLHQVNQSSVQVVLLFASPRAAHALFSYSISSKLSRKVWVAS
Papio hamadryas	LQEFGWNWVAALGSDDEYGRQGLSIFSALAAARGICIAHEGLVPLPRANSPLLGKVQEVLHQVNQSSVQVVLLFASARAAHALFSYSISSKLSRKVWVAS
Cercocebus chrysogaster	LQEFGWNWVAALGSDDEYGRQGLSIFSALAAARGICIAHEGLVPLPRANSPLLGKVQEVLHQVNQSSVQVVLLFASARAAHALFSYSISSKLSRKVWVAS
Mandrillus sphinx	LQEFGWNWVAALGSDDEYGRQGLSIFSALAAARGICIAHEGLVPLPRANSPLLGKVQEVLHQVNQSSVQVVLLFASARAAHALFSYSISSKLSRKVWVAS
Macaca assamensis	LQEFGWNWVAALGSDDEYGRQGLSTFSALAASRGICIAHEGLVPLPRANSPLLGKVQEVLHQVNQSSVQVVLLFASARAAHALFSYSISSKLSRKVWVAS
Macaca arctoides	LQEFGWNWVAALGSDDEYGRQGLSTFSALAASRGICIAHEGLVPLPRANSPLLGKVQEVLHQVNQSSVQVVLLFASARAAHALFSYSISSKLSRKVWVAS
Macaca fuscata	LQEFGWNWVAALGSDDEYGRQGLSIFSALAASRGICIAHEGLVPLPRANSPLLGKVQEVLHQVNQSSVQVVLLFASARAAHALFSYSISSKLSRKVWVAS
Cercopithecus mitis	LQEFGWNWVAALGSDDEYGRQGLSIFSALAAARGICIAHEGLVPLPRANSPLLGKVQEVLHQVNQSSVQVVLLFASVRAAHALFSYSISSKLSRKVWVAS
Cercopithecus albogularis	LQEFGWNWVAALGSDDEYGRQGLSIFSALAAARGICIAHEGLVPLPRANSPLLGKVQEVLHQVNQSSVQVVLLFASVRAAHALFSYSISSKLSRKVWVAS
Erythrocebus patas	LQEFGWNWVAALGSDDEYGRQGLSIFSALAAARGICIAHEGLVPLPRANSLLLGKVQEVLHQVNQSSVQVVLLFASARAAHALFSYSISSKLSRKVWVAS
Chlorocebus sabaceus	LQEFGWNWVAALGSDDEYGRQGLSIFSALAAARGICIAHEGLVPLPRANSLLLGKVQEVLHQVNQSSVQVVLLFASARAAHALFSYSISSKLSRKVWVAS
Nasalis larvatus	LQEFGWNWVAALGSDDEYGRQGLGIFSTLAAAHSICIAHEGLVPLPRADGPLLGKVQDVLHQVNQSSVQVVLLFASARAAHALFSYSISSKLSSKVWVAS
Pygathrix nemaeus	LQEFGWNWVAALGSDDEYGRQGLGIFSTLAAAHSICIAHEGLVPLPRADGLLLGKVQDVLHQVNQSSVQVVLLFASAPAAHALFSYSISSKLSSKVWVAS
Pygathrix nigripes	LQEFGWNWVAALGSDDEYGRQGLGIFSTLAAAHSICIAHEGLVPLPRADGLLLGKVQDVLHQVNQSSVQVVLLFASAPAAHALFSYSISSKLSSKVWVAS
Rhinopithecus bieti	LQEFGWNWVAALGSDDEYGRQGLGIFSTLAAAHSICIAHEGLVPLPRADGPLLGKVQDVLHQVNQSSVQVVLLFASARAAHALFSYSISSKLSSKVWVAS
Rhinopithecus brelichi	LQEFGWNWVAALGSDDEYGRQGLGIFSTLAAAHSICIAHEGLVPLPRADGPLLGKVQDVLHQVNQSSVQVVLLFASARAAHALFSYSISSKLSSKVWVAS
Rhinopithecus roxellana	LQEFGWNWVAALGSDDEYGRQGLGIFSTLAAAHSICIAHEGLVPLPRADGPLLGKVQDVLHQVNQSSVQVVLLFASARAAHALFSYSISSKLSSKVWVAS
Trachypithecus francoisi	LQEFGWNWVAALGSDDEYGRQGLGIFSTLAAAHSICIAHEGLVPLPHADGPLLGKVQDVLHQVNQSSVQVVLLFASARAAHALFSYSISSKLSSKVWVAS
Semnopithecus vector	LQEFGWNWVAALGSDDEYGRQGLGIFSTLAAAHSICIAHEGLVPLPHADGPLLGKVQDVLHQVNQSSVQVVLLFASARAAHALFSYSISSKLSSKVWVAS
Presbytis melalophos	LLQFGWNWVAALGSDDEYGRQGLGIFSTLAAAHSICIAHEGLVPLPRADGPLLGKVQDVLHQVNQSSVQVVLLFASARAAHALFSYSISSKLSSKVWVAS
Colobus polykomos	LQEFGWNWVAALGSDDEYGRQGLGIFSTLAAARGICIAHEGLVPLPRADGPLLGKVQDVLHQVNQSSVQVVLLFTSARAAHALFSYSISSKLSPKVWVAS
Hoolock hoolock	LQEFGWNWVAALGSDDEYGRQGLSIFSALAAARGICIAHEGLVPLPHADDLRLGKVQDVLHQVNQSNVQVVLLFASVHAAHALFNYSISSRLSPKVWVAS
Symphalangus syndactylus	LQEFGWNWVAALGSDDEYGRQGLSIFSALAAARGICIAHEGLVPLPRADDLRLGKVQDVLHQVNQSNVQVVLLFASVHAAHALFNYSISSRLSPKVWVAS
Hylobates pileatus	LQQFGWNWVAALGSDDEYGRQGLSIFSALAAARGICIAHEGLVPLPRADDLRLGKVQDVLHQVNQSNVQVVLLFASVHAAHALFNYSISSRLSPKVWVAS
Hylobates lar	LQQFGWNWVAALGSDDEYGRQGLSIFSALAAARGICIAHEGLVPLPRADDLRLGKVQDVLHQVNQSNVQVVLLFASVHAAHALFNYSISSRLSPKVWVAS
Hylobates abbotti	LQQFGWNWVAALGSDDEYGRQGLSIFSALAAARGICIAHEGLVPLPRADDLRLGKVQDVLHQVNQSNVQVVLLFASVHAAHALFNYSISSRLSPKVWVAS
Hylobates agilis	LQQFGWNWVAALGSDDEYGRQGLSIFSALAAARGICIAHEGLVPLPRADDLRLGKVQDVLHQVNQSNVQVVLLFASVHAAHALFNYSISSRLSPKVWVAS
Nomascus annamensis	LQEFGWNWVAALGSDDEYGRQGLSIFSALAAARGICIAHEGLVPLPRADDLRLGKVQDVLHQVNQSNVQVVLLFASVHAAHALFNYSISSKLSPKVWVAS
Nomascus leucogenys	LQEFGWNWVAALGSDDEYGRQGLSIFSALAAARGICIAHEGLVPLPRADDLRLGKVQDVLHQVNQSNVQVVLLFASVHAAHALFNYSISSKLSPKVWVAS
Pan troglodytes	LQEFGWNWVAALGSDDEYGRQGLSIFSALAAARGICIAHEGLVPLPRADDSRLGKVQDVLHQVNQSSVQVVLLFASVHAAHALFNYSISSRLSPKVWVAS
Homo sapiens	LQEFGWNWVAALGSDDEYGRQGLSIFSALAAARGICIAHEGLVPLPRADDSRLGKVQDVLHQVNQSSVQVVLLFASVHAAHALFNYSISSRLSPKVWVAS
Gorilla gorilla gorilla	LQEFGWNWVAALGSDDEYGRQGLSIFSALAAARGICIAHEGLVPLPRADDSRLGKVQDVLHQVNQSSVQVVLLFASVHAAHALFNYSISSRLSPKVWVAS
Pongo abelii	LQQFGWNWVAALGSDDEYGRQGLSIFSALAAARGICIAHEGLVPLPRADDLRLGKVQDVLHQVNQSSVQVVLLFASVHAAYALFNYSISSRLSPKVWVAS
Pongo pygmaeus	LQQFGWNWVAALGSDDEYGRQGLSIFSALAAARGICIAHEGLVPLPRADDLRLGKVQDVLHQVNQSSVQVVLLFASVHAAYALFNYSISSRLSPKVWVAS
Lophocebus aterrimus	LQEFGWNWVAALGSDDEYGRQGLSIFSALAAARGICIAHEGLVPLPRANSPLLGKVQEVLHQVNQSSVQVVLLFASARAAHALFSYSISSKLSRKVWVAS

301 — 350 — 400

Theropithecus gelada	EAWLTSDLVMGLPGMAQVGTVLGFLQRGAQLHKFSQYVKTRLALAADPVFCAALGEREQGLEEDVVGRRCPQCDCITLQNVSAGLNHHQTFSVYAAVYSV
Papio hamadryas	EAWLTSDLVMGLPGMAQVGTVLGFLQRGAQLHKFSQYVKTRLALAADPVFCATLGEREQGLEEDVVGRRCPQCDCITLQNVSAGLNHHQTFSVYAAVYSV
Cercocebus chrysogaster	EAWLTSDLVMGLPGMAQVGTVLGFLQRGAQLHKFSQYVKTRLALAADPAFCAALGEREQGLEEDVVGRRCPQCDCITLQNVSAGLNHHQTFSVYAAVYSV
Mandrillus sphinx	EAWLTSDLVMGLPGMAQVGTVLGFLQRGAQLHKFSQYVKTRLALAADPAFCAALGEREQGLEEDVVGRRCPQCDCITLQNVSAGLNHHQTFSVYAAVYSV
Macaca assamensis	EAWLTSDLVMGLPGMAQVGTVLGFLQRGAQLHKFSQYVKTRLALAADPAFCAALGEREQGLEEDVVGRRCPQCDCITLQNVSAGLNHHQTFSVYAAVYSV
Macaca arctoides	EAWLTSDLVMGLPGMAQVGTVLGFLQRGAQLHKFSQYVKTRLALAADPAFCAALGEREQGLEEDVVGRRCPQCDCITLQNVSAGLNHHQTFSVYAAVYSV
Macaca fuscata	EAWLTSDLVMGLPGMAQVGTVLGFLQRGAQLHKFSQYVKTRLALAADPAFCAALGEREQGLEEDVVGRRCPQCDCITLQNVSAGLNHHQTFSVYAAVYSV
Cercopithecus mitis	EAWLTSDLVMGLPGMAQVGTVLGFLQRGAQLHKFSQYVKTRLALAADPVFCAALGEREQGLEEDVVGRRCPQCDCITLQNVSAGLNHHQTFSVYAAVYSV
Cercopithecus albogularis	EAWLTSDLVMGLPGMAQVGTVLGFLQRGAQLHKFSQYVKTRLALAADPVFCAALGEREQGLEEDVVGRRCPQCDCITLQNVSAGLNHHQTFSVYAAVYSV
Erythrocebus patas	EAWLTSDLVMGLPGMAQVGTVLGFLQRGAQLHKFSQYVKTRLALAADPVFCAALGEREQGLEEDVVGRRCPQCDCITLQNVSAGLNHHQTFSVYAAVYSV
Chlorocebus sabaceus	EAWLTSDLVMGLPGMAQVGTVLGFLQRGAQLHKFSQYVKTRLALAADPVFCTALGEREQGLEEDVVGRRCPQCDCITLQNVSAGLNHHQTFSVYAAVYSV
Nasalis larvatus	EAWLTSDLVMGLPGMAQVGTVLGFLQRGAQLHKFSQYVKTHLALAADPVFCAALGEREQGLEEDVVGRRCPQCDCITLQNVSTGLNHHQTFSVYAAVYSV
Pygathrix nemaeus	EAWLTSDLVMGLPGMAQVGTVLGFLQRGAQLHKFSQYVKTHLALAANPVFCAALGEREQGLEEDVVGRRCPQCDYITLQNVSTGLNHHQTFSVYAAVYSV
Pygathrix nigripes	EAWLTSDLVMGLPGMAQVGTVLGFLQRGAQLHKFSQYVKTHLALAANPVFCAALGEREEGLEEDVVGRRCPQCDYITLQNVSTGLNHHQTFSVYAAVYSV
Rhinopithecus bieti	EAWLTSDLVMGLPGMAQVGTVLGFLQRGAQLHKFSQYVKTHMALAADPVFCAALGKREQGLEEDVVGRRCPQCDCITLQNVSTGLNHHQTFSVYAAVYSV
Rhinopithecus brelichi	EAWLTSDLVMGLPGMAQVGTVLGFLQRGAQLHKFSQYVKTHLALAADPVFCAALGKREQGLEEDVVGRRCPQCDCITLQNVSTGLNHHQTFSVYAAVYSV
Rhinopithecus roxellana	EAWLTSDLVMGLPGMAQVGTVLGFLQRGAQLHKFSQYVKTHLALAADPVFCAALGKREQGLEEDVVGRRCPQCDCITLQNVSTGLNHHQTFSVYAAVYSV
Trachypithecus francoisi	EAWLTSDLVMGLPGMAQVGTVLGFLQRGAQLHKFSQYVKSHLALAADPVFCAALGEREQGLEEDVVGRRCPQCDCITLQNVSTGLNHHQTFSVYAAVYSV
Semnopithecus vector	EAWLTSDLVMGLPGMAQVGTVLGFLQRGAQLHKFSQYVKTHLALAADPVFCAALGEREQGLEEDVVGRRCPQCDCITLQNVSTGLNHHQTFSVYAAVYSV
Presbytis melalophos	EAWLTSDLVMGLPGMAQVGTVLGFLQRGAQLHKFSQYVKTHLALAADPVFCAALGEREQGLEEDVVGRRCPQCDCITLQNVSTGLNHHQTFSVYAAVYSV
Colobus polykomos	EAWLTSDLVMGLPGMAQVGTVLGFLQRGAQLHKFSQYVKTHLALAADPVFCAALGEREQGLEEDVVGRRCPQCDCITLQNVSAGLNHHQTFSVYAAVYSV
Hoolock hoolock	EAWLTSDLVMGLPGMAQVGTVLGFLQRGAQLHEFSQYVKTHLALAADPAFCATLGKREQVLEEDVVGQRCPQCDCITLQNVSAGLNHHQTFSVYAAVYSV
Symphalangus syndactylus	EAWLTSDLVMGLPGMAQVGTVLGFLQRGAQLHEFSQYVKTHLALAADPAFCATLGKREQVLEEDVVGQRCPQCDCITLQNVSTGLNHHQTFSVYAAVYSV
Hylobates pileatus	EAWLTSDLVMGLPGMAQVGTVLGFLQRGAQLHEFSQYVKTHLALAADPAFCATLGKREQVLEEDVVGQRCPQCDCITLQNVSAGLNHHQTFSVYAAVYSV
Hylobates lar	EAWLTSDLVMGLPGMAQVGTVLGFLQRGAQLHEFSQYVKTHLALAADPAFCATLGKREQVLEEDVVGQRCPQCDCITLQNVSAGLNHHQTFSVYAAVYSV
Hylobates abbotti	EAWLTSDLVMGLPGMAQVGTVLGFLQRGAQLHEFSQYVKTHLALAADPAFCATLGKREQVLEEDVVGQRCPQCDCITLQNVSAGLNHHQTFSVYAAVYSV
Hylobates agilis	EAWLTSDLVMGLPGMAQVGTVLGFLQRGAQLHEFSQYVKTHLALAADPAFCATLGKREQVLEEDVVGQRCPQCDCITLQNVSAGLNHHQTFSVYAAVYSV
Nomascus annamensis	EAWLTSDLVMGLPGMAQVGTVLGFLQRGAQLHEFSQYVKTHLALAADPAFCATLGKREQVLEEDVVGQRCPQCDCITLQNVSAGLNHHQTFSVYAAVYSV
Nomascus leucogenys	EAWLTSDLVMGLPGMAQVGTVLGFLQRGAQLHEFSQYVKTHLALAADPAFCATLGKREQVLEEDVVGQRCPQCDCITLQNVSAGLNHHQTFSVYAAVYSV
Pan troglodytes	EAWLTSDLVMGLPGMAQMGTVLGFLQRGAQLHEFPQYVKTHLALAADPAFCSALGEREQGLEEDVVGQRCPQCDCITLQNVSAGLNHHQTFSVYAAVYSV
Homo sapiens	EAWLTSDLVMGLPGMAQMGTVLGFLQRGAQLHEFPQYVKTHLALATDPAFCSALGEREQGLEEDVVGQRCPQCDCITLQNVSAGLNHHQTFSVYAAVYSV
Gorilla gorilla gorilla	EAWLTSDLVMGLPGMAQMGTVLGFLQRGAQLHEFPQYVKTHLALAADPAFCSTLGEREQGLEEDVVGQRCPQCDCITLQNVSAGLNHHQTFSVYTAVYSV
Pongo abelii	EAWLTSDLIMGLPGMAQVGTVLGFLQKGAQLHEFSQYVKTHLALAADPAFCAALGEREQGLEEDVVGQRCPQCDCITLQNVSAGLNHHQMFSVYAAVYSV
Pongo pygmaeus	EAWLTSDLIMGLPGMAQVGTVLGFLQKGAQLHEFSQYVKTHLALAADPAFCAALGEREQGLEEDVVGQRCPQCDCITLQNVSAGLNHHQMFSVYAAVYSV
Lophocebus aterrimus	EAWLTSDLVMGLPGMAQVGTVLGFLQRGAQLHKFSQYVKTRLALAADPVFCAALGEREQGLEEDVVGRRCPQCDCITLQNVSAGLNHHQTFSVYAAVYSV

401 450 500

Theropithecus gelada	AQALHNTLQCNASGCPVQDPVKPWQLLDNMYNLTFHAGGLTLRFNSNGNVDMEYDLKLWVWQGPVPELHDVGRFNGSLWIDSLKIRWHTSNNQKPVSQCS
Papio hamadryas	AQALHNTLQCNASGCPVQDPVKPWQLLDNMYNLTFHAGGLTLRFNSNGNVDMEYDLKLWVWQGPVPELHDVGRFNGSLWIDSLKIRWHTSNNQKPVSQCS
Cercocebus chrysogaster	AQALHNTLHCNASGCPVQEPVKPWQLLDNMYNLTFHAGGLTLRFNSNGNVDMEYDLKLWVWQGPVPELHNVGRFNGSLWIDSLKIRWHTSNNQKPVSQCS
Mandrillus sphinx	AQALHNTLHCNASGCPVQEPVKPWQLLDNMYNLTFHAGGLTLRFNSNGNVDMEYDLKLWVWQGPVPELHDVGRFNGSLWIDSLKIRWHTSNNQKPVSQCS
Macaca assamensis	AQALHNALQCSASGCPVQDPVKPWQLLENMYNLTFHAGGLTLRFNSNGNVDMEYDLKLWVWQGPVPELHDVGRFNGNLWIDSPKIRWHTSNNQKPVSQCS
Macaca arctoides	AQALHNALQCSASGCPVQDPVKPWQLLENMYNLTFHAGGLTLRFNSNGNVDMEYDLKLWVWQGPVPELHDVGRFNGSLWIDSPKIRWHTSNNQKPVSQCS
Macaca fuscata	AQALHNALQCSASGCPVQDPVKPWQLLENMYNLTFHAGGLTLRFNSNGNVDMEYDLKLWVWQGPVPELHDVGRFNGSLWIDSPKIRWHTSNNQKPVSQCS
Cercopithecus mitis	AQALHNTLQCNASGCPMRDPVKPWQLLENMYNLTFHAGGLMLRFNSNGNVDMEYDLKLWVWQGPVPELHDVGRFNGSLWVDSLKIRWHTSNNQMPVSQCS
Cercopithecus albogularis	AQALHNTLQCNASGCPMRDPVKPWQLLENMYNLTFHAGGLMLRFNSNGNVDMEYDLKLWVWQGPVPELHDVGRFNGSLWVDSLKIRWHTSNNQMPVSQCS
Erythrocebus patas	AQALHSTLQCNASGCPMRDPVKPWQLLENMYNLTFHAGGLTLRFNSNGNVDMEYDLKLWVWQGPVPELHDVGRFNGSLWIDSLKIHWHTSNNQKPVSQCS
Chlorocebus sabaceus	AQALHNTLQCNASGCPMRDPVKPWQLLENMYNLTFHAGGLTLRFNSNGNVDMEYDLKLWVWQGPVPELHDVGRFNGSLWIDSLKIHWHTSNNQKPVSQCS
Nasalis larvatus	AQALHNTLQCNASGCPMQDPVKPWQLLQNMYNLTFHAGGLTLRFNSNGNVDMEYDLKLWVWQGPVPELHDVGRFNGSLWTDSLKIRWHTSNNQKPVSQCS
Pygathrix nemaeus	AQALHNTLQCNASGCPMQDPVKPWQLLENMYNLTFHAGGLTLRFNSNGNVDMEYDLKLWVWQGPVPELHDVGRFNGSLWTDSLKIRWHTSNNQKPVSQCS
Pygathrix nigripes	AQALHNTLQCNASGCPMQDPVKPWQLLENMYNLTFHAGGLTLRFNSNGNVDMEYDLKLWVWQGPVPELHDVGRFNGSLWTDSLKIRWHTSNNQKPVSQCS
Rhinopithecus bieti	AQALHNTLQCNASGCPMQDPVKPWQLLENMYNLTFHAGGLTLRFNSNGNVDMEYDLKLWVWQGSVPELHDVGRFNGSLWTDSLKIRWHTSNNQKPVSQCS
Rhinopithecus brelichi	AQALHNTLQCNASGCPMQDPVKPWQLLENMYNLTFHAGGLTLRFNSNGNVDMEYDLKLWVWQGSVPELHDVGRFNGSLWTDSLKIRWHTSNNQKPVSQCS
Rhinopithecus roxellana	AQALHNTLQCNASGCPMQDPVKPWQLLENMYNLTFHAGGLTLRFNSNGNVDMEYDLKLWVWQGSVPELHDVGRFNGSLWTDSLKIRWHTSNNQKPVSQCS
Trachypithecus francoisi	AQALHNTLQCNASGCPMQDPVKPWQLLENMYNLTFHAGGLTLHFNSNGNVDMEYDLKLWVWQGPVPELHDVGRFNGSLWTDSLKIRWHTSNNQKPVSQCS
Semnopithecus vector	AQALHNTLQCNASGCPMQDPVKPWQLLENMYNLTFHAGGLTLHFNSNGNVDMEYDLKLWVWQGPVPELHDVGRFNGSLWTDSLKIRWHTSNNQKPVSQCS
Presbytis melalophos	AQALHNTLQCNASGCPMQDPVKPWQLLENMYNLTFHAGGLTLRFNSNGNVDMEYDLKLWVWQGPVPELHNVGRFNGSLWTDSLKIRWHTSNHQKPVSQCS
Colobus polykomos	AQALHNTLQCNASGCPMQDPVKPWQLLENMYNLTFHAGGLTLRFNSNGNVDMEYDLKLWVWQGPVPELHDVGRFNGSLWTDSLKIRWHTSNNQKPVSQCS
Hoolock hoolock	AQALHNTLQCNASGCPTQDPVKPWQLLENMYNLTFHAGGLTLRFNSSGNVDMEYDLKLWVWQGSVPKLHNVGRFNGSLWTEHLKIRWHTLDNQKPVSQCS
Symphalangus syndactylus	AQALHNTLQCNASGCPTQDPVKPWQLLENMYNLTFHAGGLTLRFNSSGNVDMEYDLKLWVWQGSVPKLHNVGRFNGSLWTERLKIRWHTLDNQKPVSQCS
Hylobates pileatus	AQALHNTLQCNASGCPTQDPVKPWQLLENMYNLTFHAGGLTLRFNSSGNVDMEYDLKLWVWQGSVPKLHNVGRFNGSLWTEHLKIRWHTLDNQKPVSQCS
Hylobates lar	AQALHNTLQCNASGCPTQDPVKPWQLLENMYNLTFHAGGLTLRFNSSGNVDMEYDLKLWVWQGSVPKLHNVGRFNGSLWTEHLKIRWHTLDNQKPVSQCS
Hylobates abbotti	AQALHNTLQCNASGCPTQDPVKPWQLLENMYNLTFHAGGLTLRFNSSGNVDMEYDLKLWVWQGSVPKLHNVGRFNGSLWTEHLKIRWHTLDNQKPVSQCS
Hylobates agilis	AQALHNTLQCNASGCPTQDPVKPWQLLENMYNLTFHAGGLTLRFNSSGNVDMEYDLKLWVWQGSVPKLHNVGRFNGSLWTEHLKIRWHTLDNQKPVSQCS
Nomascus annamensis	AQALHNTLQCNASGCPTQDPVKPWQLLENMYNLTFHAGGLTLRFNSSGNVDMEYDLKLWVWQGSVPKLHNVGRFNGSLWTEHLKIRWHTLDNQKPVSQCS
Nomascus leucogenys	AQALHNTLQCNASGCPTQDPVKPWQLLENMYNLTFHAGGLTLRFNSSGNVDMEYDLKLWVWQGSVPKLHNVGRFNGSLWTEHLKIRWHTLDNQKPVSQCS
Pan troglodytes	AQALHNTLQCNASGCPAQDPVKPWQLLENMYNLTFHAGGLMLRFDSSGNVDMEYDLKLWVWQGSVPRLHDVGRFNGSLRTERLKIRWHTSDNQKPVSRCS
Homo sapiens	AQALHNTLQCNASGCPAQDPVKPWQLLENMYNLTFHVGGLPLRFDSSGNVDMEYDLKLWVWQGSVPRLHDVGRFNGSLRTERLKIRWHTSDNQKPVSRCS
Gorilla gorilla gorilla	AQALHNTLQCNASGCPAQDPVKPWQLLENMYNLTFHAGGLTLRYDSSGNVDMEYDLKLWVWQGSVPRLHDVGRFNGSLRTERLKIRWHTSDNQKPVSRCS
Pongo abelii	AQALHNTLQCNASGCPAQDPVKPWQLLENMYNLTFHVGGLTLRFNSSGNVDMEYDLKLWVWQGSVPKLHNVGGFNGSLWTERLKIRWHTPDNQKPVSQCS
Pongo pygmaeus	AQALHNTLQCNASGCPAQDPVKPWQLLENMYNLTFHVGGLTLRFNSSGNVDMEYDLKLWVWQGSVPKLHNVGGFNGSLWTERLKIRWHTPDNQKPVSQCS
Lophocebus aterrimus	AQALHNTLQCNASGCPVQDPVKPWQLLDNMYNLTFHAGGLTLRFNSNGNVDMEYDLKLWVWQGPVPELHDVGRFNGSLWIDSLKIRWHTSNNQKPVSQCS

Species	501 … 550 … 600
Theropithecus gelada	RQCQEGQVRRVKGFHSCCYDCVDCKAGSYRKSPDDLACTFCSQDEWSPERSTRCFRRRLRFLAWGEPAVLLLLLLFGLALGLVLAALGLFIRHRDSPLVQ
Papio hamadryas	RQCQEGQVRRVKGFHSCCYDCVDCKAGSYRKSPDDLACTFCSQDEWSPERSTRCFRRRLRFLAWGEPAVLLLLLLFGLALGLVLAALGLFIRHRDSPLVQ
Cercocebus chrysogaster	RQCQEGQVRRVKGFHSCCYDCVDCKAGSYRKSPDDLACTFCSQDEWSPERSTRCFRRRLRFLAWGEPAVLLLLLLFGLALGLVLAALGLFIRHRDSPLVQ
Mandrillus sphinx	RQCQEGQVRRVKGFHSCCYDCVDCKAGSYRKSPDDLACTFCSQDEWSPERSTRCFRRRLRFLAWGEPAVLLLLLLFGLALGLVLAALGLFIRHRDSPLVQ
Macaca assamensis	RQCQEGQVRRVKGFHSCCYDCVDCKAGSYRKSPDDLACTFCGQEEWSPERSTRCFRRRLRFLAWGEPAVLLLLLLFGLALGLVLAALGLFIRHRDSPLVQ
Macaca arctoides	RQCQEGQVRRVKGFHSCCYDCVDCKAGSYRKSPDDLACTFCGQEEWSPERSTRCFRRRLRFLAWGEPAVLLLLLLFGLALGLVLAALGLFIRHRDSPLVQ
Macaca fuscata	RQCQEGQVRRVKGFHSCCYDCVDCKAGSYRKSPDDFACTFCGREEWSPERSTRCFRRRLRFLAWGEPAVLLLLLLFGLALGLVLAALGLFIRHRDSPLVQ
Cercopithecus mitis	RQCQEGQVRRVKGFHSCCYDCVDCKAGSYRKSPDDLACTFCSQEEWSPERSTRCFRRRLRFLAWGEPAVLLLLLLLGLALGLVLAALGLFIRHRDSPLVQ
Cercopithecus albogularis	RQCQEGQVRRVKGFHSCCYDCVDCKAGSYRKSPDDLACTFCSQEEWSPERSTRCFRRRLRFLAWGEPAVLLLLLLLGLALGLVLAALGLFIRHRDSPLVQ
Erythrocebus patas	RQCQEGQVRRVKGFHSCCYDCVDCKAGSYRKSPDDLACTFCSQDEWSPERSTQCFRRRLRFLAWGEPAVLLLLLLLSLALGLVLAALGLFIRHRDSPLVQ
Chlorocebus sabaceus	RQCQEGQVRRVKGFHSCCYDCVDCKAGSYRKSPDDLACTFCSQDEWSPERSTRCFRRRLRFLAWGEPAVLLLLLLLGLALGLVLAALGLFIRHRDSPLVQ
Nasalis larvatus	RQCQEGQVRRVKGFHSCCYDCVDCKAGSYQKSPDDLACSFCNQDEWSPERSTRCFRRRLRFLAWGEPAVLLLLLLLSLALGLVLAALGLFIRHRDSPLVQ
Pygathrix nemaeus	RQCQEGQVRRVKGFHSCCYDCVDCKAGSYQKSPDDLACSFCNQDEWSPERSTRCFRRRLRFLAWGEPAVLLLLLLLSLALGLVLAALGLFIRHRDSPLVQ
Pygathrix nigripes	RQCQEGQVRRVKGFHSCCYDCVDCKAGSYQKSPDDLACSFCNQDEWSPERSTRCFRRRLRFLAWGEPAVLLLLLLLSLALGLVLAALGLFIRHRDSPLVQ
Rhinopithecus bieti	WQCQEGQVRRVKGFHSCCYDCVDCKAGSYQKSPDDLACSFCNQDEWSPERSTRCFRRRLRFLAWGEPAVLLLLLLLSLALGLVLAALGLFIRHRDSPLVQ
Rhinopithecus brelichi	WQCQEGQVRRVKGFHSCCYDCVDCKAGSYQKSPDDLACSFCNQDEWSPERSTRCFRRRLRFLAWGEPAVLLLLLLLSLALGLVLAALGLFIRHRDSPLVQ
Rhinopithecus roxellana	WQCQEGQVRRVKGFHSCCYDCVDCKAGSYQKSPDDLACSFCNQDEWSPERSTRCFRRRLRFLAWGEPAVLLLLLLLSLALGLVLAALGLFIRHRDSPLVQ
Trachypithecus francoisi	RQCQEGQVRRVKGFHSCCYDCVDCKAGSYQKSPDDLACSFCNQDEWSPERSTSCFRRRLRFLAWGEPAVLLLLLLLSLALGLVLAALGLFIRHRDSPLVQ
Semnopithecus vector	RQCQEGQVRRVKGFHSCCYDCVDCKAGSYQKSPDDLACSFCNQDEWSPERSTSCFRRRLRFLAWGEPAVLLLLLLLSLALGLVLAALGLFIRHRDSPLVQ
Presbytis melalophos	RQCQEGQVRRVKGFHSCCYDCVDCKAGSYRKSPDDLACTFCNQDEWSPERSTRCFRRRLRFLAWGEPVVLLLLLLLSLALGLVLAALGLFIRHRDSPLVQ
Colobus polykomos	RQCQEGQVRRVKGFHSCCYDCVDCKAGSYRKSPDDLACSFCNQDEWSPERSTRCFRRRLRFLAWGEPAVLLLLLLLSLALGLVLAALGLFIRHRDSPLVQ
Hoolock hoolock	QQCQEGQVRRVKGFHSCCYDCVDCKAGSYRHSPDDLACTFCRQDEWSPERSTRCFRRRYRFLAWGEPAVLLLLLLLSLALGLVLAALGLFIRHRDSPLVQ
Symphalangus syndactylus	QQCQEGQVRRVKGFHSCCYDCVDCKAGSYRHSPDDLACTFCRQDEWSPERSTRCFRRRYRFLAWGEPAVLLLLLLLSLALGLVLAALGLFIRHRDSPLVQ
Hylobates pileatus	QQCQEGQVRRVKGFHSCCYDCVDCKAGSYRHSPDDLACTFCRQDEWSPERSTRCFRRRYRFLAWGEPAVLLLLLLLSLALGLVLAALGLFIRHRDSPLVQ
Hylobates lar	QQCQEGQVRRVKGFHSCCYDCVDCKAGSYRHSPDDLACTFCRQDEWSPERSTRCFRRRYRFLAWGEPAVLLLLLLLSLALGLVLAALGLFIRHRDSPLVQ
Hylobates abbotti	QQCQEGQVRRVKGFHSCCYDCVDCKAGSYRHSPDDLACTFCRQDEWSPERSTRCFRRRYRFLAWGEPAVLLLLLLLSLALGLVLAALGLFIRHRDSPLVQ
Hylobates agilis	QQCQEGQVRRVKGFHSCCYDCVDCKAGSYRHSPDDLACTFCRQDEWSPERSTRCFRRRYRFLAWGEPAVLLLLLLLSLALGLVLAALGLFIRHRDSPLVQ
Nomascus annamensis	QQCQEGQVRRVKGFHSCCYDCVDCKAGSYRHSPDDLACTFCRQDEWSPERSTRCFRRRYRFLAWGEPAVLLLLLLLSLALGLVLAALGLFIRHRDSPLVQ
Nomascus leucogenys	QQCQEGQVRRVKGFHSCCYDCVDCKAGSYRHSPDDLACTFCRQDEWSPERSTRCFRRRYRFLAWGEPAVLLLLLLLSLALGLVLAALGLFIRHRDSPLVQ
Pan troglodytes	RQCQEGQVRRVKGFHSCCYDCVDCEAGSYRQNPDDIACTFCGQDEWSPERSTRCFRRRSRFLAWGEPAVLLLLLLLSLALGLVLAALGLFIHHRDSPLVQ
Homo sapiens	RQCQEGQVRRVKGFHSCCYDCVDCEAGSYRQNPDDIACTFCGQDEWSPERSTRCFRRRSRFLAWGEPAVLLLLLLLSLALGLVLAALGLFVHHRDSPLVQ
Gorilla gorilla gorilla	RQCQEGQVRRVKGFHSCCYDCVDCEAGSYRQNPDDVTCTSCGQDEWSPERSTRCFHRRSRFLAWGEPAVLLLLLLLSLALGLVLAALGLFVHHRDSPLVQ
Pongo abelii	RQCQEGQVRRVKGFHSCCYDCVDCKAGSYRHSPDDLACTFCRQDEWSPERSTRCFRRRSRFLAWGEPAVLLLLLLLSLALGLVLAALGLFIRHRDSPLVR
Pongo pygmaeus	RQCQEGQVRRVKGFHSCCYDCVDCKAGSYRHSPDDLACTFCRQDEWSPERSTRCFRRRSRFLAWGEPAVLLLLLLLSLALGLVLAALGLFIRHRDSPLVR
Lophocebus aterrimus	RQCQEGQVRRVKGFHSCCYDCVDCKAGSYRKSPDDLACTFCSQDEWSPERSTRCFRRRLRFLAWGEPAVLLLLLLFGLALGLVLAALGLFIRHRDSPLVQ

	601 — 650 — 700
Theropithecus gelada	ASGGLLACFSLVCLGLVCISVLLFPGQPSPARCLAQQPSSHLPLTGCLSTFILQAAEIFVESELPLSWADRLSGCLRGPWAWLVVLVAMLVEAALCAWYL
Papio hamadryas	ASGGLLACFSLVCLGLVCISVLLFPGQPSPARCLAQQPSSHLPLTGCLSTFILQAAEIFVESELPLSWADRLSGCLRGPWAWLVVLLAMLVEAALCAWYL
Cercocebus chrysogaster	ASGGPLACFGLVCLGLVCISVLLFPGQPSPARCLAQQPSSHLPLTGCLSTFILQAAEIFMESELPLSWADRLSGCLRGPWAWLVVLLAMLVEAALCAWYL
Mandrillus sphinx	ASGGPLACFGLVCLGLVCISVLLFPGQPSPARCLAQQPSSHLPLTGCLSTFILQAAEIFVESELPLSWADRLSGCLRGPWAWLVVLLAMLVEAALCAWYL
Macaca assamensis	ASGGPLACFGLVCLGLVCISVLLFPGQPSPARCLAQQPSSHLPLTGCLSTFILQAAEIFAESELPLSWADRLSGCLRGPWAWLVVLLAMLVEAALCAWYL
Macaca arctoides	ASGGPLACFGLVCLGLVCISVLLFPGQPSPARCLAQQPSSHLPLTGCLSTFILQAAEIFAESELPLSWADRLSGCLRGPWAWLVVLLAMLVEAALCAWYL
Macaca fuscata	ASGGPLACFGLVCLGLVCISVLLFPGQPSPARCLAQQPSSHLPLTGCLSTFILQAAEIFVESELPLSWADRLSGCLRGPWAWLVVLLAMLVEAALCAWYL
Cercopithecus mitis	ASGGPLACFGLVCLGLVCLSVLLFPGQPSPARCLAQQPSSHLPLTGCLSTLILQAAEIFVESELPLSWADRLSGCLRGPGAWLVVLLAMLVEAALCAWYL
Cercopithecus albogularis	ASGGPLACFGLVCLGLVCLSVLLFPGQPSPARCLAQQPSSHLPLTGCLSTLILQAAEIFVESELPLSWADRLSGCLRGPGAWLVVLLAMLVEAALCAWYL
Erythrocebus patas	ASGGPLACFGLVCLGLVCISVLLFPGQPSPARCLAQQPSSHLPLTGCLSTLILQAAEIFVESELPLSWADRLSGCLRGPWAWLVVLLAMLVEAALCTWYL
Chlorocebus sabaceus	ASGGPLACFGLVCLGLVCISVLLFPGQPSPARCLAQQPSSHLPLTGCLSTLILQAAEIFVESELPLSWADRLSGCLRGPWAWLVVLLAVLVEAALCAWYL
Nasalis larvatus	ASGGPLACFGLVCLGLVCLSVLLFPGQPSPALCLAQQPSSHLPLTGCLSTLILQAAEIFVESELPLSWADRLSGCLRGPWAWLVVLLAMLVEAALCAWYL
Pygathrix nemaeus	ASGGPLACFGLVCLGLVCLSVLLFPGQPSPALCLAQQPSSHLPLTGCLSTFILQAAEIFVESELPLSWADRLSGCLRGPWAWLVVLLAMLVEAALCAWYL
Pygathrix nigripes	ASGGPLACFGLVCLGLVCLSVLLFPGQPSPALCLAQQPSSHLPLTGCLSTFILQAAEIFVESELPLSWADRLSGCLRGPWAWLVVLLAMLVEAALCAWYL
Rhinopithecus bieti	ASGGPLACFGLVCLGLVCLSVLLFPGQPSPALCLAQQPSSHLPLTGCLSTLILQAAEIFVESELPLSWADRLSGCLRGPWAWLVVLLAMLVEAALCAWYL
Rhinopithecus brelichi	ASGGPLACFGLVCLGLVCLSVLLFPGQPSPALCLAQQPSSHLPLTGCLSTLILQAAEIFVESELPLSWADRLSGCLRGPWAWLVVLLAMLVEAALCAWYL
Rhinopithecus roxellana	ASGGPLACFGLVCLGLVCLSVLLFPGQPSPALCLAQQPSSHLPLTGCLSTLILQAAEIFVESELPLSWADRLSGCLRGPWAWLVVLLAMLVEAALCAWYL
Trachypithecus francoisi	ASGGPLACFGLVCLGLVCLSVLLFPGQPSPALCLAQQPSSHLPLTGCLSTLILQAAEIFVESELPLSWADRLSGCLRGPWAWLVVLLAMLVEAALCAWYL
Semnopithecus vector	ASGGPLACFGLVCLGLVCLSVLLFPGQPSPALCLAQQPSSHLPLTGCLSTLILQAAEIFVESELPLSWADRLSGCLRGPWAWLVVLLAMLVEAALCAWYL
Presbytis melalophos	ASGGPLACFGLVCLGLVCLSVLLFPGQPSPALCLAQQPSSHLPLTGCLSTLILQAAEIFVESELPLSWADRLSGCLRGPWAWLVVLLAMLVEAALCAWYL
Colobus polykomos	ASGGPLACFGLVCLGLVCLSVLLFPGQPSSALCLAQQPSSHLPLTGCLSTLILQAAEIFVESELPLSWADRLSGCLRGPWAWLVVLLAMLVEAALCAWYL
Hoolock hoolock	ASGGPLACFGLVCLGLVCLSVLLFPGQPSPARCLAQQPLSHLPLTGCLSTLFLQAAEIFVESELPLSWADRLSGCLRGPWAWLVVLLAMLVEAALCTWYL
Symphalangus syndactylus	ASGGPLACFGLVCLGLVCLSVLLFPGQPSPARCLAQQPLSHLPLTGCLSTLFLQAAEIFVESELPLSWADRLSGCLRGPGAWLVVLLAMLVEAALCTWYL
Hylobates pileatus	ASGGPLACFGLVCLGLVCLSVLLFPGQPSPARCLAQQPLSHLPLTGCLSTLFLQAAEIFVESELPLSWADRLSGCLRGPWAWLVVLLAMLVEAALCTWYL
Hylobates lar	ASGGPLACFGLVCLGLVCLSVLLFPGQPSPARCLAQQPLSHLPLTGCLSTLFLQAAEIFVESELPLSWADRLSGCLRGPWAWLVVLLAMLVEAALCTWYL
Hylobates abbotti	ASGGPLACFGLVCLGLVCLSVLLFPGQPSPARCLAQQPLSHLPLTGCLSTLFLQAAEIFVESELPLSWADRLSGCLRGPWAWLVVLLAMLVEAALCTWYL
Hylobates agilis	ASGGPLACFGLVCLGLVCLSVLLFPGQPSPARCLAQQPLSHLPLTGCLSTLFLQAAEIFVESELPLSWADRLSGCLRGPWAWLVVLLAMLVEAALCTWYL
Nomascus annamensis	ASGGPLACFGLVCLGLVCLSVLLFPGQPSPARCLAQQPLSHLPLTGCLSTLFLQAAEIFVESELPLSWADRLSGCLRGPWAWLVVLLAMLVEAALCTWYL
Nomascus leucogenys	ASGGPLACFGLVCLGLVCLSVLLFPGQPSPARCLAQQPLSHLPLTGCLSTLFLQAAEIFVESELPLSWADRLSGCLRGPWAWLVVLLAMLVEAALCTWYL
Pan troglodytes	ASGGPLACFGLVCLGLVCLSVLLFPGQPSPARCLAQQPLSHLPLTGCLSTLFLQAAEIFVESELPLSWADRLSGCLRGPWAWLVVLLAMLVEVALCTWYL
Homo sapiens	ASGGPLACFGLVCLGLVCLSVLLFPGQPSPARCLAQQPLSHLPLTGCLSTLFLQAAEIFVESELPLSWADRLSGCLRGPWAWLVVLLAMLVEVALCTWYL
Gorilla gorilla gorilla	ASGGPLACFGLVCLGLVCLSVLLFPGQPSPAQCLAQQPLSHLPLTGCLSTLFLQAAEIFVESELPLSWADRLSGCLRGPWAWLVVLLAMLVEVALCTWYL
Pongo abelii	ASGGPLACFGLVCLGLVCLSVLLFPGRPGTARCLAQQPLSHLPLTGCLSTLFLQAAEIFVESELPLSWADRLSGCLRGPWAWLVVLLAMLVEVALCTWYL
Pongo pygmaeus	ASGGPLACFGLVCLGLVCLSVLLFPGRPGTARCLAQQPLSHLPLTGCLSTLFLQAAEIFVESELPLSWADRLSGCLRGPWAWLVVLLAMLVEVALCTWYL
Lophocebus aterrimus	ASGGLLACFSLVCLGLVCISVLLFPGQPSPARCLAQQPSSHLPLTGCLSTFILQAAEIFVESELPLSWADRLSGCLRGPWAWLVVLLAMLVEAALCAWYL

701 750 800

Theropithecus gelada	VAFPPEMVTDWRMLPTEALVHCRARSWVSFGLVHATNATLAFLCFLGTFLVQSRPGRYNRARGLTFAMLAYFITWVSFVPLLANVQVVLRPAVQMGALLL
Papio hamadryas	VAFPPEVVMDWRMLPTEALVHCRARSWVSFGLVHATNATLAFLCFLGTFLVQSRPGRYNRARGLTFAMLAYFITWVSFVPLLANVQVVLRPAVQMGALLL
Cercocebus chrysogaster	VAFPPEVVTDWRMLPTEALVHCRARSWVSFGLVHATNATLAFLCFLGTFLVQSRPGRYNRARGLTFAMLAYFITWVSFVPLLANVQVVFRPAVQMGALLL
Mandrillus sphinx	VAFPPEVVTDWRMLPTEALVHCRARSWVSFGLVHATNATLAFLCFLGTFLVQSRPGRYNRARGLTFAMLAYFITWVSFVPLLANVQVVFRPAVQMGALLL
Macaca assamensis	VAFPPEVVTDWRMLPTEALVHCRTRSWVSFGLVHATNATLAFLCFLGTFLVQSRPGRYNRARGLTFAMLAYFITWVSFVPLLANVQVVLRPAVQMGALLL
Macaca arctoides	VAFPPEVVTDWRMLPTEALVHCRTRSWVSFGLVHATNATLAFLCFLGTFLVQSRPGRYNRARGLTFAMLAYFITWVSFVPLLANVQVVLRPAVQMGALLL
Macaca fuscata	VAFPPEVVTDWRMLPTEALVHCRTRSWVSFGLVHATNTTLAFLCFLGTFLVQSRPGRYNRARGLTFAMLAYFITWVSFVPLLANVQVVLRPAVQMGALLL
Cercopithecus mitis	VAFPPEVVTDWRMLPTEALVHCRARSWVSFGLVHATNATLAFLCFLGTFLVQSRPGRYNRARGLTFAMLAYFITWVSFVPLLANVQVVLRPAVQMGALLL
Cercopithecus albogularis	VAFPPEVVTDWRMLPTEALVHCRARSWVSFGLVHATNATLAFLCFLGTFLVQSRPGRYNRARGLTFAMLAYFITWVSFVPLLANVQVVLRPAVQMGALLL
Erythrocebus patas	MAFPPEVVTDWRMLPTEALVHCRARSWVSFGLVHATNATLAFLCFLGTFLVQSRPGRYNRARGLTFAMLAYFITWVSFVPLLANVQVVLRPAVQMGALLL
Chlorocebus sabaceus	MVFPPEVVTDWRMLPTEALVHCRARSWVSFGLVHATNATLAFLCFLGTFLVQSRPGRYNRARGLTFAMLAYFITWVSFVPLLANVQVVLRPAVQMGALLL
Nasalis larvatus	VAFPPEVVTDWRMLPTEVLVHCRTRSWVSFGLVHATNATLAFLCFLGTFLVQSRPGRYNRARGLTFAMLAYFITWVSFVPLLANVQVVLRPAVQMGALLL
Pygathrix nemaeus	VAFPPEVVTDWRLLPTEALVHCRTRSWVSFGLVHATNATLAFLCFLGTFLVQSRPGHYNRARGLTFAMLAYFITWVSFVPLLANVQVVLRPAVQMGALLL
Pygathrix nigripes	VAFPPEVVTDWRLLPTEALVHCRTRSWVSFGLVHATNATLAFLCFLGTFLVQSRPGHYNRARGLTFAMLAYFITWVSFVPLLANVQVVLRPAVQMGALLL
Rhinopithecus bieti	VAFPPEVVTDWHMLPTEALVHCRTRSWVSFGLVHATNATLAFLCFLGTFLVQSRPGRYNRARGLTFAMLAYFITWVSFVPLLANVQVVLRPAVQMGALLL
Rhinopithecus brelichi	VAFPPEVVTDWHMLPTEALVHCRTRSWVSFGLVHATNATLAFLCFLGTFLVQSRPGRYNRARGLTFAMLVYFITWVSFVPLLANVQVVLRPAVQMGALLL
Rhinopithecus roxellana	VAFPPEVVTDWHMLPTEALVHCRTRSWVSFGLVHATNATLAFLCFLGTFLVQSRPGRYNRARGLTFAMLAYFITWVSFVPLLANVQVVLRPAVQMGALLL
Trachypithecus francoisi	VAFPPEVVTDWRMLPTEALVHCRTRSWVSFGLVHATNATLAFLCFLGTFLVQSRPGRYNRARGLTFAMLAYFITWVSFVPLLANVQVVLRPAVQMGALLL
Semnopithecus vector	VAFPPEVVTDWRMLPTEALVHCRTRSWVSFGLVHATNATLAFLCFLGTFLVQSRPGRYNRARGLTFAMLAYFITWVSFVPLLANVQVVLRPAVQMGALLL
Presbytis melalophos	VAFPPEVVTDWRMLPTEALVHCRTRSWVSFGLVHATNATLAFLCFLGTFLVQSRPGRYNRARGLTFAMLAYFITWVSFVPLLANVQVVLRPAVQMGALLL
Colobus polykomos	VAFPPEVVTDWRMLPTEALVHCRTRSWVSFGLVHATNATLAFLCFLGTFLVQSRPGRYNRARGLTFAMLAYFITWVSFVPLLANVQVVLRPAVQMGALLL
Hoolock hoolock	VAFPPEVVTDWRMLPKEALVHCRTRSWVSFGLAHATNATLAFLCFLGTFLVQSQPGRYNRARGLTFAMLAYFITWVSFVPLLANVQVVLRPAVHMGALLL
Symphalangus syndactylus	VAFPPEVVTDWRMLPKEALVHCRTRSWVSFGLAHATNATLAFLCFLGTFLVQSQPGRYNRARGLTFAMLAYFITWVSFVPLLANVQVVLRPAVHMGALLL
Hylobates pileatus	VAFPPEVVTDWRMLPKEALVHCRTRSWVSFGLAHATNATLAFLCFLGTFLVQSQPGRYNRARGLTFAMLAYFITWVSFVPLLANVQVVLRPAVHMGALLL
Hylobates lar	VAFPPEVVTDWRMLPKEALVHCRTRSWVSFGLAHATNATLAFLCFLGTFLVQSQPGRYNRARGLTFAMLAYFITWVSFVPLLANVQVVLRPAVHMGALLL
Hylobates abbotti	VAFPPEVVTDWRMLPKEALVHCRTRSWVSFGLAHATNATLAFLCFLGTFLVQSQPGRYNRARGLTFAMLAYFITWVSFVPLLANVQVVFRPAVHMGALLL
Hylobates agilis	VAFPPEVVTDWRMLPKEALVHCRTRSWVSFGLAHATNATLAFLCFLGTFLVQSQPGRYNRARGLTFAMLAYFITWVSFVPLLANVQVVFRPAVHMGALLL
Nomascus annamensis	VAFPPEVVTDWRMLPKEALVHCRTRSWVSFGLAHATNATLAFLCFLGTFLVQSQPDRYNRARGLTFAMLAYFITWVSFVPLLANVQVVLRPAVHMGALLL
Nomascus leucogenys	VAFPPEVVTDWRMLPKEALVHCRTRSWVSFGLAHATNATLAFLCFLGTFLVRSQPGRYNRARGLTFAMLAYFITWVSFVPLLANVQVVLRPAVHMGALLL
Pan troglodytes	VAFPPEVVTDWHMLPTEALVHCRTRSWVSFGLAHATNATLAFLCFLGTFLVRSQPGRYNRARGLTFAMLAYFITWVSFVPLLANVQVVLRPAVQMGALLL
Homo sapiens	VAFPPEVVTDWHMLPTEALVHCRTRSWVSFGLAHATNATLAFLCFLGTFLVRSQPGRYNRARGLTFAMLAYFITWVSFVPLLANVQVVLRPAVQMGALLL
Gorilla gorilla gorilla	VAFPPEVVTDWHMLPTEALVHCRTRSWVSFGLAHATNATLAFLCFLGTFLVRSQPGRYNRARGLTFAMLAYFITWVSFVPLLANVQVVLRPAVQMGALLL
Pongo abelii	VAFPPEVVTDWHILPTEALVHCRTRSWVSFGLAHATNATLAFLCFLGTFLVQSRPGRYNRARGLTFAMLAYFITWVSFVPLLANVQVVLRPAVQMGALLL
Pongo pygmaeus	VAFPPEVVTDWHILPTEALVHCRTRSWVSFGLAHATNATLAFLCFLGTFLVQSRPGRYNRARGLTFAMLAYFITWVSFVPLLANVQVVLRPAVQMGALLL
Lophocebus aterrimus	VAFPPEVVTDWRMLPTEALVHCRARSWVSFGLVHATNATLAFLCFLGTFLVQSRPGRYNRARGLTFAMLAYFITWVSFVPLLANVQVVLRPAVQMGALLL

	801–852
Theropithecus gelada	CVLGILAAFHLPRCYLLVRQPELNTPEFFLGRGPGDARDRNDGDTGNQGKHE
Papio hamadryas	CVLGILAAFHLPRCYLLVRQPELNTPEFFLGRGPGDARDRNDGDTGNQGKHE
Cercocebus chrysogaster	CVLGILAAFHLPRCYLLVRQPELNTPEFFLGRGPGDARDRNDGHTGNQGKHE
Mandrillus sphinx	CVLGILAAFHLPRCYLLVRQPELNTPEFFLGRGPGDARDRNDGHTGNQGKHE
Macaca assamensis	CVLGILAAFHLPRCYLLVQQPELNTPEFFLGRGPGDARDRNDGDTGNQGKHE
Macaca arctoides	CVLGILAAFHLPRCYLLVRQPELNTPEFFLGRGPGDARDRNDGDTGNQGKHE
Macaca fuscata	CVLGILAAFHLPRCYLLVRQPELNTPEFFLGRGPGDARDRNDGDTGNQGKHE
Cercopithecus mitis	CVLGILAAFHLPRCYLLVRQPELNTPEFFLGRGPGDARDRNDGDTGNQGKHE
Cercopithecus albogularis	CVLGILAAFHLPRCYLLVRQPELNTPEFFLGRGPGDARDRNDGDTGNQGKHE
Erythrocebus patas	CVLGILAAFHLPRCYLLVRQPELNTPEFFLGRGPGDARDRNDGDTGNQGNHE
Chlorocebus sabaceus	CVLGILAAFHLPRCYLLVRQPELNTPEFFLGRGPGDARDRNDGDTGNQGKHE
Nasalis larvatus	CVLGILAAFHLPRCYLLVRQPELNTPEFFLGRGPGDARDRNDGDTGNQGKPE
Pygathrix nemaeus	CVLGILAAFHLPRCYLLVRQPELNTPEFFLGRGPGDARERNDGDTGNQGKPE
Pygathrix nigripes	CVLGILAAFHLPRCYLLVRQPELNTPEFFLGRGPGDARERNDGDTGNQGRPE
Rhinopithecus bieti	CVLGILAAFHLPRCYLLVRQPELNTPEFFLGRGPGDARDRNDGDTGNQGKPE
Rhinopithecus brelichi	CVLGILAAFHLPRCYLLVRQPELNTPEFFLGRGPGDARDRNDGDTGNQGKPE
Rhinopithecus roxellana	CVLGILAAFHLPRCYLLVRQPELNTPEFFLGRGPGDARDRNDGDTGNQGKPE
Trachypithecus francoisi	CVLGILAAFHLPRCYLLVRQPELNTPEFFLGRGPGDARDRNDGDTGN?GKPE
Semnopithecus vector	CVLGILAAFHLPRCYLLVRQPELNTPEFFLGRGPGDARDRNDGDTGN?GKPE
Presbytis melalophos	CVLGILAAFHLPRCYLLVRQPELNTPEFFLGRGPGDARDRNDGDTGNQGKPE
Colobus polykomos	CVLGILAAFHLPRCYLLVRQPELNTPEFFLGRGPGDARDRNDADTGNQGKPE
Hoolock hoolock	CVLGILAAFHLPRCYLLMQQPGLNTPKFFLGGGPGDAQGRNDGDTGNQGKHE
Symphalangus syndactylus	CVLGILAAFHLPRCYLLMQQPGLNTPEFFLGGGPGDAQGRNDGDTGNQGKHE
Hylobates pileatus	CVLGILAAFHLPRCYLLMQQPGLNTPEFFLGGGPGDAQGRNDGDTGNQGKHE
Hylobates lar	CVLGILAAFHLPRCYLLMQQPGLNTPEFFLGGGPGDAQGRNDGDTGNQGKHE
Hylobates abbotti	CVLGILVAFHLPRCYLLMQQPGLNTPEFFLGGGPGDAQGRNDGDTGNQGKHE
Hylobates agilis	CVLGILAAFHLPRCYQLMQQPGLNTPEFFLGGGPGDAQGRNDGDTGNQGKHE
Nomascus annamensis	CVLGILAAFHLPRCYLLMQQPGLNTPEFFLGGGPGDAQGRNNGDTGNQGKHE
Nomascus leucogenys	CVLGILAAFHLPRCYLLMQQPGLNTPEFFLGGGPGDAQGRNDGDTGNQGKHE
Pan troglodytes	CVLGILAAFHLPRCYLLMWQPGLNTPEFFLGGGPGDAQGRNDGDTGNQGKHE
Homo sapiens	CVLGILAAFHLPRCYLLMRQPGLNTPEFFLGGGPGDAQGQNDGNTGNQGKHE
Gorilla gorilla gorilla	CVLGILAAFHLPRCYLLIRQPGLNTPEFFLGGGPGDAQGRNDGDTGNQGKHE
Pongo abelii	CVLGILAAFHLPRCYLLMRQPGLNTPEFFLGGGPGDAQGRNDGDTGNQRKHE
Pongo pygmaeus	CVLGILAAFHLPRCYLLMRQPGLNTPEFFLGGGPGDAQGRNDGDTGNQRKHE
Lophocebus aterrimus	CVLGILAAFHLPRCYLLVRQPELNTPEFFLGRGPGDA*DRNDGDTGNQGKHE
