# Supplementary figures and images for: Differentiated adaptive evolution, episodic relaxation of selective constraints, and pseudogenization of umami and sweet taste genes TAS1Rs in catarrhine primates
Source: Front Zool. 2014 Oct 29;11:79. doi: 10.1186/s12983-014-0079-4 (PMC4226867; doi:10.1186/s12983-014-0079-4)

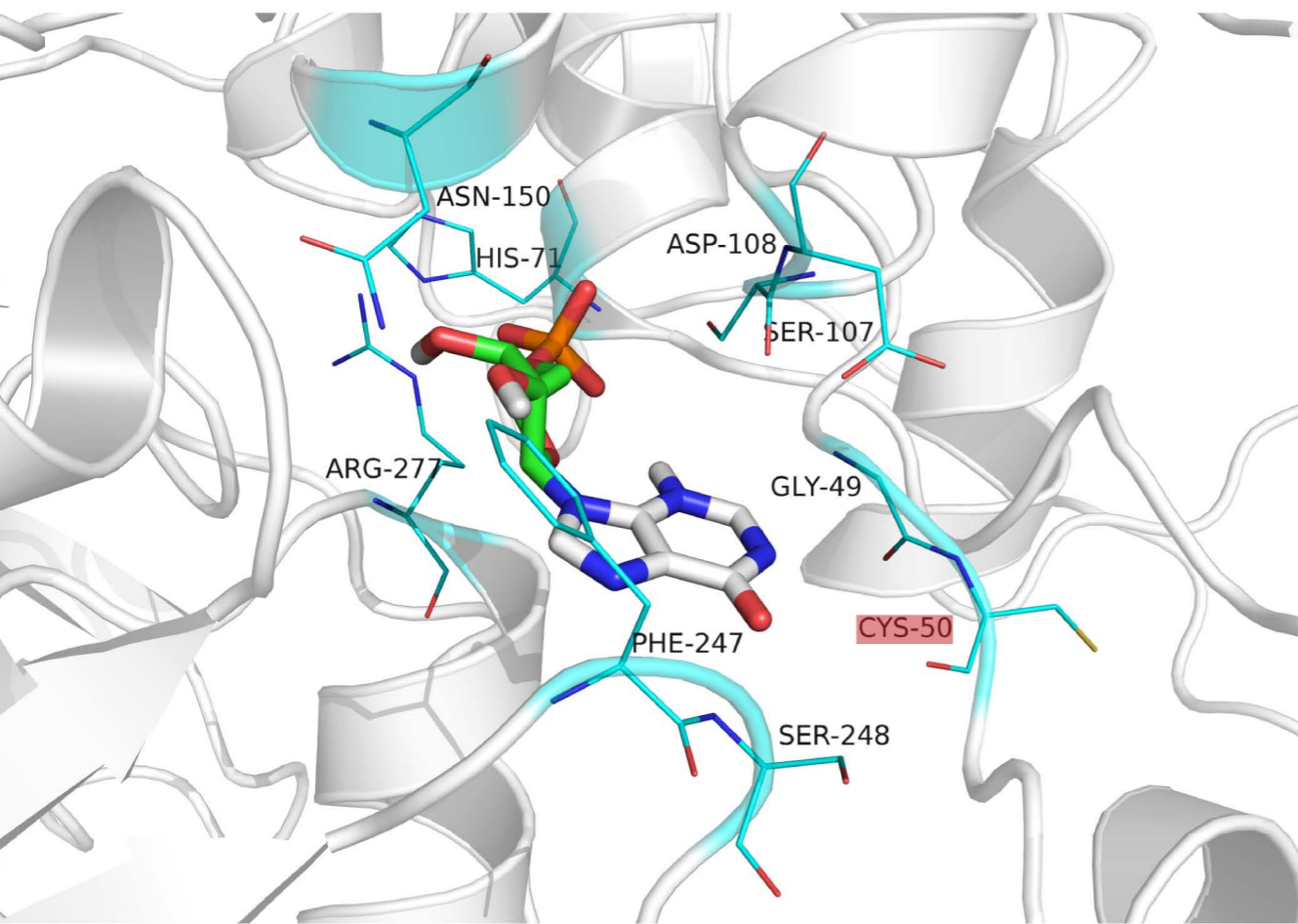

IMP

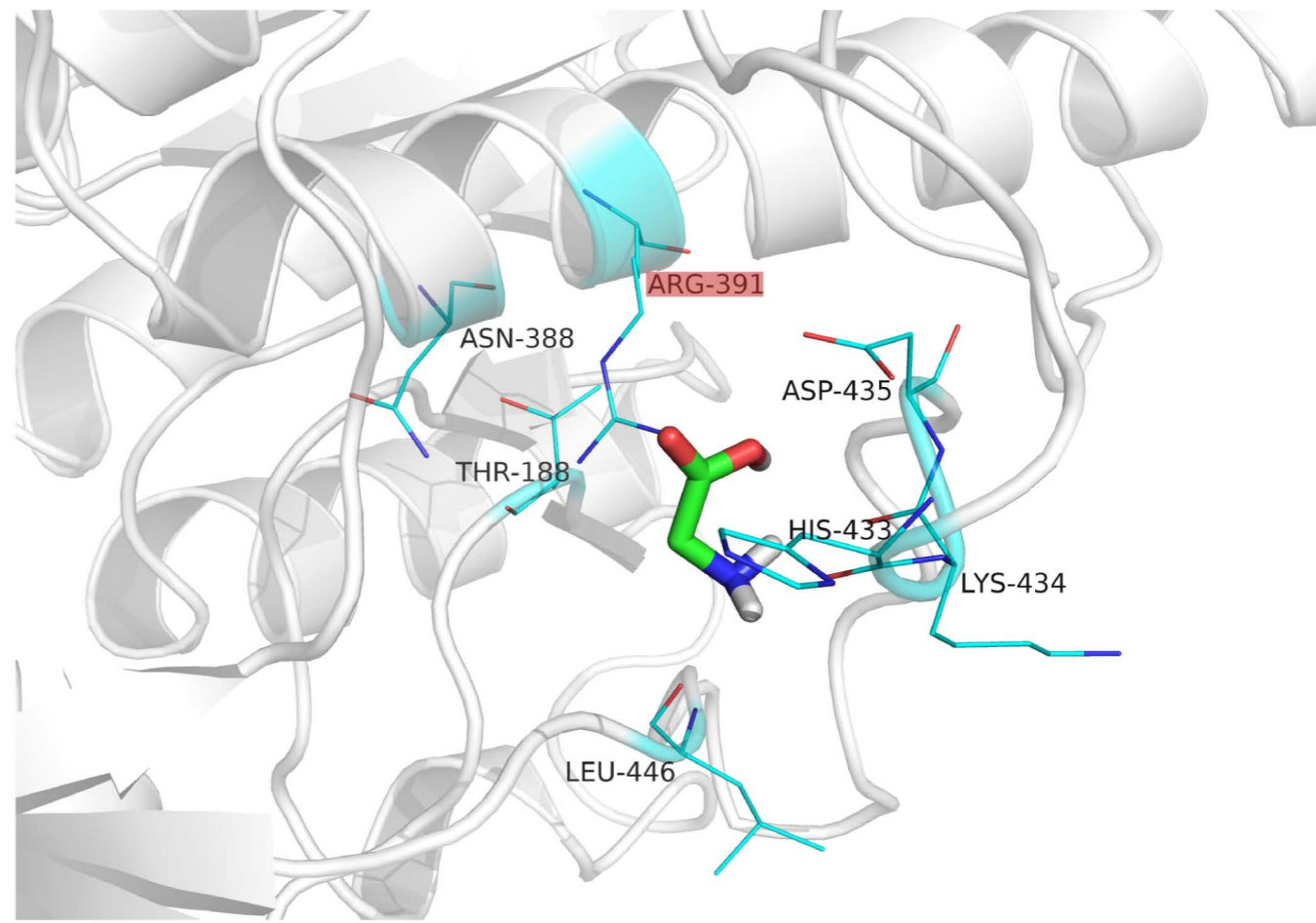

glycine

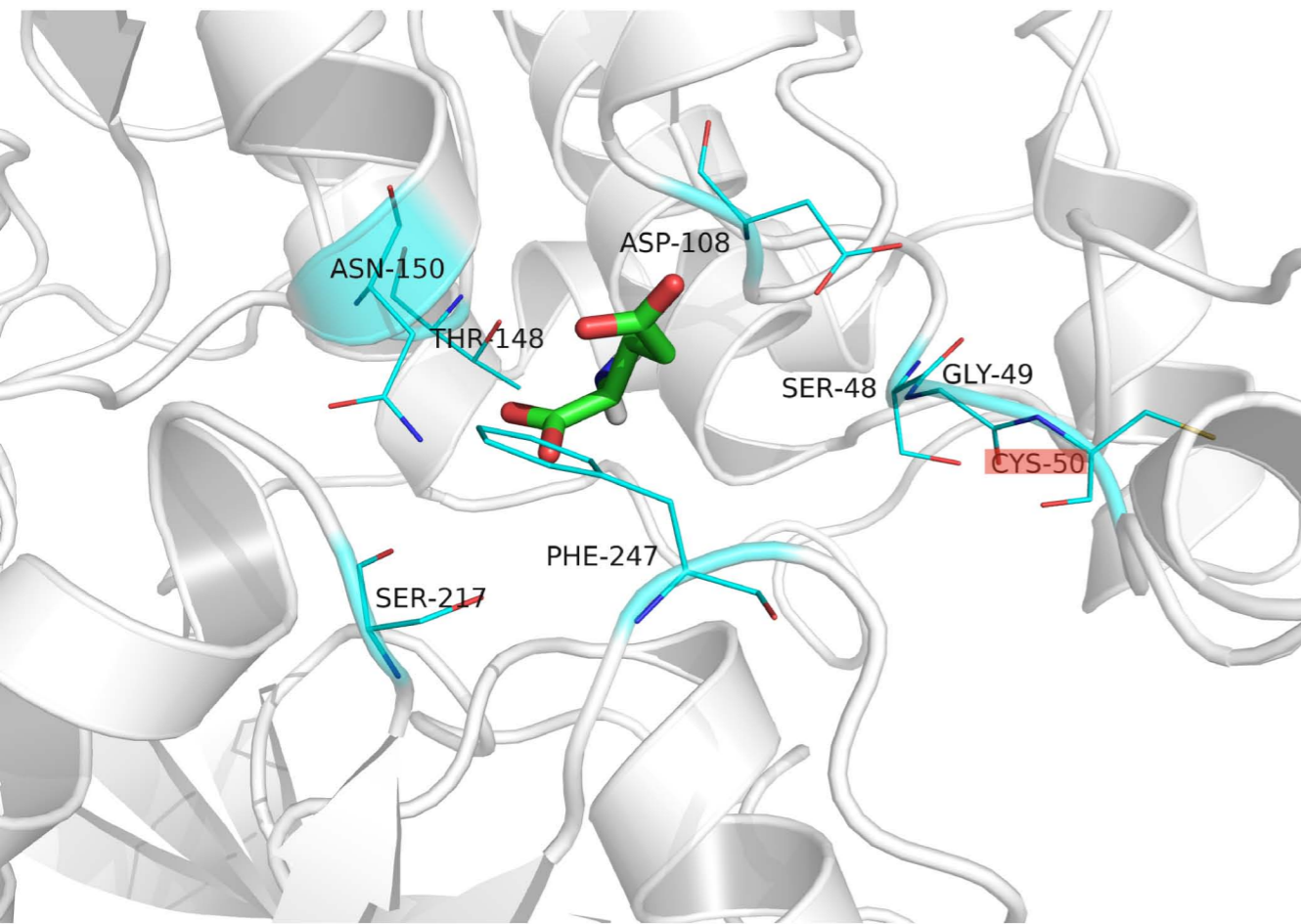

glutamate

Supplement: Additional file 7: — Molecular docking results of TAS1R1. Positive selection sites detected in our research are marked with red. [file 12983_2014_79_MOESM7_ESM.pdf]

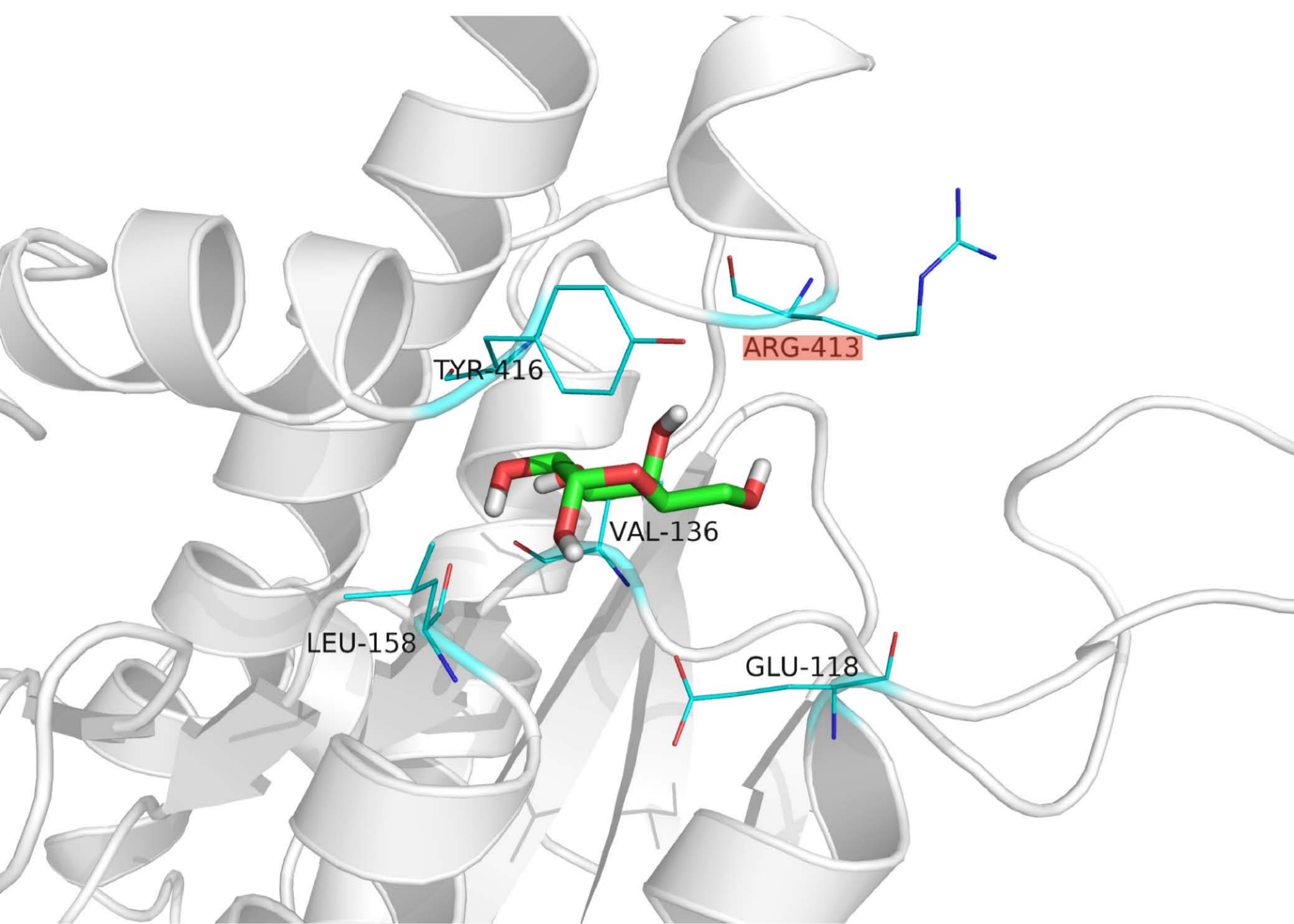

D-galactose

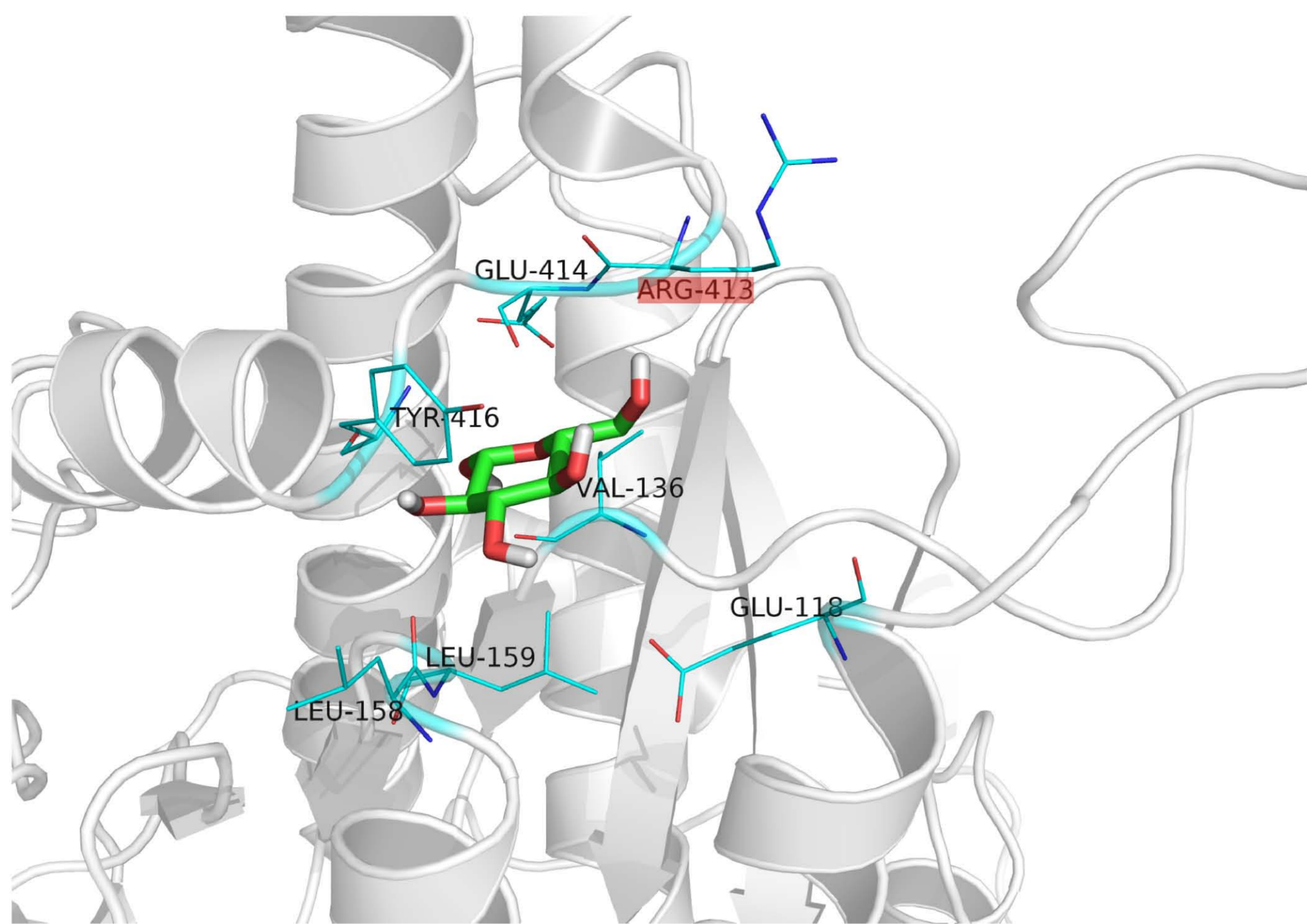

D-glucose

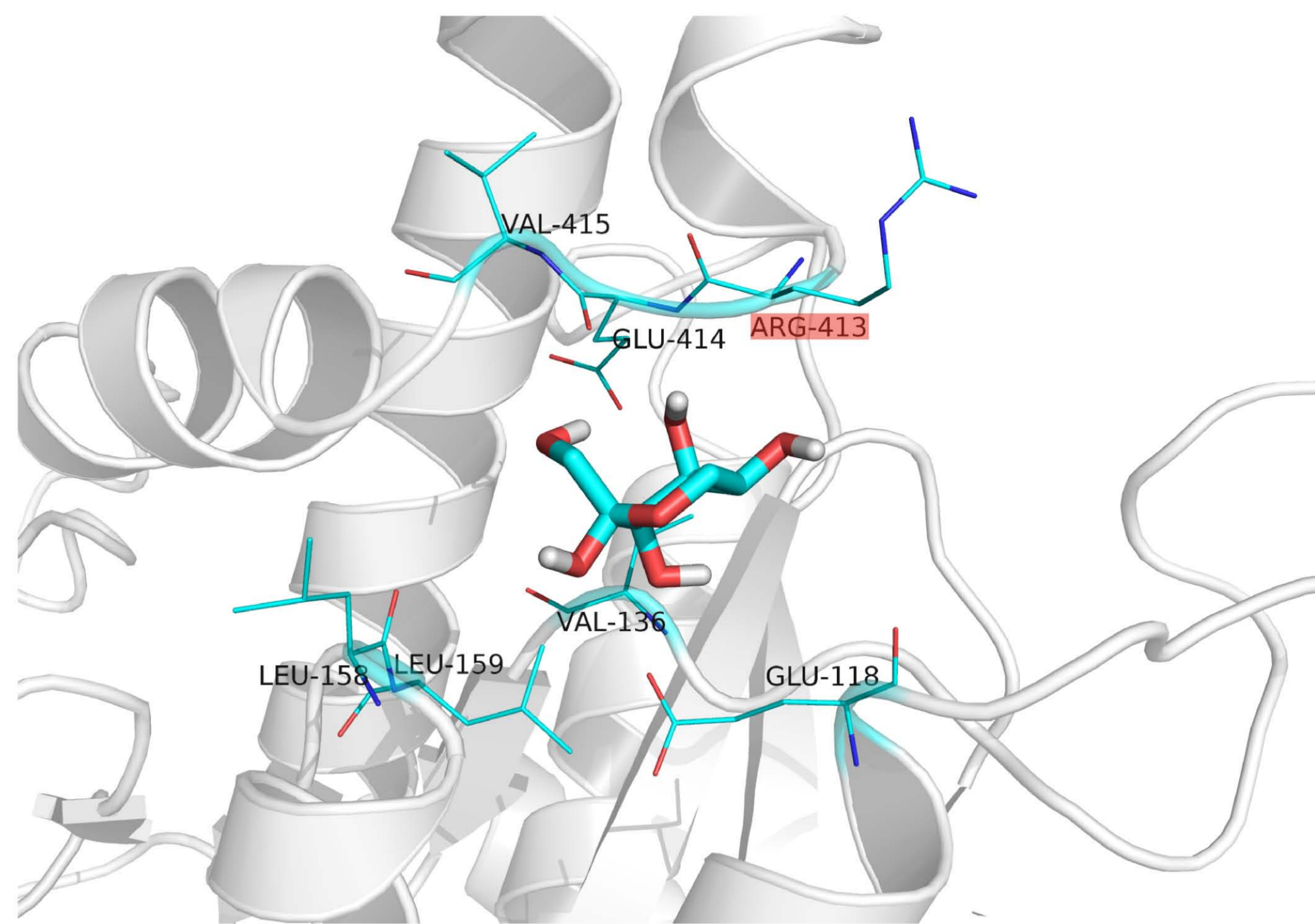

fructose

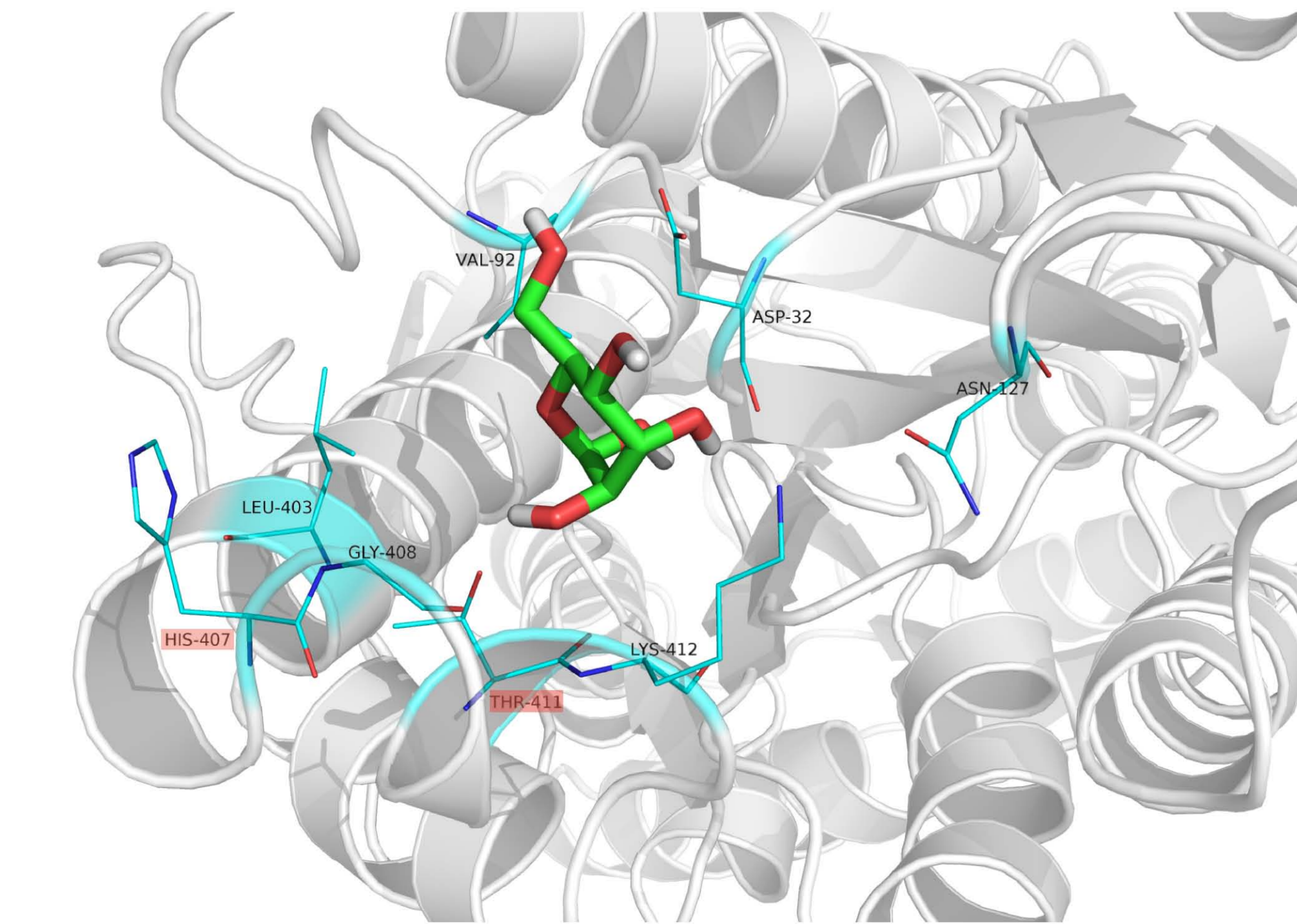

galactose

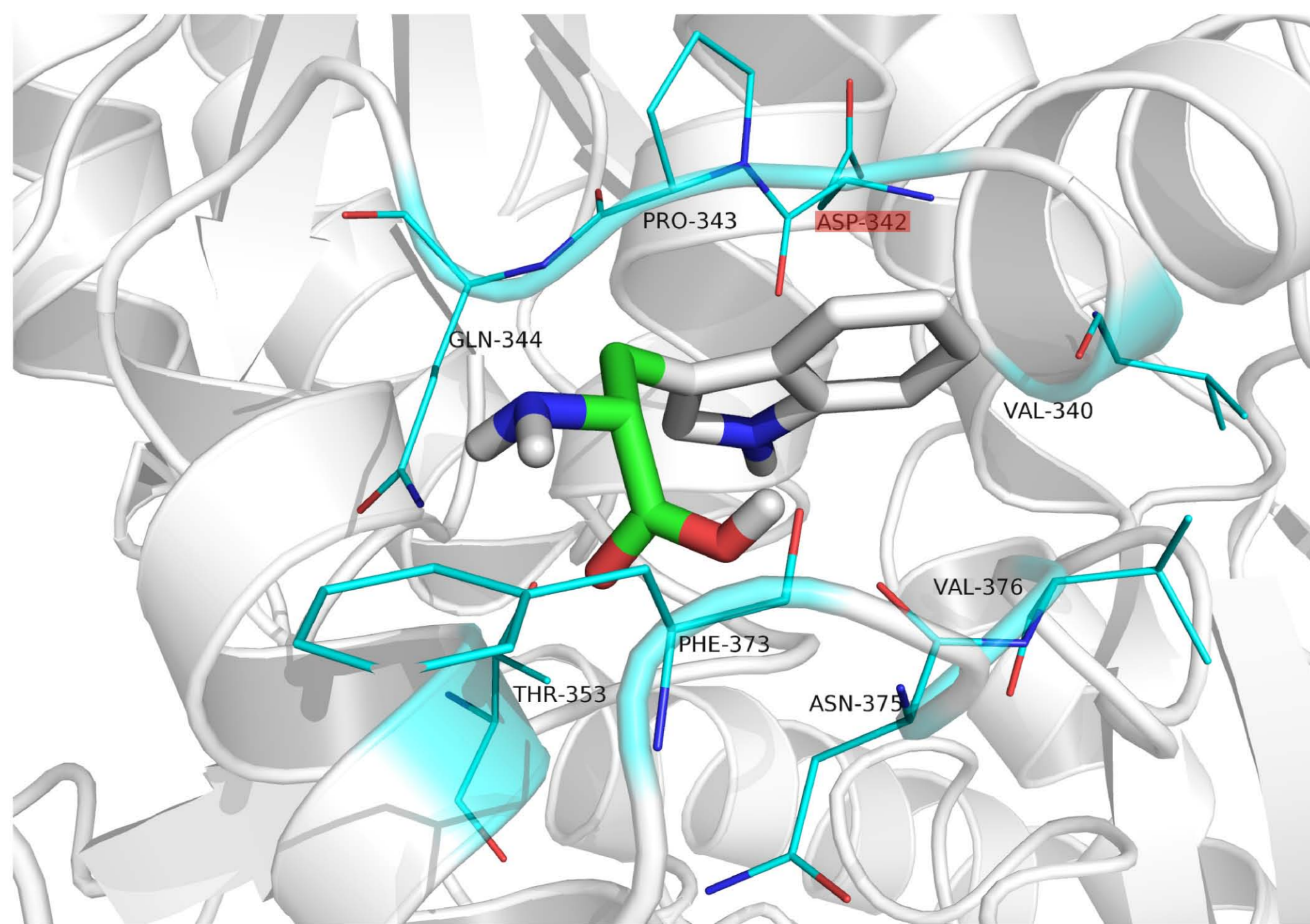

D-tryptophan

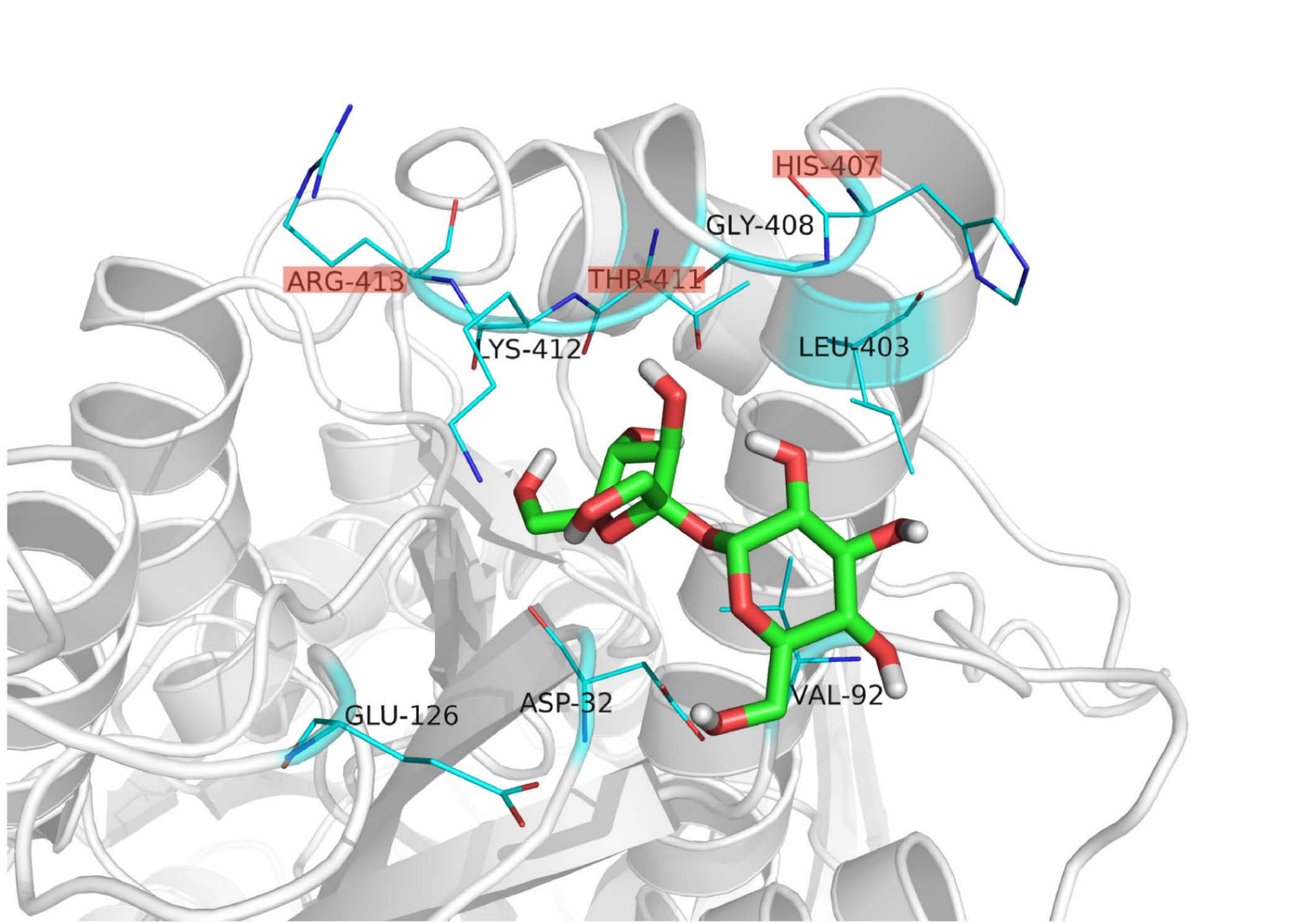

sucrose

Supplement: Additional file 8: — Molecular docking results of TAS1R2. Positive selection sites detected in our research are marked with red. [file 12983_2014_79_MOESM8_ESM.pdf]
